# METTL14-dependent *N*^6^-methyladenosine modification of FGF16 participates in IGFBP5 deficiency-enhanced angiogenesis

**DOI:** 10.1038/s12276-026-01788-y

**Published:** 2026-08-04

**Authors:** Fei Song, Yu Hu, Yi-Xiang Hong, Shan-Shan Zhao, Hui-Zhu Huang, Yutian Wang, Le Zhang, Wei-Yin Wu, Yan Wang, Gang Li

**Affiliations:** 1 Xiamen Cardiovascular Hospital of Xiamen University, School of Medicine, Xiamen University, Xiamen, Fujian 361009 China; 2 Department of Cardiology, Sir Run Run Shaw Hospital, Zhejiang University School of Medicine, Hangzhou, Zhejiang 310016 China; 3 Department of Cardiology, Nanfang Hospital, Southern Medical University, Guangzhou, Guangdong 510515 China

**Keywords:** Peripheral vascular disease, Cell growth

## Abstract

*N*^6^-methyladenosine (m^6^A) RNA modification plays critical roles in physiological and pathological processes. Our prior study demonstrated that IGFBP5 expression is upregulated in the ischemic limb, whereas endothelial-specific IGFBP5 knockout (Igfbp5^EKO^) protects against hind limb ischemia by enhancing angiogenesis. Here, we show that IGFBP5 deficiency elevates global m^6^A levels and upregulates the expression of m^6^A methyltransferase complex components METTL3, METTL14 and WTAP in endothelial cells. We further identified a direct interaction between IGFBP5 and the MT-A70 domain of METTL14. Knockdown of METTL14, METTL3 or WTAP attenuated the pro-angiogenic effects of IGFBP5 deficiency in vitro. In vivo, endothelial knockdown of METTL14 abolished the improved hind-limb ischemia recovery in Igfbp5^EKO^ mice. Methylated RNA immunoprecipitation sequencing revealed that IGFBP5 depletion in endothelial cells increases both m^6^A modification and mRNA abundance of FGF16. Notably, METTL14 or METTL3 silencing suppressed IGFBP5-dependent FGF16 upregulation. Enhanced translational efficiency of FGF16 in IGFBP5-deficient cells was reversed by METTL14 knockdown, indicating that IGFBP5 regulates FGF16 translation via m^6^A modification. Mechanistically, IGFBP5 modulates FGF16 m^6^A modification via interaction with the m^6^A reader protein IGF2BP2, targeting the m^6^A site at position 255 of FGF16 mRNA. In summary, our study establishes METTL14-mediated m^6^A modification of FGF16 as a key mechanism underlying IGFBP5-driven angiogenesis. Targeting the IGFBP5–METTL14–m^6^A–FGF16 axis may offer novel therapeutic strategies for ischemic disease.

**Figure:**
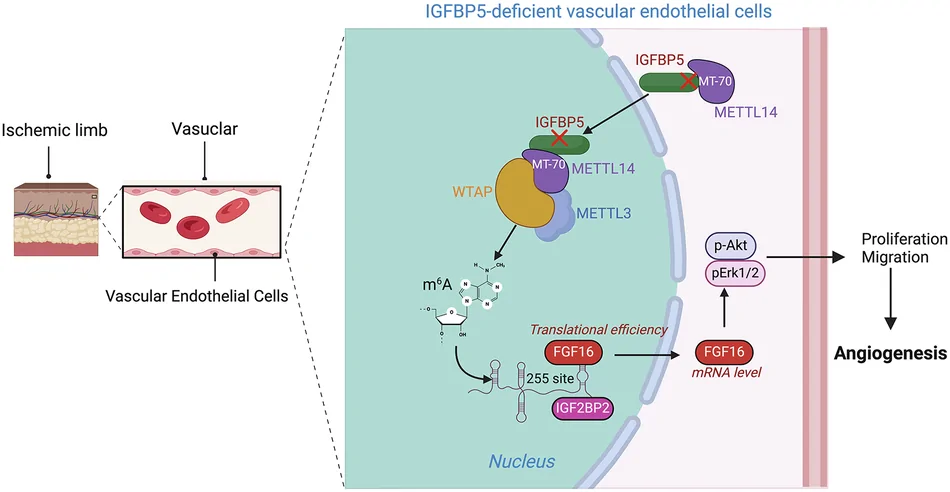

## IGFBP5 Deficiency Boosts Angiogenesis via m6A Modification

Ischemic diseases, such as peripheral arterial disease and myocardial infarction, are increasingly prevalent owing to ageing populations and rising metabolic disorders. This study explores the role of IGFBP5 in angiogenesis, focusing on non-IGF-dependent mechanisms involving m^6^A RNA modification. Researchers knocked down IGFBP5 in human umbilical vein endothelial cells and observed increased m^6^A levels and upregulation of m^6^A writers such as METTL14. IGFBP5 interacts with the MT-A70 domain of METTL14, influencing m^6^A modification and promoting angiogenesis. In vivo, METTL14 knockdown in IGFBP5-deficient mice reversed improved blood-flow recovery after ischemic injury. The study highlights FGF16 as a key target of IGFBP5-mediated m^6^A modification, enhancing its stability and translation via IGF2BP2. These findings suggest that targeting the IGFBP5–METTL14–FGF16 axis could offer new therapeutic strategies for ischemic diseases, with future research needed to explore its potential in larger animal models.

## Introduction

The prevalence of ischemic diseases, including peripheral arterial disease (PAD) and myocardial infarction, continues to rise globally, driven by the ageing population and the increasing incidence of metabolic disorders^1,2^. Patients with PAD often exhibit symptoms of critical limb ischemia (CLI), and some require lower-limb amputation as a treatment^3,4^. The primary pathogenic feature of ischemic diseases is blood vessel blockage, exacerbated by inadequate angiogenesis^5,6^. Despite therapeutic advances, revascularization remains the standard treatment^7,8^. Identifying key molecules and angiogenesis pathways is critical for developing effective pharmacological strategies. Our previous study demonstrated that IGFBP5 deficiency confers therapeutic benefits in hind-limb ischemia (HLI) via IGF-dependent mechanism^9^. However, whether IGFBP5-regulated angiogenesis involves non-IGF-dependent mechanisms, such as *N*^6^-methyladenosine (m^6^A) modification, remains unexplored.

m^6^A, the most prevalent internal modification in eukaryotic messenger RNAs (mRNAs), plays critical roles in numerous biological processes^10^. It is crucial in multiple fields such as developmental control^11^, the cell cycle^12^, fate determination^13,14^ and the heat-shock stress response^15^, by influencing RNA metabolism, including pre-mRNA processing, translation efficiency, transcript stability and miRNA production^16–20^. The level of m^6^A modification is dynamically regulated by “writers” and “erasers”, whereas its effects are mediated by “readers”^21–23^. The writer complex typically consists of two proteins, methyltransferase-like 3 (METTL3) and methyltransferase-like 14 (METTL14). METTL3 is the critical RNA methyltransferase, whereas METTL14 assists in RNA binding to support METTL3^24,25^. Recently, there have been notable advancements in understanding the biochemistry and molecular controls of m^6^A methylation in many physiological processes. However, the functional significance of m^6^A in pathological angiogenesis, the process of new blood-vessel formation, which is crucial in conditions such as development, tissue ischemia, diabetes and cancer, remains underexplored. It has been demonstrated that depletion of METTL3 decreases the angiogenic abilities of endothelial cells, including viability, proliferation, migration and tube formation^26^. Our earlier research has shown that the removal of IGFBP5 increases the formation of blood vessels by stimulating ATP metabolism and stabilizing HIF1ɑ^9^. In the current study, we revealed that suppressing IGFBP5 not only enhanced the expression of m^6^A writers (METTL3, METTL14 and WTAP) but also raised levels of cellular m^6^A modification, suggesting that m^6^A methylation may play an essential role in IGFBP5-regulated endothelial-cell activities. Further analysis revealed that IGFBP5 binds to METTL14 via the MT-A70 domain. Additionally, we found that the METTL14–m^6^A axis influenced IGFBP5-mediated restoration of blood flow and angiogenesis after ischemic injury in mice. Moreover, we demonstrated that FGF16 is the target of IGFBP5-mediated m^6^A modification through its binding to the m^6^A reader IGF2BP2, specifically at the 255 m^6^A site.

## Methods

### Reagents and antibodies

The detailed information of reagents and antibodies is shown in the Supplementary Materials.

### Cell culture and transfection

Human umbilical vein endothelial cells (HUVECs) were obtained from the American Type Culture Collection (Virginia, MA, USA). HUVECs were grown in ECM medium supplemented with 5% fetal bovine serum (FBS), 1% endothelial-cell growth supplement and 1% penicillin/streptomycin (ScienCell, USA) in a 5% CO₂ incubator at 37 °C. After 72 h of transduction with IGFBP5-shRNA (Cat#: sc-39591-V, Santa Cruz) and control shRNA (Cat#: sc-108060, Santa Cruz) lentivirus, the cells were co-transfected with Lipofectamine RNAi MAX and control siRNA, METTL3 siRNA, METTL14 siRNA, WTAP siRNA and FGF16 siRNA (sequences are shown in Supplementary Table 1) at a final concentration of 100 nM. Cells were then used for detection or collected 48–60 h after transfection.

### Construction of the plasmid of IGFBP5 and different domains of METTL14

To determine the interaction domain of METTL14 with IGFBP5, we constructed four plasmids based on the pEGFP-C1 vector: (1) wild-type Flag-METTL14 (full-length METTL14, 1–456 amino acids, wild type), (2) Flag-ΔN-MT-A70 (deletion of 1–185 amino acids, N-terminal before MT-A70), (3) Flag-ΔMT-A70 (deletion of 186–363, MT-A70 domain), and (4) Flag-ΔC-MT-A70 (deletion of 364–456 amino acids, C-terminal post MT-A70). The METTL14 plasmids were tagged with FLAG at the N-terminus, whereas the IGFBP5 plasmid was tagged with HA at the C-terminus. All plasmids were commercially synthesized by Sangon Biotech (Shanghai, China).

### m^6^A RNA methylation quantification

The EpiQuik m^6^A RNA Methylation Quantification Kit (Cat#: P-9005, EpigenTek, Farmingdale, NY, USA) was applied to determine the m^6^A levels in the HUVECs that were infected with control shRNA and IGFBP5 shRNA. In brief, after total RNA isolation from cells, 96-well plates were coated with 80 μl of binding solution and 200 ng of total RNA. The plates were then incubated at 37 °C for 90 min. After washing, 50 μl of diluted capture antibody was added, followed by incubation for 60 min. Subsequently, samples were treated with detection antibody (50 μl) and diluted enhancer solution (50 μl) separately for 30 min. Following a 10-min incubation with the developer solution, the reaction was halted by adding the stop solution. Absorbance was measured at 450 nm using a microplate reader within 3–4 min. The m^6^A level was calculated using the formula m^6^A% = (OD_Sample_ – ODNegative) / (OD_Positive control_ – OD_Negative control_) x 100%.

### m^6^A methylated RNA immunoprecipitation quantitative PCR

Total RNA was extracted from HUVECs and treated with DNase (Sigma-Aldrich, St. Louis, MO, USA) to remove genomic DNA. Methylated RNA immunoprecipitation quantitative PCR was performed using the Magna RIP RNA-Binding Protein Immunoprecipitation Kit (Cat#: 17-700, Millipore, Burlington, MA, USA) following the manufacturer’s protocol. Cells were lysed in RIPA buffer, and RNA samples were immunoprecipitated with an anti-m^6^A antibody. The abundance of m^6^A-modified mRNA was quantified using reverse transcription quantitative PCR.

### Mice and animal housing

The mice study was carried out in accordance with the guidelines from Directive 2010/63/EU of the European Parliament on the protection of animals used for scientific purposes or the NIH Guide for the Care and Use of Laboratory Animals. Endothelial-specific Igfbp5 knockout (Igfbp5^EKO^) mice on a C57BL/6 background were generated and provided by GemPharmatech Co. Ltd. (Nanjing, China). The knockout efficiency was validated in our previous study^9^. Male mice aged 6–8 weeks were housed under standard conditions in the animal facility of Xiamen University. The study was approved by the Institutional Animal Care and Use Committee of Xiamen University (Approval No. XMULAC20230205).

### Hind-limb ischemia and the blood-flow recovery scan

Mice were anaesthetized with 3% isoflurane. The femoral artery was surgically exposed and isolated from the adjacent nerve. The segment between the profunda femoris artery and saphenous artery branches was ligated and excised. The incision was closed with a single-layer 4–0 Prolene suture. Blood flow was measured using a laser Doppler perfusion imaging system (PeriScan PIM 3, Perimed, Järfälla, Sweden) preoperatively (day −1) and on postoperative days 0, 7, 14, 21 and 28. Perfusion ratios were calculated as ischemic/non-ischemic limb flux units. Mice were euthanized by CO₂ asphyxiation.

### Gastrocnemius injection with adeno-associated virus and adenovirus

Adeno-associated virus (AAV) was obtained from Hanbio Co., Ltd. (Shanghai, China). For METTL14 knockdown, 200 μl of HB-AAV2/9-Mettl14-TIE-m-ZsGreen or control HB-AAV2/9-TIE-ZsGreen controls 2/9 at a concentration of 1 × 10^12^ vg/ml was injected into the gastrocnemius muscle of wild type or Igfbp5^EKO^ mice. AAV injections were performed 3 weeks prior to HLI induction.

### Methylated RNA immunoprecipitation sequencing

Methylated RNA immunoprecipitation sequencing was performed by Aksomics Biotech Inc. (Shanghai, China). Total RNA was extracted from HUVECs transfected with control shRNA or IGFBP5 shRNA using TRIzol reagent. The total mRNA was purified with the Dynabeads mRNA DIRECT Kit (Cat#: 61011, Thermo Fisher Scientific, Waltham, MA, USA), and fragmentation was induced by sonication (10 ng/ml in 100 ml RNase-free water). Fragmented mRNA was incubated with anti-m^6^A immunoprecipitate (IP) antibody (Synaptic Systems, Cat#: 202003) in 1x IP buffer for 2 h at 4 °C under gentle rotation. Protein A magnetic beads (Thermo Fisher Scientific), pre-washed twice with IP buffer, were added to the mixture and incubated at 4 °C for 2 h with continuous mixing. Beads were washed three times with IP buffer, and bound RNA was eluted and purified using an RNA Clean & Concentrator Kit (Zymo Research, Orange, CA, USA). Sequencing libraries were prepared with the TruSeq Stranded mRNA Library Prep Kit (Illumina, San Diego, CA, USA) and sequenced on an Illumina HiSeq 2000 platform. Reads were aligned to the human reference genome (GRCh38) using TopHat, and differential m^6^A peaks between IP and input samples were identified with exomePeak (*P* < 0.01).

### ZDOCK structure prediction

The molecular interaction between IGFBP5 and METTL14 was predicted using ZDOCK software (http://zdock.umassmed.edu). Structural coordinates of IGFBP5 and METTL14 domains were obtained from the RCSB Protein Data Bank (PDB ID: 3V2A). Three-dimensional models were visualized using PyMOL (version 2.5.4, Schrödinger, New York, NY, USA).

### Matrigel tube formation assay

96-well plates were pre-coated with 50 µl of Matrigel Growth Factor Reduced Matrix (Corning, Cat#: 356230) and incubated at 4 °C for 15 min, followed by polymerization at 37 °C for 60 min. HUVECs were serum-starved for 12 h, trypsinized, resuspended in ECM medium containing 1% FBS and seeded onto the Matrigel-coated wells at a density of 2.0 × 10⁴ cells per well. After 6 h of incubation, tube formation was visualized under a phase-contrast microscope (Nikon, Japan). Quantitative analysis of total tube length, branch length and segment length was performed using ImageJ software (NIH, USA) with the Angiogenesis Analyzer plugin.

### Cell-cycle analysis

Cell-cycle distribution was analysed by flow cytometry using propidium iodide staining. Briefly, transfected HUVECs were trypsinized with 0.25% trypsin-EDTA (Thermo Fisher Scientific), fixed in ice-cold 70% ethanol, and stored at 4 °C overnight. Cells were washed twice with PBS and stained with a solution containing 20 μg/ml propidium iodide (Cat#: P1304MP, Thermo Fisher Scientific, Waltham, MA, USA), 10 μg/ml RNase A and 0.1% Triton X-100 in PBS for 30 min at room temperature. DNA content was measured using a BD LSR Fortessa flow cytometer (BD Biosciences, NJ, USA), and cell-cycle phases (G0/G1, S, G2/M) were quantified using ModFit LT software (Verity Software House, ME, USA).

### Transwell assay

HUVEC migration was assessed using a Transwell chamber system with an 8-μm pore polycarbonate membrane (Cat: 3422, Corning Costar, NY, USA). Briefly, 2 × 10^4^ cells (passages 3–8) were resuspended in 200 μl of ECM containing 1% FBS and seeded into the upper chamber. The lower chamber was filled with 600 μl of ECM supplemented with 10% FBS as a chemoattractant. After 48 h of incubation at 37 °C, non-migrated cells on the upper membrane surface were removed using a cotton swab. Migrated cells were fixed with 4% paraformaldehyde for 15 min, stained with 0.1% crystal violet (Sigma-Aldrich, Cat#: C3886) for 20 min, and imaged using an Olympus IX73 phase-contrast microscope. Five randomly selected fields per insert were analysed using ImageJ software (NIH, USA).

### Quantitative real-time PCR

Total RNA was isolated from HUVECs using TRIzol reagent (Cat#: 15596026, Life Technology, CA, USA). First-strand cDNA was synthesized from 1 μg of RNA with HiScript III All-in-One RT SuperMix Perfect (Cat#: R333, Vazyme). Quantitative real-time PCR (qRT-PCR) was performed using Taq Pro Universal SYBR qPCR Master Mix (Cat#: Q712-02, Vazyme) on a Bio-Rad CFX96 system.

### Immunofluorescence staining

Gastrocnemius muscle tissues from the limbs of ischemic or sham-operated mice were harvested, embedded in OCT compound and stored at −80 °C. Tissue sections (6 μm thick) were prepared and fixed with ice-cooled acetone for 10 min. Permeabilization was performed using 0.3% Triton X-100 in Tris-buffered saline with Tween 20 (TBST) for 15 min at room temperature. Sections were blocked with a solution containing 1% BSA and 10% donkey serum for 60 min at room temperature to reduce non-specific binding. Sections were then incubated overnight at 4 °C with CD31 Alexa Fluor 647 antibody (1:100 dilution, Santa Cruz). Nuclei were counterstained with DAPI (1 μg/ml, Sigma-Aldrich). Images were acquired using a Leica SP5 II confocal fluorescence microscope (Leica, Germany).

### Histological staining

Mouse gastrocnemius tissue sections were fixed in 4% paraformaldehyde for 10 min. Subsequently, haematoxylin and eosin staining was performed using a commercial kit (Cat#: G1120, Solarbio, Beijing, China) to assess inflammatory-cell infiltration. Fibrosis was detected using a picrosirius red stain kit (Cat#: ab245887, Abcam). Images were captured using a Leica microscope (Germany).

### Terminal deoxynucleotidyl transferase dUTP nick end labelling and dihydroethidium immunofluorescent staining

Apoptosis in gastrocnemius muscle tissue sections was detected using a One-step terminal deoxynucleotidyl transferase dUTP nick end labelling (TUNEL) Cell Apoptosis Detection Kit (Cat#: T2190, Solarbio, Beijing, China) according to the manufacturer’s protocol. Reactive oxygen species (ROS) levels were measured using dihydroethidium (DHE, Beyotime, Nanjing, China) following the manufacturer’s instructions. Nuclei were counterstained using 4',6-diamidino-2-phenylindole (1 μg/ml, Sigma-Aldrich). Images were acquired using a fluorescence microscope (Leica).

### Protein co-immunoprecipitation

Cell pellets were lysed in ice-cold lysis buffer and incubated at 4 °C for 10 min. To remove the cellular debris, the lysate solution was centrifuged at 15,000 *g* for 15 min at 4 °C. The lysate was then divided into two equal aliquots and treated with RNase A and T1 enzymes, followed by incubation at room temperature for 20 min. The supernatant was filtered through a 0.45-μm membrane syringe filter. A portion of the lysate was saved as input, whereas the remaining lysate was incubated with anti-Flag M2 magnetic beads for 4 h at 4 °C. The beads were washed thoroughly and eluted at 4 °C for 2 h. Eluted samples were subjected to western blot analysis, with 2 μg of the IP sample and 10 μg of the input loaded for detection.

### FGF16 RNA immunoprecipitation quantitative PCR

To elucidate the interaction between IGF2BP2 and FGF16 RNA, we isolated total RNA from control shRNA- and IGFBP5 shRNA-infected HUVECs. RNA samples were treated with the IGF2BP2 RIP antibody (Cat#: 03-251, Millipore, Boston, MA, USA) for RNA immunoprecipitation qPCR detection. Reverse transcriptase PCR (RT-PCR) and qRT-PCR were then performed to confirm the interaction between FGF16 RNA and the IGF2BP2 protein. The FGF16 primer sequences were as follows: forward primer, AACGTGCCCTTAGCTGACTC; reverse primer, CTTCAGGTGGGCGAAGTCT.

### FGF16 translational efficiency assay

We constructed the GPL-FGF16 dual luciferase reporter and transfected it into HUVECs infected with control shRNA and IGFBP5 shRNA. The FGF16 translational efficiency was assessed using a Dual-Glo Luciferase Assay System (Cat#: E2920, Promega) 48 h post-transfection, following the manufacturer’s instructions. After 48 h, cells were treated with 75 μl of Dual-Glo Reagent for 2 h. Firefly luminescence was measured using a microplate reader, followed by the addition of 75 μl of Dual-Glo Stop & Glo Reagent and a 10-min incubation. Renilla luminescence was subsequently measured. The ratio of firefly to Renilla luminescence was calculated to determine translational efficiency.

### Probe-based RT-PCR detection of different m^6^A sites in FGF16

The probes for detecting m^6^A sites in FGF16 were synthesized by Aksomics Biotech Inc. (Shanghai, China). For probe-based RT-PCR detection, total RNA was extracted using TRIzol reagent (Life Technology, CA, USA) from HUVECs infected with control-shRNA or IGFBP5-shRNA. A mixture (20 nM probe L, 20 nM probe R, 1× ligation buffer, and 1000 ng treatment RNA) was added to the RNA samples (1000 ng of RNA), incubated for 3 min at 85 °C, and then an additional 10 min at 35 °C. Next, 20 μl of ligation buffer was added to the mixture and incubated at room temperature for 10 min. Real-time PCR was performed using the following protocol: 10 min at 95 °C for holding, 15 s at 95 °C (40 cycles) for denaturation, and 1 min at 60 °C for extension. The methylated and unmethylated sites of the target gene in each sample were subjected to a real-time PCR reaction separately. The primers used for this detection are shown in Supplementary Table 2.

### Western blotting analysis

Following treatment, cells were lysed using a radioimmunoprecipitation assay (RIPA) lysis solution containing proteinase inhibitors. Protein concentrations were determined using a Bicinchoninic Acid (BCA) Protein Assay Kit (Solarbio, Beijing, China). Samples were boiled in 5 × sodium dodecyl sulfate (SDS) sample buffer at 95–100 °C for 5 min. The protein samples (20 μg each) were separated by SDS-PAGE and then transferred to polyvinylidene fluoride (PVDF) membranes (Bio-Rad, Hercules, CA, USA). After blocking with 5% skimmed milk, the membranes were incubated with primary antibodies targeting AKT, phospho-AKT (Ser473), p44/42 MAPK (ERK1/2), phospho-p44/42 MAPK (ERK1/2) (Thr202/Tyr204), METTL3, METTL14, WTAP, ALKBH5, FTO and β-Actin (dilutions ranging from 1:500 to 1:2000) overnight at 4 °C. Following three washes, the membranes were incubated with secondary antibodies conjugated to horseradish peroxidase for 1 h. Detection was performed using an enhanced chemiluminescence reagent (Solarbio, Beijing, China) and a Bio-Rad chemiluminescence imaging system. The grey values were quantified using ImageJ (NIH, USA).

### Statistical analysis

All experiments were performed in a minimum of triplicate, with both in vivo and in vitro studies included. Data analysis and figure preparation were carried out using GraphPad Prism (version 10.0). Statistical differences between the two groups were assessed using an unpaired two-tailed Student’s *t* test, whereas one-way or two-way ANOVA with Tukey’s multiple comparisons test was used for multi-group comparisons. Data are presented as mean ± standard error of the mean. The number of experiments is indicated in the figure legends. A *P* value < 0.05 was considered statistically significant.

## Results

### IGFBP5 deficiency elevates m^6^A levels and upregulates m^6^A writers in human umbilical vein endothelial cells

**Fig. 1:**
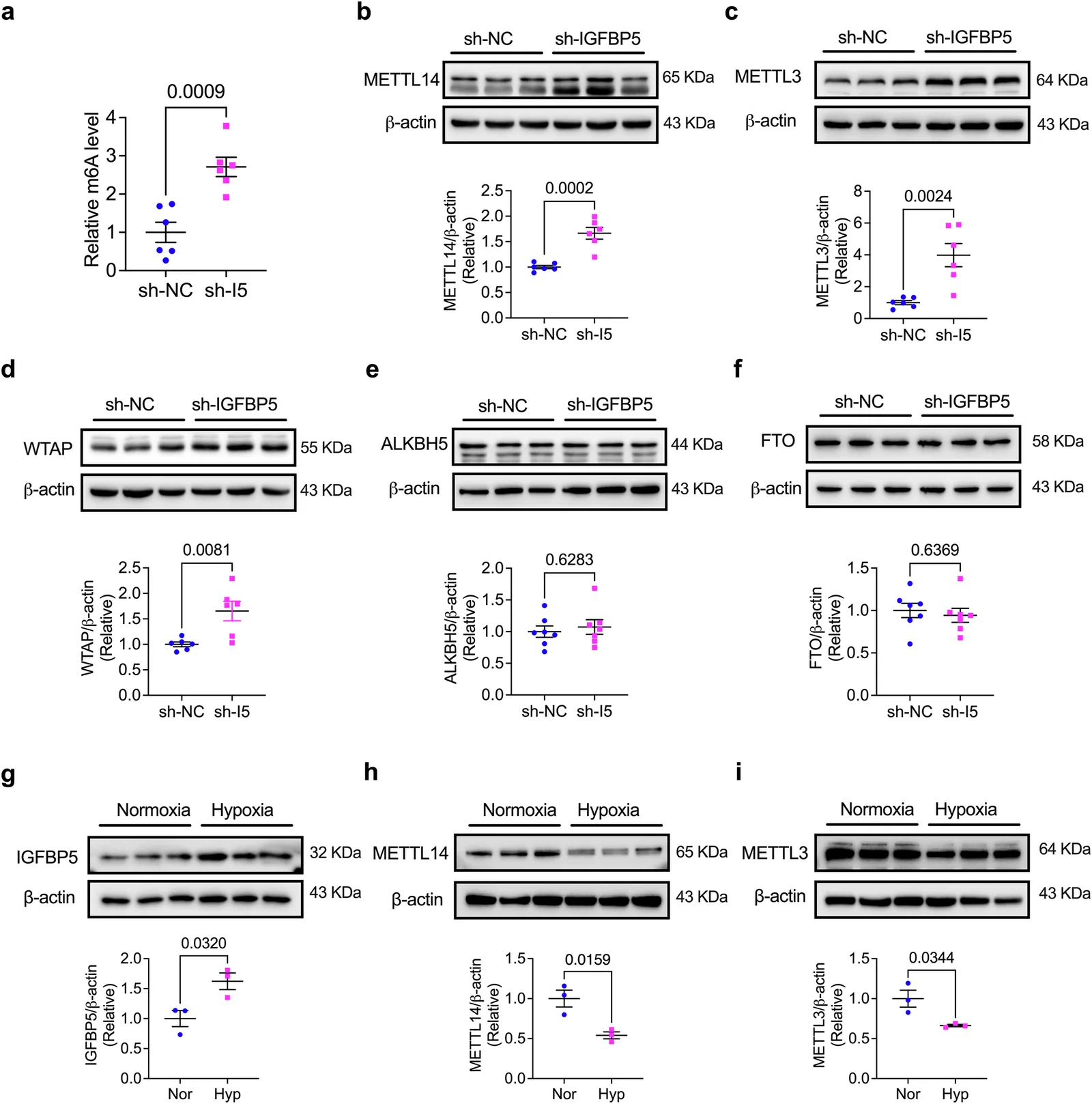
IGFBP5 knockdown elevates global m^6^A level and upregulates methyltransferase expression. **a** Total m^6^A levels in control shRNA- and IGFBP5 shRNA-infected human umbilical vein endothelial cells (HUVECs) measured by m^6^A RNA methylation assay (*n* = 6 per group). **b–d** Representative western blotting images and quantitative analysis of METTL14 (**b**), METTL3 (**c**), and WTAP (**d**) in control shRNA- and IGFBP5 shRNA-infected HUVECs (*n* = 6 per group). **e**, **f**, Representative western blotting images, and quantification of ALKBH5 (**e**) and FTO (**f**) in control shRNA- and IGFBP5 shRNA-infected HUVECs (*n* = 7 per group). **g–i** Representative western blotting images, and quantification of IGFBP5 (**g**), METTL14 (**h**), and METTL3 (**i**) under normoxia or hypoxia conditions in HUVECs (*n* = 3 per group). Data are shown as mean ± SEM. Unpaired, two-tailed Student’s *t* tests were performed for **a–i**.

m^6^A modification plays a significant role in regulating tumour angiogenesis, cellular proliferation and migration. Its regulatory function is mediated by the dynamic balance between methyltransferases (m^6^A writers) and demethylases (m^6^A erasers)^10,23,27–29^. We knocked down IGFBP5 in HUVECs (Supplementary Fig. 1). Using m^6^A-RIP-qPCR, we observed a significant increase in total m^6^A levels in IGFBP5-deficient HUVECs compared with the control group (Fig. 1a). To investigate whether IGFBP5 regulates m^6^A modification, we examined the expression levels of m^6^A writers, including methyltransferase-like 3 (METTL3), METTL14 and WTAP. The data revealed that the expression of m^6^A writers, including METTL14 (Fig. 1b), METTL3 (Fig. 1c), and WTAP (Fig. 1d), was significantly increased in IGFBP5-deficient HUVECs. However, no significant changes were observed in the levels of m^6^A erasers, including ALKBH5 (Fig. 1e) and FTO (Fig. 1f). When the recombinant IGFBP5 was added, these upregulated proteins (METTL3, METTL14 and WTAP) by knockdown of IGFBP5 were suppressed (Supplementary Fig. 2). These findings suggest that IGFBP5 deficiency leads to an increase in the expression of m^6^A writers, which may contribute to elevated m^6^A levels in HUVECs. In addition, we also examined the expression of IGFBP5 and m^6^A methyltransferases, such as METTL14 and METTL3, under hypoxic conditions. The results showed that IGFBP5 was significantly upregulated after hypoxia induction (Fig. 1g), whereas METTL14 and METTL3 were significantly downregulated (Fig. 1h and i). These findings suggested that IGFBP5-regulated m^6^A modification may be involved in ischemia-induced pathological damage.

### IGFBP5 interacts with the MT-A70 domain of METTL14 to regulate m^6^A modification

**Fig. 2:**
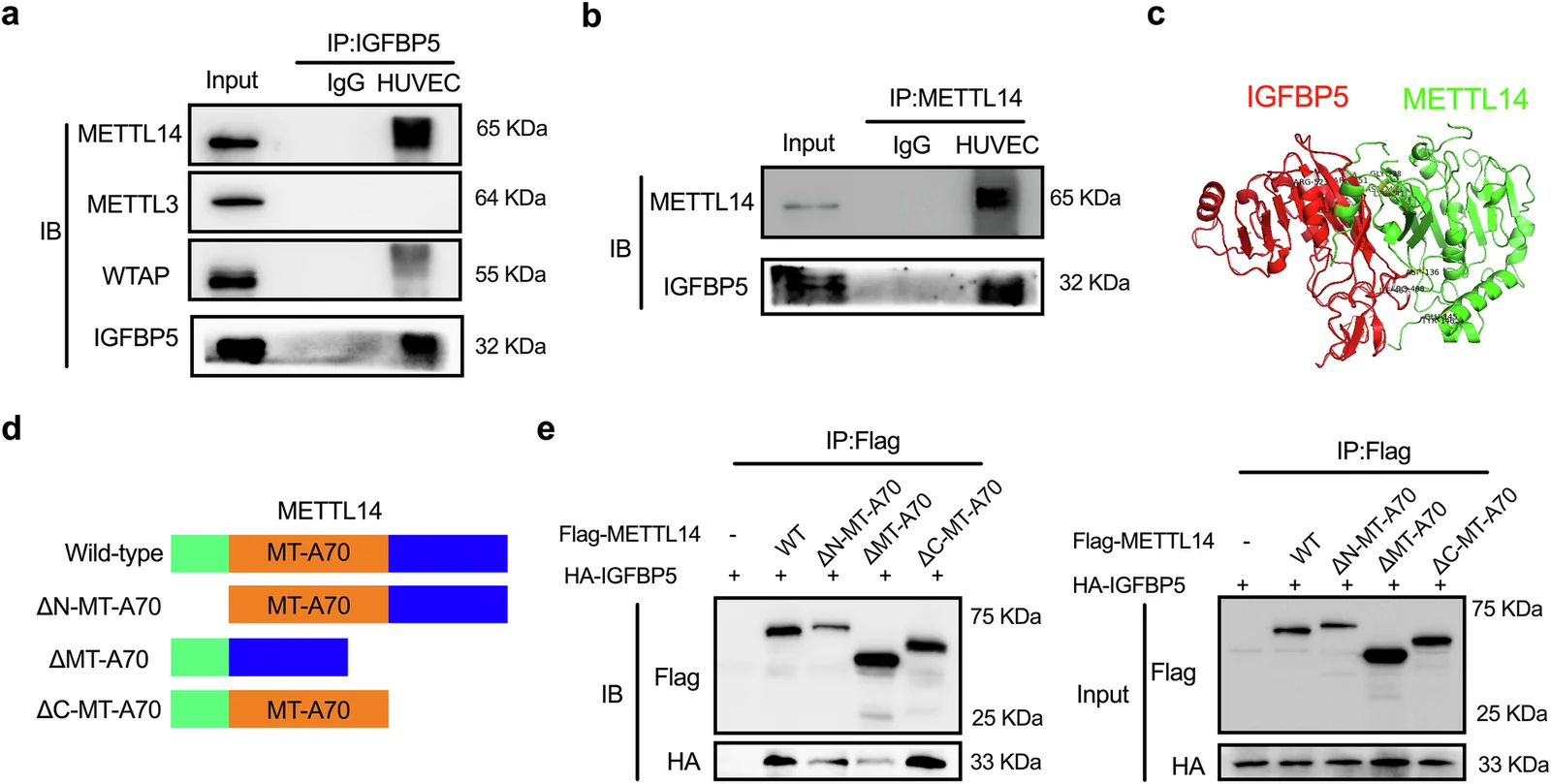
The MT-A70 domain of METTL14 interacts with IGFBP5. **a** Co-immunoprecipitation of IGFBP5 with METTL14, METTL3 and WTAP in human umbilical vein endothelial cells (HUVECs). **b** Reciprocal CO-IP confirming METTL14-IGFBP5 interaction. **c** ZDOCK-predicted structure of the METTL14–IGFBP5 complex (http://zdock.umassmed.edu). **d** Domain architecture of METTL14 constructs: wild type (full-length METTL14, 1–456 amino acids, wild type), Flag-ΔN-MT-A70 (deletion of 1–185 amino acids, N-terminal before MT-A70), Flag-ΔMT-A70 (deletion of 186–363, MT-A70 domain), and Flag-ΔC-MT-A70 (deletion of 364–456 amino acids, C-terminal post MT-A70). **e** CO-IP assay showing interaction between HA-tagged IGFBP5 and Flag-tagged METTL14 truncations (wild type, ΔN-MT-A70, ΔMT-70 and ΔC-MT-A70).

To elucidate the mechanism underlying IGFBP5-mediated m^6^A modification in HUVECs, we performed immunoprecipitation experiments. We found that IGFBP5 specifically binds to METTL14 but not METTL3 or WTAP (Fig. 2a). Reverse immunoprecipitation assays confirmed that METTL14 was the specific protein that bound to IGFBP5 (Fig. 2b). Bioinformatics analysis of the METTL14 protein structure indicated that METTL14 comprises three major structural domains (Fig. 2c). Among these domains, the MT-A70 domain has been previously identified as the core functional component of m^6^A methyltransferase, which is essential for m^6^A modification^30^. To identify the domain of METTL14, which binds IGFBP5, we created four plasmids: wild type Flag-METTL14 (full-length METTL14, 1–456 amino acids, WT), Flag-ΔN-MT-A70 (deletion of 1–185 amino acids, N-terminal before MT-A70), Flag-ΔMT-A70 (deletion of 186–363, MT-A70 domain), and Flag-ΔC-MT-A70 (deletion of 364–456 amino acids, C-terminal post MT-A70) (Fig. 2d). Co-transfection of 293 T cells with the HA-IGFBP5 plasmid showed that the interaction between IGFBP5 and METTL14 was absent in cells lacking the MT-A70 domain of METTL14 (Fig. 2e). These findings demonstrate that IGFBP5 interacts directly with the MT-A70 domain of the METTL14 protein, modulating the formation of methyltransferase complexes and regulating m^6^A modification.

### Knockdown of m^6^A methyltransferases attenuates angiogenesis promoted by IGFBP5 deficiency in human umbilical vein endothelial cells

**Fig. 3:**
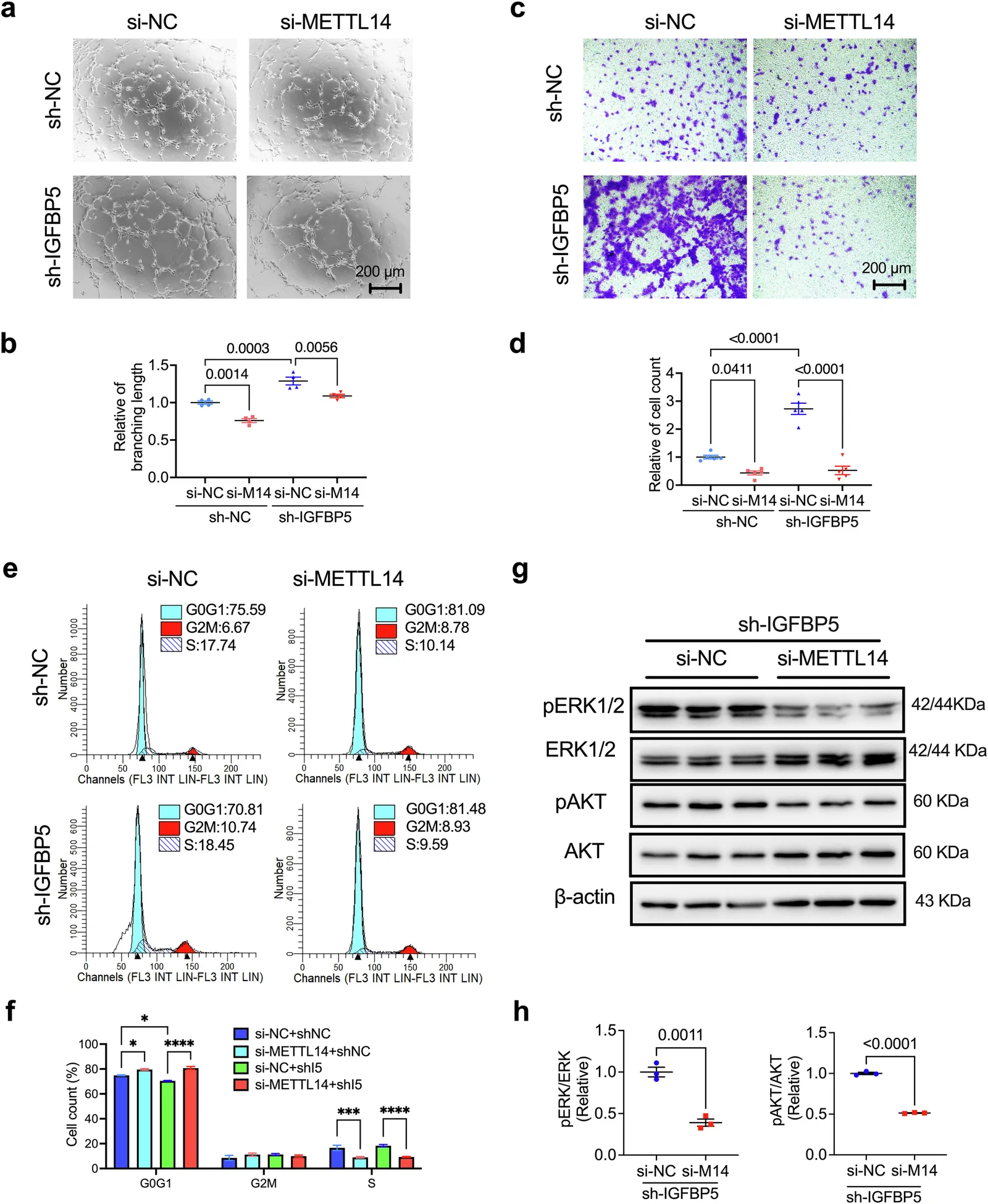
METTL14 knockdown suppresses angiogenic phenotypes in IGFBP5-deficient human umbilical vein endothelial cells. **a** Representative images, and **b** quantification of tube formation in control shRNA- and IGFBP5 shRNA-infected human umbilical vein endothelial cells (HUVECs) transfected with control siRNA (si-NC) and METTL14 siRNA (si-METTL14) (*n* = 4 per group). **c** Representative images, and **d** quantification of Transwell assay in control shRNA- and IGFBP5 shRNA-infected HUVECs transfected with si-NC and si-METTL14 (*n* = 5 per group). **e** Representative images, and **f** quantification of flow cytometry for the cell cycle in control shRNA- and IGFBP5 shRNA-infected HUVECs transfected with si-NC and si-METTL14 (*n* = 5 per group). **g** Western blotting images, and **h** quantification of pERK1/2, ERK1/2, pAKT, and AKT expression in IGFBP5 shRNA-infected HUVECs transfected with si-NC and si-METTL14 (*n* = 3 per group). Data are shown as mean ± SEM. One-way ANOVAs with Tukey’s post hoc tests were performed for **b** and **d**. Two-way ANOVA with Tukey’s multiple comparisons test was performed for **f**. Unpaired, two-tailed Student’s *t* tests were performed for **h**.

Our previous study has shown that IGFBP5 deficiency enhances tube formation, proliferation and migration in HUVECs^9^. To investigate the role of m^6^A methyltransferases in this process, we knocked down METTL3, METTL14 and WTAP using siRNA in IGFBP5-deficient HUVECs (knockdown efficiency of METTL3, METTL14 and WTAP shown in Supplementary Fig. 3). METTL14 silencing markedly reduced tube formation (Fig. 3a and b), suppressed cell migration (Fig. 3c and d), and inhibited cell-cycle progression (Fig. 3e and f) in IGFBP5-deficient cells. Our previous study has demonstrated that IGFBP5 deficiency significantly increases the phosphorylation of AKT and ERK1/2 in HUVECs^9^. In this study, IGFBP5-deficient-increased phosphorylation of ERK1/2 and AKT was decreased upon METTL14 knockdown (Fig. 3g and h and Supplementary Fig. 4). When the recombinant IGFBP5 protein was added, the effect of knocking down METTL14 was neutralized (Supplementary Fig. 5). Similar effects were observed in METTL3- and WTAP-knocked-down HUVECs (Supplementary Figs. 6 and 7). Additionally, we observed that IGFBP5 knockdown-promoted expression of METTL14, METTL3 and WTAP was not suppressed by recombinant IGF1 (Supplementary Fig. 8), suggesting that IGFBP5-regulated m^6^A modification occurs in a non-IGF-dependent manner. Furthermore, we found that recombinant IGFBP5-suppressed tube formation, proliferation and migration were rescued by overexpression of METTL14, which was neutralized by mutation of MT-A70 sites of METTL14 (Supplementary Fig. 9). These findings demonstrate that m^6^A methyltransferases mediate the pro-angiogenic effects of IGFBP5 deficiency.

### Knockdown of METTL14 abolishes the pro-angiogenic effects of IGFBP5 deletion in murine hind limb ischemia model

**Fig. 4:**
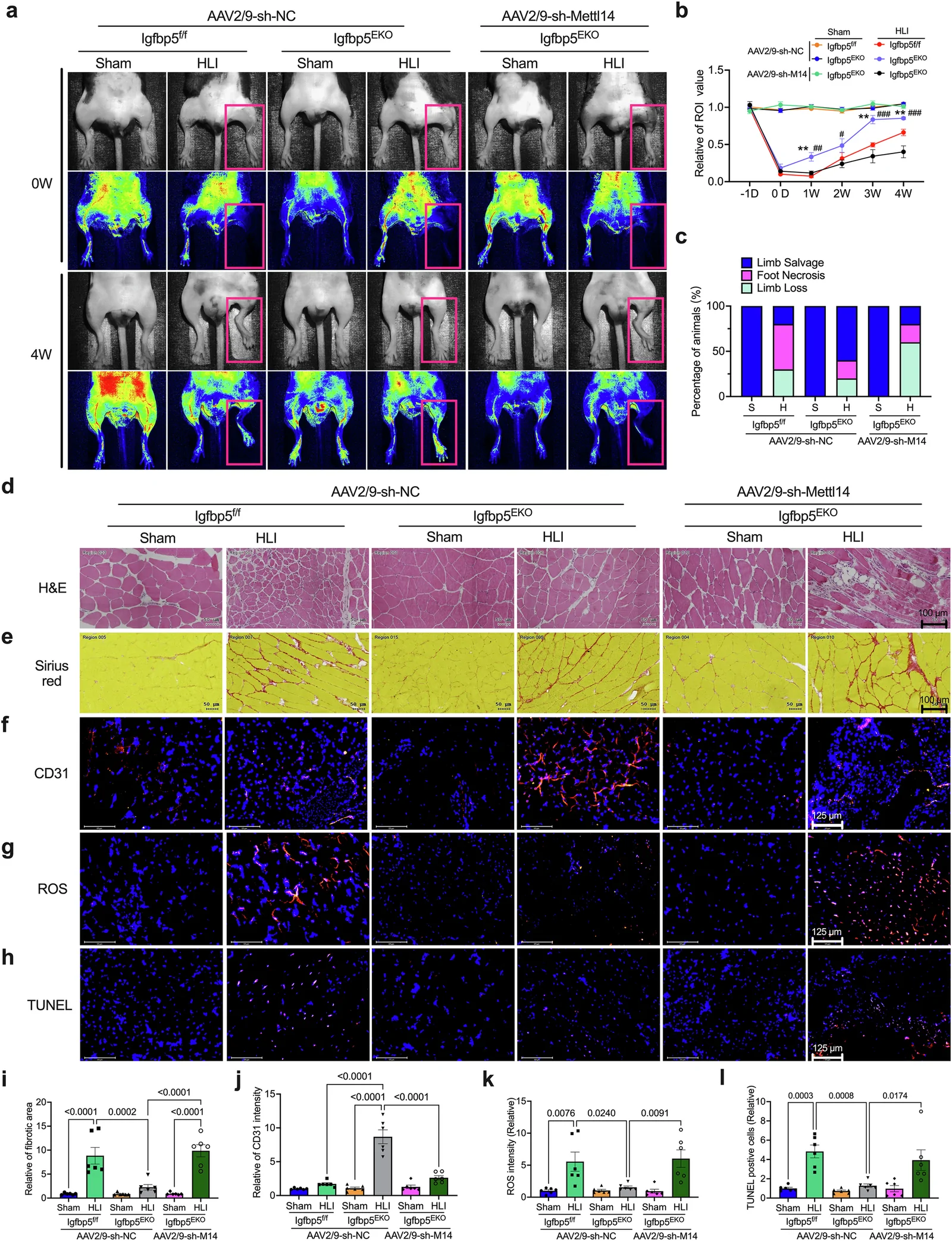
EC-specific METTL14 knockdown reverses the protective effects of IGFBP5 deletion in murine hind-limb ischemia. **a** Laser Doppler-based tissue perfusion system in AAV2/9-control shRNA- and AAV2/9-Mettl14 shRNA-infected Igfbp5^EKO^ and Igfbp5^f/f^ mice at baseline (0 W) and 4 weeks post-ischemia. **b** Quantification of blood flow in the hind limb of AAV2/9-control shRNA- and AAV2/9-Mettl14 shRNA-infected Igfbp5^EKO^ and Igfbp5^f/f^ mice preoperatively (day −1) and on postoperative days 0, 7, 14, 21 and 28 [*n* = 10 in the hind-limb ischemia (HLI) groups and *n* = 9 in the sham groups]. **c** Percentage of limb salvage, foot necrosis and limb loss in AAV2/9-control shRNA- and AAV2/9-Mettl14 shRNA-infected Igfbp5^EKO^ and Igfbp5^f/f^ mice treated with sham or HLI (*n* = 10 in the HLI groups and *n* = 9 in the sham groups). **d** Representative haematoxylin & eosin (H&E) staining images. **e** Picrosirius red staining images. **f** Immunofluorescence staining of CD31 (red). **g** Reactive oxygen species (ROS) (red). **h** Apoptotic cells (red) with terminal deoxynucleotidyl transferase dUTP nick end labelling (TUNEL) staining of gastrocnemius of hindlimb in AAV2/9-control shRNA- and AAV2/9-Mettl14 shRNA-infected sham or ischemic Igfbp5^EKO^ and Igfbp5^f/f^ mice. Quantification of relative area of **i** fibrosis; **j** CD31; **k** ROS; and **l**, apoptotic cells in gastrocnemius of hindlimb of AAV2/9-control shRNA- and AAV2/9-Mettl14 shRNA-infected Igfbp5^EKO^ and Igfbp5^f/f^ mice treated with sham or HLI (*n* = 6 per group). Data are shown as mean ± SEM. Two-way ANOVA with Tukey’s multiple comparisons test was performed for **b**. One-way ANOVAs with Tukey’s post hoc tests were performed for **i–l**. **P < 0.01 vs Igfbp5^f/f^-HLI-AAV2/9-sh-NC, ^#^*P* < 0.05, ^##^*P* < 0.01, ^###^*P* < 0.001 vs Igfbp5^EKO^-HLI-AAV2/9-sh-Mettl14.

As previously reported, endothelial-specific knockout IGFBP5 (Igfbp5^EKO^) protected against HLI^9^. To explore the role of the IGFBP5-METTL14 axis in vivo, AAV2/9-sh-Mettl14 or control AAV2/9-sh-NC was injected into Igfbp5^EKO^ and wild type (Igfbp5^f/f^) mice prior to HLI surgery. The knockout efficiency of METTL14 in hind-limb endothelial cells is presented in Supplementary Fig. 10. Laser Doppler analysis showed significantly improved blood-flow recovery in Igfbp5^EKO^ mice compared with Igfbp5^f/f^ controls, which was abrogated by METTL14 knockdown (Fig. 4a and b). METTL14 silencing also reversed the reduction in foot necrosis and limb loss observed in Igfbp5^EKO^ mice (Fig. 4a and c). Histological analysis revealed attenuated inflammatory infiltration, fibrosis and myofibre disorganization in Igfbp5^EKO^ mice, effects that were abolished by AAV2/9-sh-Mettl14 (Fig. 4d, e, and i). CD31 immunofluorescence indicated decreased neovascularization in METTL14-deficient Igfbp5^EKO^ mice (Fig. 4f and j), accompanied by elevated ROS production (Fig. 4g and k) and apoptosis (Fig. 4h and l) in ischemic muscle. The expression of m6-associated genes (METTL14, METTL3, WTAP, ALKBH5 and FTO) was also examined in vivo. The data found that HLI-suppressed expression of METTL14, METTL3 and WTAP can be promoted by Igfbp5^EKO^ (Supplementary Fig. 11a–d). The expression of m^6^A erasers, such as ALKBH5 and FTO, was not affected by the knockdown of IGFBP5 or the knockdown of METTL14 (Supplementary Fig. 12–13). These results demonstrate that METTL14 knockdown eliminates the pro-angiogenic and tissue-protective effects of endothelial IGFBP5 deficiency in HLI.

### IGFBP5 deficiency promoted m^6^A modification and mRNA levels of FGF16

**Fig. 5:**
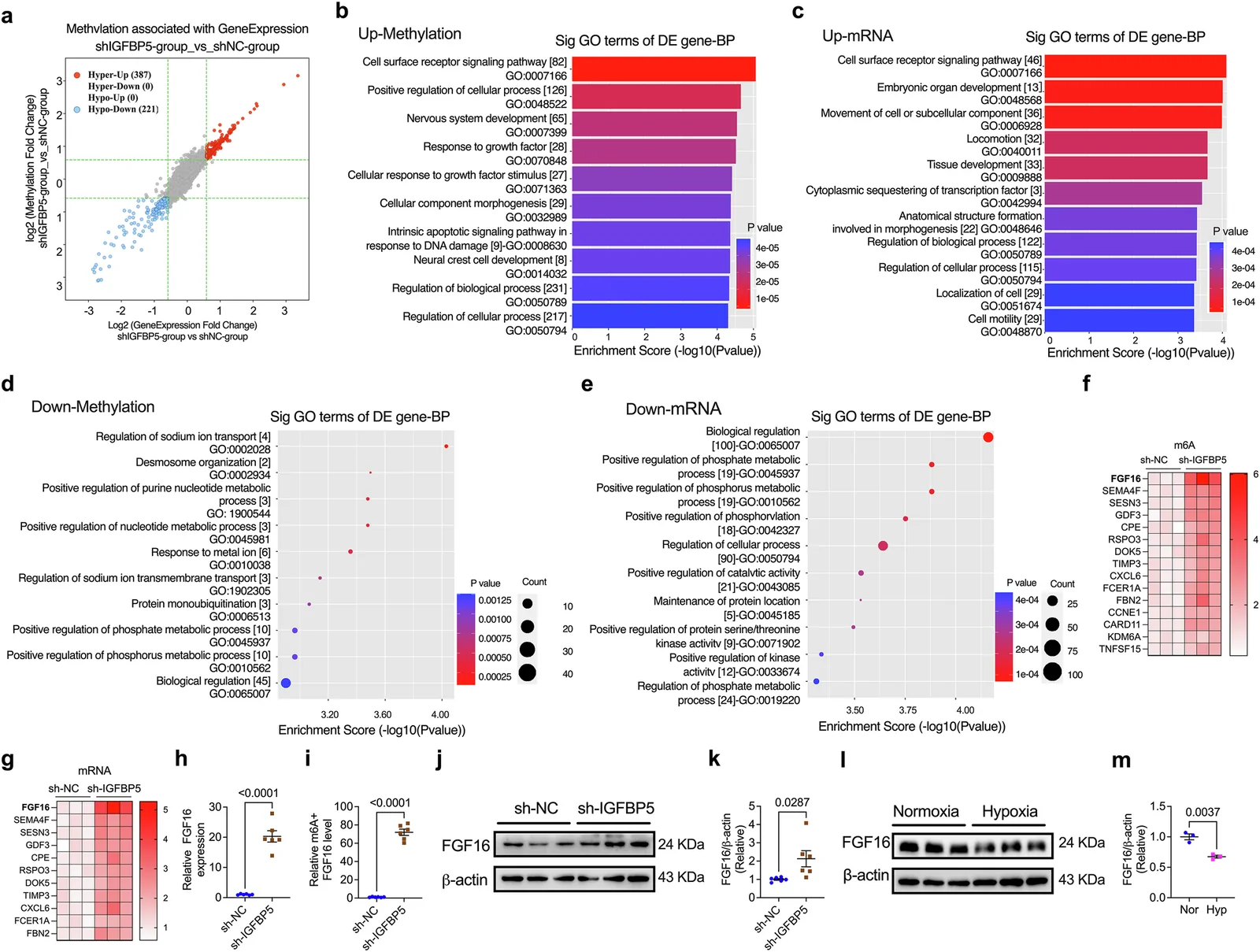
MeRIP-seq reveals m^6^A and transcriptomic changes in control shRNA- and IGFBP5 shRNA-infected human umbilical vein endothelial cells. **a** Different levels of m^6^A and mRNA expression between the IGFBP5 shRNA and control shRNA groups. Dashed lines: *P* < 0.05 (Wilcoxon rank-sum test) and log2 fold change > 1. **b** GO analysis of upregulated m^6^A levels, and **c** upregulated mRNA in IGFBP5 shRNA human umbilical vein endothelial cells (HUVECs) compared with controls. **d** GO analyses of downregulated m^6^A levels, and **e** mRNA expression in IGFBP5 shRNA HUVECs compared with controls. **f** Heatmap of most upregulated mRNA expression, and **g** m^6^A levels in cell-surface receptor signalling pathway in control shRNA- and IGFBP5 shRNA-transfected HUVECs. **h** Relative levels of FGF16 RNA in control shRNA- and IGFBP5 shRNA-transfected HUVECs (*n* = 6 per group). **i** Relative m^6^A levels of FGF16 in control shRNA- and IGFBP5 shRNA-transfected HUVECs (*n* = 6 per group). **j** Western blotting assay, and **k** relative protein levels of FGF16 in control shRNA- and IGFBP5 shRNA-transfected HUVECs (*n* = 6 per group). **l** Representative western blotting images, and **m** quantification of FGF16 under normoxia or hypoxia conditions in HUVECs (*n* = 3 per group). Data are shown as mean ± SEM. Unpaired, two-tailed Student’s *t* tests were performed for **h**, **i**, **k** and **m**.

To investigate the mechanism of the IGFBP5-m^6^A axis in regulating angiogenesis, we performed m^6^A-RIP-seq analysis in HUVECs transfected with control (sh-NC) or IGFBP5 shRNA (sh-IGFBP5). Integrated analysis of mRNA expression and m^6^A profiles revealed 387 genes with coordinated upregulation of both mRNA abundance and m^6^A methylation (Supplementary Table 3), whereas 221 genes showed concurrent downregulation of both parameters (Supplementary Table 4) in IGFBP5-deficient cells (Fig. 5a). Notably, no inverse correlation between mRNA expression and methylation was observed (Fig. 5a). Gene Ontology (GO) enrichment analysis demonstrated that hypermethylated mRNAs were significantly enriched in pathways related to cell-surface receptor signalling, positive regulation of cellular processes, nervous-system development, and growth-factor responsiveness (Fig. 5b; Supplementary Table 5). Upregulated mRNAs were predominantly associated with embryonic organ development and cellular motility (Fig. 5c; Supplementary Table 6). Conversely, hypomethylated mRNAs were linked to ion transport and metabolic processes (Fig. 5d; Supplementary Table 7), whereas downregulated mRNAs clustered around biological regulation and phosphate metabolism (Fig. 5e; Supplementary Table 8).

Strikingly, fibroblast growth factor 16 (FGF16), but not other angiogenesis-related genes, such as RSPO3, TIMP3 and CXCL6 (Supplementary Fig. 14), exhibited the most pronounced increases in both mRNA levels and m^6^A methylation (Fig. 5f and g). FGF16, a 207-amino-acid secreted protein, is critically involved in regulating cell growth, differentiation, proliferation and survival. Reverse transcription quantitative PCR and m^6^A-RIP-qPCR confirmed that IGFBP5 knockdown significantly elevated FGF16 mRNA levels and m^6^A methylation (Fig. 5h and i). Concordantly, FGF16 protein expression also increased post knockdown of IGFBP5 (Fig. 5j and k) and decreased under hypoxic conditions (Fig. 5l and m). Collectively, these data establish FGF16 as a key downstream effector of IGFBP5-mediated m^6^A-dependent regulation.

### IGFBP5 deficiency enhances FGF16 translational efficiency through site-specific m^6^A methylation at position 255

**Fig. 6:**
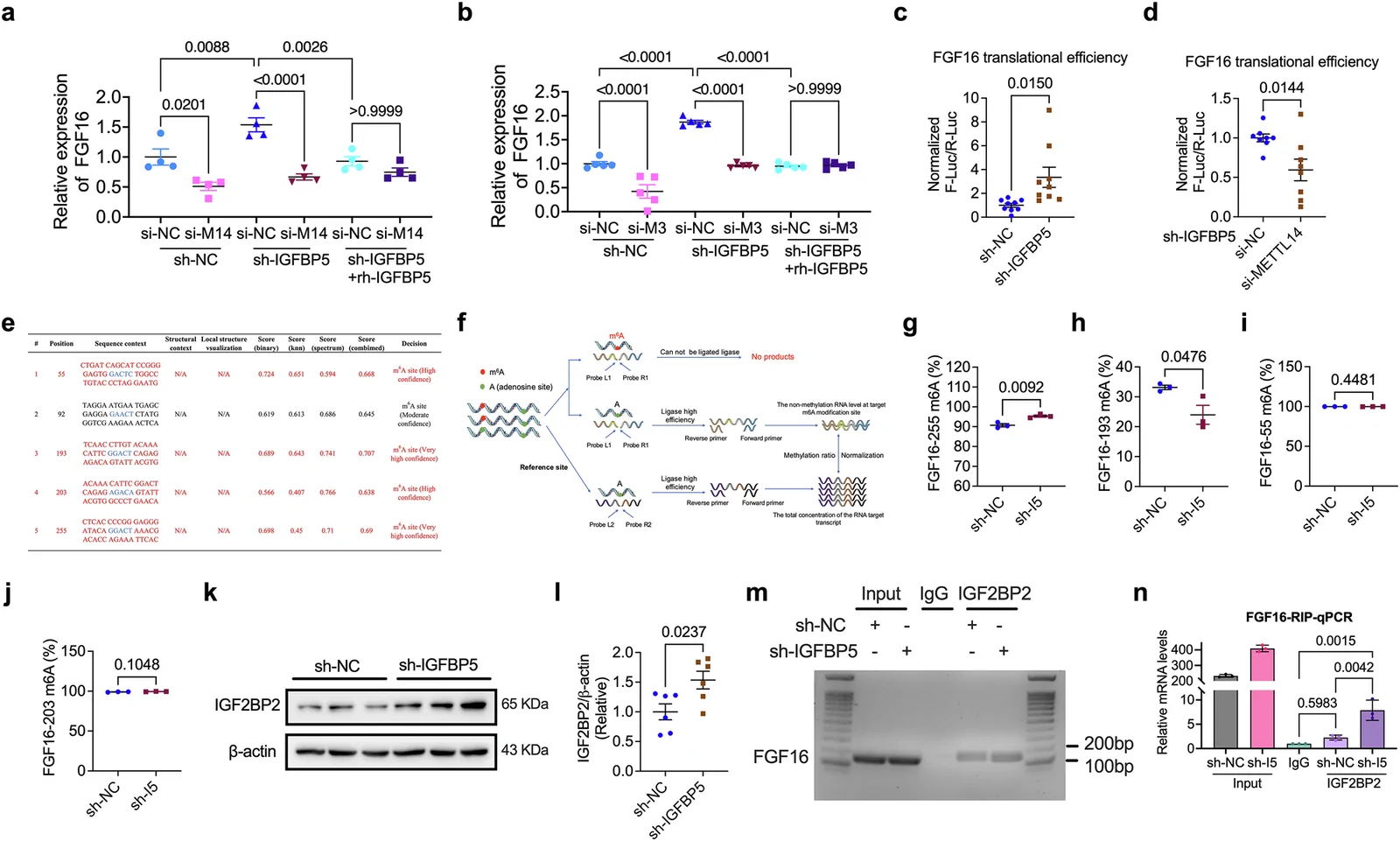
IGFBP5 regulates FGF16 m^6^A modification via IGF2BP2. **a** mRNA level of FGF16 in control siRNA- and METTL14 siRNA-transfected human umbilical vein endothelial cells (HUVECs) infected with control shRNA and IGFBP5 shRNA and treated with recombinant IGFBP5 detected by quantitative real-time PCR (qRT-PCR) (*n* = 4 per group). **b** mRNA level of FGF16 in control siRNA- and METTL3 siRNA-transfected HUVECs infected with control shRNA and IGFBP5 shRNA and treated with recombinant IGFBP5 detected by qRT-PCR (*n* = 6 per group). **c** FGF16 translational efficiency in control shRNA- and IGFBP5 shRNA-transfected HUVECs (*n* = 9 per group). **d** FGF16 translation efficiency in control siRNA- and METTL14 siRNA-transfected IGFBP5-deficient HUVECs (*n* = 8 per group). **e** m^6^A site prediction of FGF16 using the SRAMP platform. **f** Construction process of targeting m^6^A sites of FGF16. **g–j** A probe-based test of m^6^A levels in different m^6^A sites of FGF16 (**g**, 255 sites; **h**, 193; **i**, 55 sites; and **j**, 203) in control shRNA- and IGFBP5 shRNA-infected HUVECs (*n* = 3 per group). **k** Western blotting images, and **l**, quantification of IGF2BP2 in sh-NC- and sh-IGFBP5-infected HUVECs (*n* = 6 per group). **m**, mRNA levels of FGF16 in the IGF2BP2 RNA immunoprecipitation tested with PCR, and **n**, qRT-PCR analysis (*n* = 3 per group). Data are shown as mean ± SEM. An unpaired, two-tailed Student’s *t* test was performed for **c**, **d**, **g**, **h**, **i**, **j**, and **l**. One-way ANOVAs with Tukey’s post hoc test was performed for **a**, **b** and **n**.

To delineate the IGFBP5-m^6^A axis in regulating FGF16 expression, we knocked down METTL14 or METTL3 in IGFBP5-deficient HUVECs and quantified FGF16 levels. Both METTL14 and METTL3 silencing significantly attenuated IGFBP5 deficiency-induced FGF16 upregulation, which can be neutralized by rhIGFBP5 (Fig. 6a and b). Dual-luciferase reporter assays revealed that IGFBP5 deficiency enhanced FGF16 translation efficiency, which was reversed by METTL14 knockdown (Fig. 6c and d). The expression of FGF16 was also examined in normal and ischemic hind-limb tissues of wild type and Igfbp5^EKO^ mice, with or without Mettl14 shRNA AAV injection. We find that FGF16 was significantly promoted by knockout of IGFBP5, which was suppressed by knockdown of METTL14 (Supplementary Fig. 15). These findings highlight the critical role of m^6^A modification in IGFBP5-mediated FGF16.

Bioinformatic prediction using SRAMP (http://www.cuilab.cn/sramp) identified four high-confidence m^6^A sites in FGF16 mRNA (positions 55, 193, 203 and 255) (Fig. 6e). Targeting probes were designed for these sites (Fig. 6f). Site-specific m^6^A quantification showed that position 255 exhibited >90% m^6^A occupancy, with a significant increase in IGFBP5-deficient cells (Fig. 6g). In contrast, position 193 showed reduced methylation, whereas positions 55 and 203 remained unchanged (Fig. 6h–j). Collectively, these data establish the 255 m^6^A site as the primary locus mediating IGFBP5-dependent regulation of FGF16.

The m^6^A modification is specifically recognized by m^6^A-binding proteins called m^6^A readers. Our further finding showed that the m^6^A reader IGF2BP2 was upregulated in IGFBP5-deficient cells (Fig. 6k and l, Supplementary Fig. 16a), but not other m^6^A readers (Supplementary Fig. 16b–d). Insulin-like growth factor 2 mRNA-binding protein (IGF2BP2) is an RNA-binding protein that plays a crucial role in post-transcriptional regulation of mRNA localization, stability and translational control, or as an m^6^A reader that targets mRNA transcripts and promotes the stability of mRNAs^31^. RIP-PCR and RIP-qPCR showed that FGF16 mRNA binds to IGF2BP2 protein and enhanced binding of them upon IGFBP5 knockdown (Fig. 6m and n), indicating that IGFBP5 deficiency promotes FGF16 stability via IGF2BP2-mediated m^6^A recognition.

### Knockdown of FGF16 antagonized the pro-angiogenic effects of IGFBP5 deficiency

**Fig. 7:**
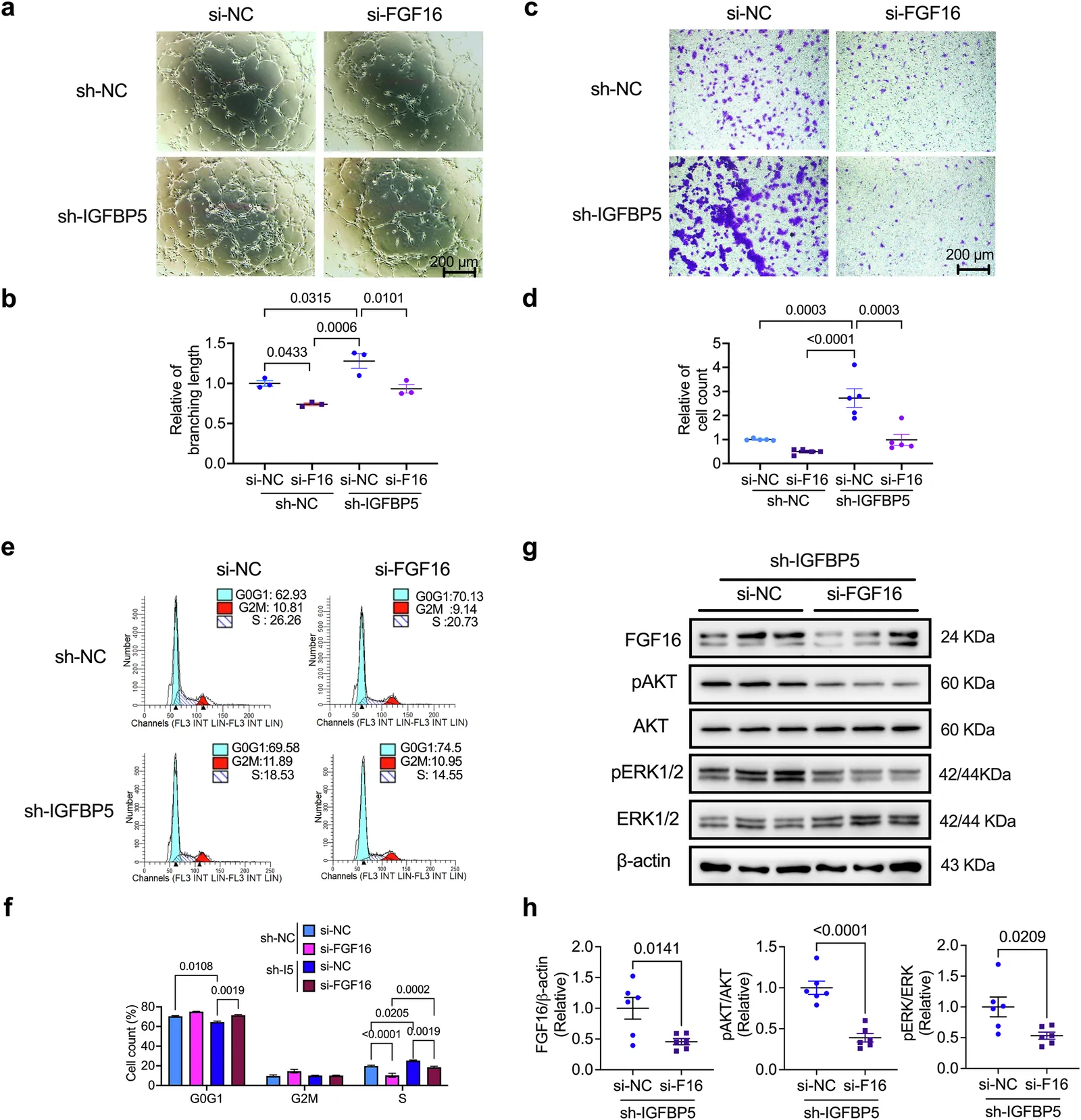
FGF16 knockdown attenuates IGFBP5 deficiency-induced angiogenesis in human umbilical vein endothelial cells. **a** Representative images, and **b** quantification of tube formation in control shRNA- and IGFBP5 shRNA-infected human umbilical vein endothelial cells (HUVECs) transfected with control siRNA (si-NC) or FGF16 siRNA (si-FGF16) (*n* = 3 per group). **c** Representative images, and **d** quantification of Transwell assay in control shRNA- and IGFBP5 shRNA-infected HUVECs transfected with si-NC and si-FGF16 (*n* = 5 per group). **e** Representative images, and **f** quantification of flow cytometry for the cell cycle in control shRNA- and IGFBP5 shRNA-infected HUVECs transfected with si-NC and si-FGF16 (*n* = 5 per group). **g** Representative western blotting images, and **h** quantification of pERK1/2, ERK1/2, pAKT and AKT expression in IGFBP5 shRNA-infected HUVECs transfected with si-NC and si-FGF16 (*n* = 6 per group). Data are shown as mean ± SEM. One-way analyses of variance (ANOVAs) with Tukey’s post hoc tests were performed for **b** and **d**. Two-way ANOVA with Tukey’s multiple comparisons test was performed for **f**. An unpaired, two-tailed Student’s *t* test was performed for **h**.

To investigate the role of FGF16 in IGFBP5-regulated angiogenesis, we silenced FGF16 using siRNA in IGFBP5-deficient HUVECs. The knockdown efficiency of FGF16 was shown in Supplementary Fig. 17. FGF16 knockdown significantly attenuated the pro-angiogenic effects of IGFBP5 deficiency, including reduced tube formation capacity and shorter branch lengths in Matrigel assays (Fig. 7a and b). FGF16 silencing also suppressed the enhanced migratory capacity of IGFBP5-deficient HUVECs in Transwell assays (Fig. 7c and d). Furthermore, FGF16 knockdown reversed the pro-proliferative effect of IGFBP5 deficiency, as demonstrated by cell-cycle analysis (Fig. 7e and f). Western blotting revealed that FGF16 silencing attenuated IGFBP5 deficiency-induced phosphorylation of ERK1/2 and AKT, with reduced p-ERK1/2/ERK1/2 and p-AKT/AKT ratios (Fig. 7g and h, Supplementary Fig. 18). Knockdown of FGF16-suppressed tube formation, proliferation and migration was neutralized by rhIGFBP5 (Supplementary Fig. 19). Intriguingly, knockdown IGFBP5-promoted tube formation was suppressed by METTL14 or IGF2BP2 siRNA and rescued by overexpression of FGF16 (Supplementary Fig. 20). Collectively, these data demonstrate that FGF16 is essential for mediating the pro-angiogenic effects of IGFBP5 deficiency and regulating endothelial-cell proliferation, migration and tube formation.

## Discussion

Our previous study demonstrated that IGFBP5 ablation enhanced angiogenesis by promoting ATP metabolism and stabilizing HIF1α through phosphorylation of the IGF1R/AKT/ERK1/2 pathways^9^. In the current study, we identify a novel mechanism through which IGFBP5 deficiency regulates angiogenesis via m^6^A RNA modification: 1) IGFBP5 knockdown upregulated m^6^A methyltransferases (METTL3, METTL14, and WTAP) and global m^6^A levels in HUVECs; 2) IGFBP5 interacts with the MT-A70 domain of METTL14; 3) silencing METTL14, METTL3 or WTAP abolished the IGFPB5 deficiency-driven effect, including enhanced tube formation, accelerated cell-cycle progression and increased migration, by suppressing AKT and ERK1/2 phosphorylation; 4) in vivo endothelial knockdown of METTL14 in Igfbp5^EKO^ mice abrogated the rescue of hind-limb ischemia damage observed in Igfbp5^EKO^ controls; 5) IGFBP5 deficiency enhanced FGF16 binding to the m^6^A reader IGF2BP2, increasing m^6^A levels and the translational efficiency of FGF16 by targeting the 255 m^6^A site.

CLI is a severe stage of PAD that reduces the patient’s quality of life^32^. It is imperative to establish therapeutic strategies to prevent CLI progression^33^. Augmentation of angiogenesis by administration of proangiogenic cytokines or by transplantation of stem/progenitor cells represents a potential approach for treating severe ischemic diseases^34,35^. For instance, implantation of autologous bone-marrow mononuclear cells has significantly improved ischemic symptoms and angiogenesis in patients with CLI^36^. In our previous work, we demonstrated that IGFBP5 ablation enhances angiogenesis by promoting ATP metabolism and stabilizing HIF1α via the IGF1R/AKT/ERK1/2 pathways^9^. However, IGFBP5 also exerts biological functions through non-IGF-dependent mechanisms. In the present study, we found that knockdown of IGFBP5 in HUVECs promoted the expression of m^6^A methyltransferases METTL3, METTL14 and WTAP, as well as the m^6^A levels, indicating that m^6^A methylation may play a critical role in IGFBP5-regulated angiogenesis. Further results demonstrated that knockdown of METTL3, METTL14 or WTAP mitigated the angiogenic effects of IGFBP5 deficiency.

The m^6^A modification is catalysed by the m^6^A methyltransferase (writer) complex, which comprises the METTL3/METTL14 heterodimeric catalytic core and the regulatory subunit WTAP^17,24,25^. METTL3 contains the active methyltransferase domain that catalyses the conversion of adenosine (A) to m^6^A, whereas METTL14 functions as an essential component that supports METTL3 in recognizing RNA substrates. Together, the METTL3–METTL14 heterodimer is crucial for methylation activity^17,22,37^. Our findings revealed that IGFBP5 interacted with the MT-A70 domain of METTL14 and then affected the heterodimer of METTL3–METTL14. Additionally, our data suggested that IGFBP5 specifically interacted with METTL14 under normal conditions, and that this interaction was diminished in the absence of IGFBP5, resulting in a shift in equilibrium. In recent years, there have been major advances in knowledge of the biochemistry and molecular regulation of the m^6^A RNA epitranscriptome in diverse physiological processes. However, studies on the regulatory role of m^6^A in both physiological and pathological angiogenesis, blood-vessel formation, remain limited^38^. Yao et al. found that m^6^A levels were elevated in the transcriptome of ECs and mouse retinas following hypoxic stress, and depletion of METTL3 impaired endothelial-cell angiogenic ability, including viability, proliferation, migration and tube formation in vitro^26^. Another study demonstrated that endothelial expression of METTL3 methylates Notch1 mRNA near the stop codon, leading to suppression of Notch activity in a YTHDF2-dependent manner during endothelial-to-haematopoietic transition^39^. Our results also showed that knockdown of METTL14, METTL3 and WTAP in IGFBP5-deficient ECs inhibited IGFBP5-promoted tube formation, cell-cycle progression and cell migration by downregulating the phosphorylation of AKT and ERK1/2. Furthermore, in vivo knockdown of endothelial METTL14 in IGFBP5 endothelial knockdown (Igfbp5^EKO^) mice reversed Igfbp5^EKO^-rescued blood-flow recovery after hind-limb ischemia.

FGF16 was originally identified in the heart through a homology-based PCR^40^ and is efficiently secreted by an uncleaved bipartite signal sequence^41^. FGF16 is a member of the FGF9 subfamily (comprising FGF9, FGF16 and FGF20), which shares an amino-acid sequence similar to other FGFs, although their similarity to one another varies^40^. In fetal mouse hearts, FGF16 is expressed in the endocardium and epicardium at embryonic days E10.5 and E12.5 in a unique but overlapping pattern with FGF9 and FGF20 (ref.^42^). FGF16 has been implicated in the FGF signalling network, which contributes to the expansion of anterior heart field progenitor cells^43^. FGF16 can induce the migration of primary human endothelial cells in culture^44^. In our present study, we found that depletion of IGFBP5 increased FGF16 expression by enhancing its m^6^A levels and translational efficiency via binding to the m^6^A reader IGF2BP2. Additionally, the knockdown of FGF16 mitigates the IGFBP5 deletion-promoted tube formation, cell proliferation and migration. However, this study has limitations. First, the therapeutic potential of blocking IGFBP5 in large animal models with various ischemic insults needs to be verified. Second, the current study did not investigate the impact of mutation at the 255 m^6^A site of FGF16 on Igfbp5^EKO^-rescued HLI damage.

In summary, our findings demonstrated a novel mechanism by which IGFBP5 critically regulates angiogenesis through the post-transcriptional regulation of FGF16 in an m^6^A-dependent manner. This suggests that targeting the IGFBP5/METTL14/FGF16 axis may represent a therapeutic approach to treating ischemic diseases.

## Supplementary information
