# Supplementary material for: METTL14-dependent N6-methyladenosine modification of FGF16 participates in IGFBP5 deficiency-enhanced angiogenesis
Source: Exp Mol Med. 2026 Aug 4;58(8):2630–42. doi: 10.1038/s12276-026-01788-y (PMC13538678; doi:10.1038/s12276-026-01788-y)
Supplement: Supplementary file 1 [file 12276_2026_1788_MOESM1_ESM.pdf]

Supplementary materials

Reagents and Antibodies

The Hematoxylin-Eosin (H&E) staining kit (Cat#: G1120) and TUNEL apoptosis assay kit (Cat#: T2190) were purchased from Solarbio Life Science (Beijing, China). The Picro Sirius Red stain kit (Cat#: ab245887) was purchased from Abcam (Cambridge, UK). The Tali™ cell cycle kit (Cat#: A10798), Lipofectamine™ RNAiMAX (Cat#: 13778150), Accutase-Enzyme cell detachment medium (Cat#: 00-4555-56), and Lipofectamine™ 3000 (Cat#: L3000008) were purchased from Thermo Fisher Scientific (Waltham, MA, USA). Endothelial Cell Medium (ECM, Cat#: 1001) was purchased from ScienCell Research Laboratories (Carlsbad, CA, USA). Control siRNA (Cat#: sc-37007), METTL3 siRNA (Cat#: sc-92172), METTL14 siRNA (Cat#: sc-89054), WTAP siRNA (Cat#: sc-64224), FGF16 siRNA (Cat#: sc-39474), control shRNA plasmid (Cat#: sc-108060), and IGFBP5 shRNA (h) lentiviral (Cat#: sc-39591-V) were purchased from Santa Cruz Biotechnology (Dallas, Texas, USA). The HB-AAV2/9-Mettl14-TIE-m-ZsGreen and HB-AAV2/9-TIE-ZsGreen controls were ordered from HANBIO (Shanghai, China). Dihydroethidium (DHE, Cat#: S0063) was purchased from Beyotime (Shanghai, China). The Matrigel™ reduced growth factor matrix (Cat#: 356230) was obtained from Corning Life Sciences (Corning, NY, USA). The EpiQuic m6A RNA Methylation Quantification Kit (Cat#: P-9005) was bought from EpigenTek (Farmingdale, NY, USA).

The following antibodies were purchased from Cell Signaling Technology (Boston, MA, USA): anti-IGFBP5 (Cat#: 10941S), anti-Phospho-p44/42 MAPK (ERK1/2) (Thr202/Tyr204) (Cat#: 4370), anti-p44/42 MAPK (ERK1/2) (Cat#: 4695S), anti-Phospho-AKT (Ser473) (Cat#: 4060S), and anti-AKT (Cat#: 9272S). The following antibodies were purchased from Abcam

(Cambridge, UK): anti-METTL3 (Cat#: ab195352), anti-FTO (Cat#: ab126605), anti-ALKBH5 (Cat#: ab195377), anti-DDDK tag (binds to FLAG tag sequence) (Cat#: ab205606), and anti-HA tag. The FGF16 antibody (Cat#: DF8945) was obtained from Affinity Biosciences (Nanjing, China), and anti-β-Actin (Cat#: AC038) was purchased from ABclonal (Wuhan, China) as the loading control.

Supplementary Figures

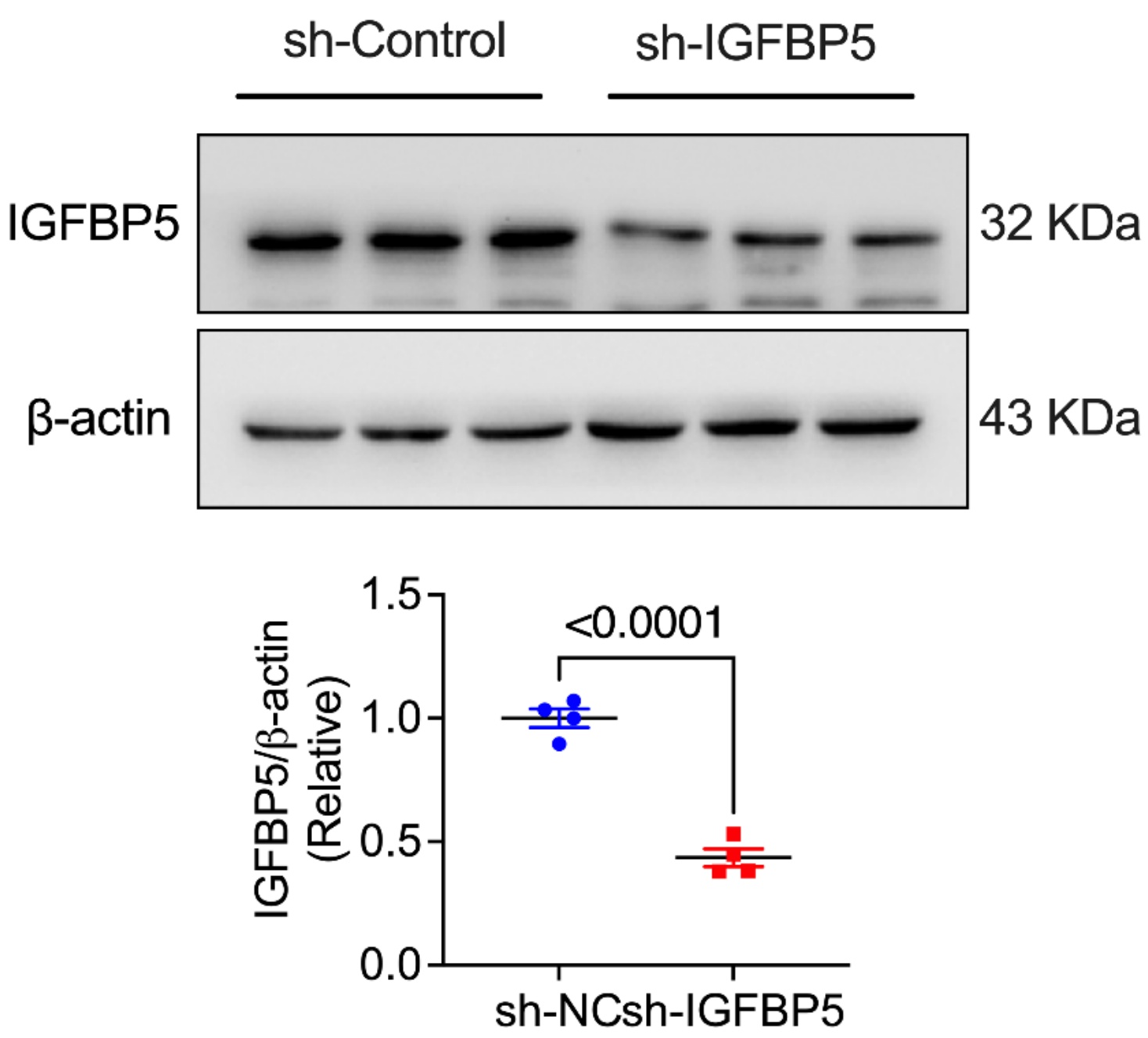

Supplementary Fig. 1. Knockdown efficiency of IGFBP5. The western blotting assay determined the protein levels of IGFBP5 in the sh-control- or sh-IGFBP5-transfected HUVECs.

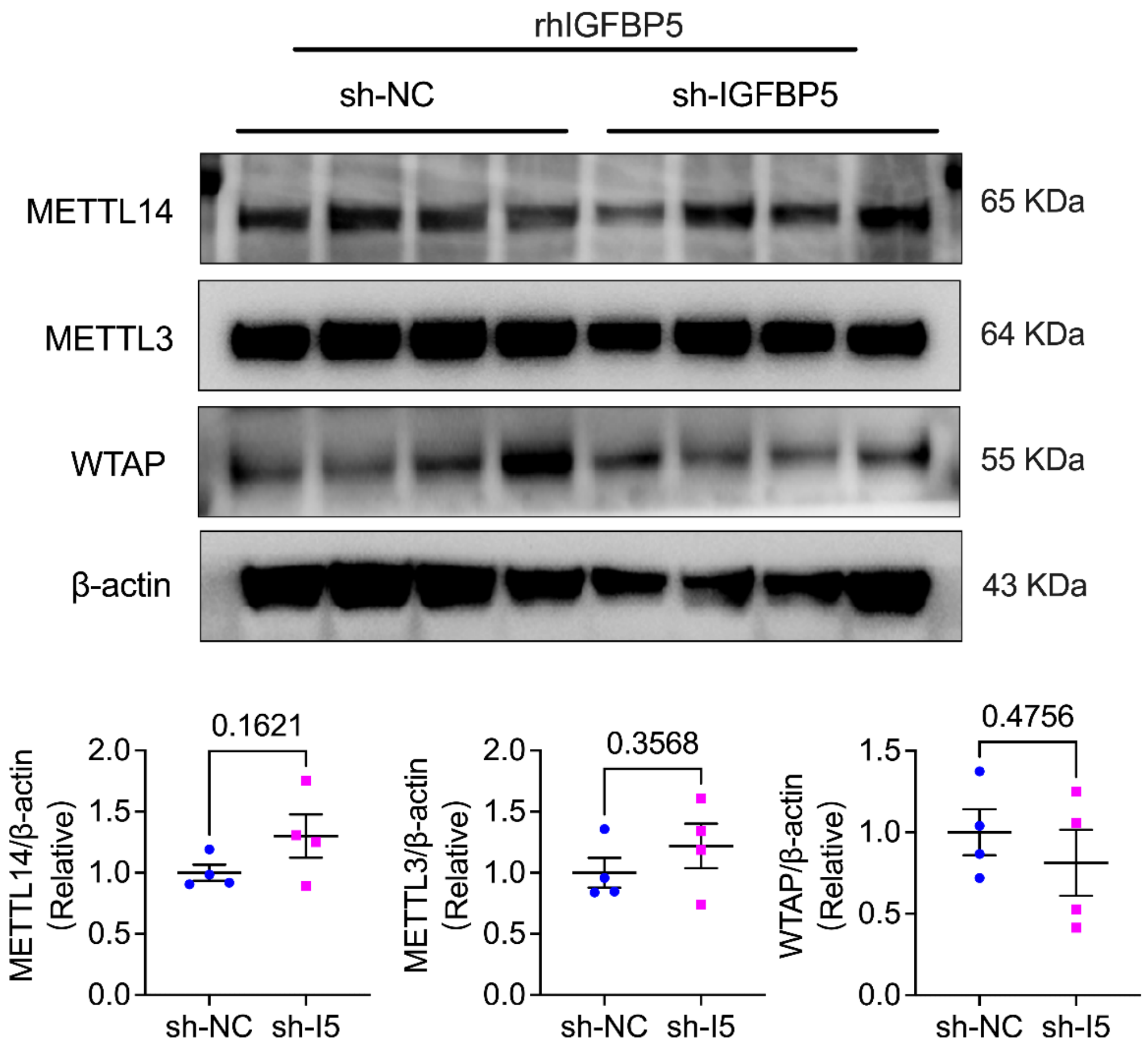

Supplementary Fig. 2. IGFBP5 knockdown-promoted expression of METTL14, METTL3, and WTAP was neutralized by rhIGFBP5. Western blotting images and quantification of METTL14, METTL3, and WTAP in HUVECs transfected with sh-control or sh-IGFBP5 (n = 3 per group).

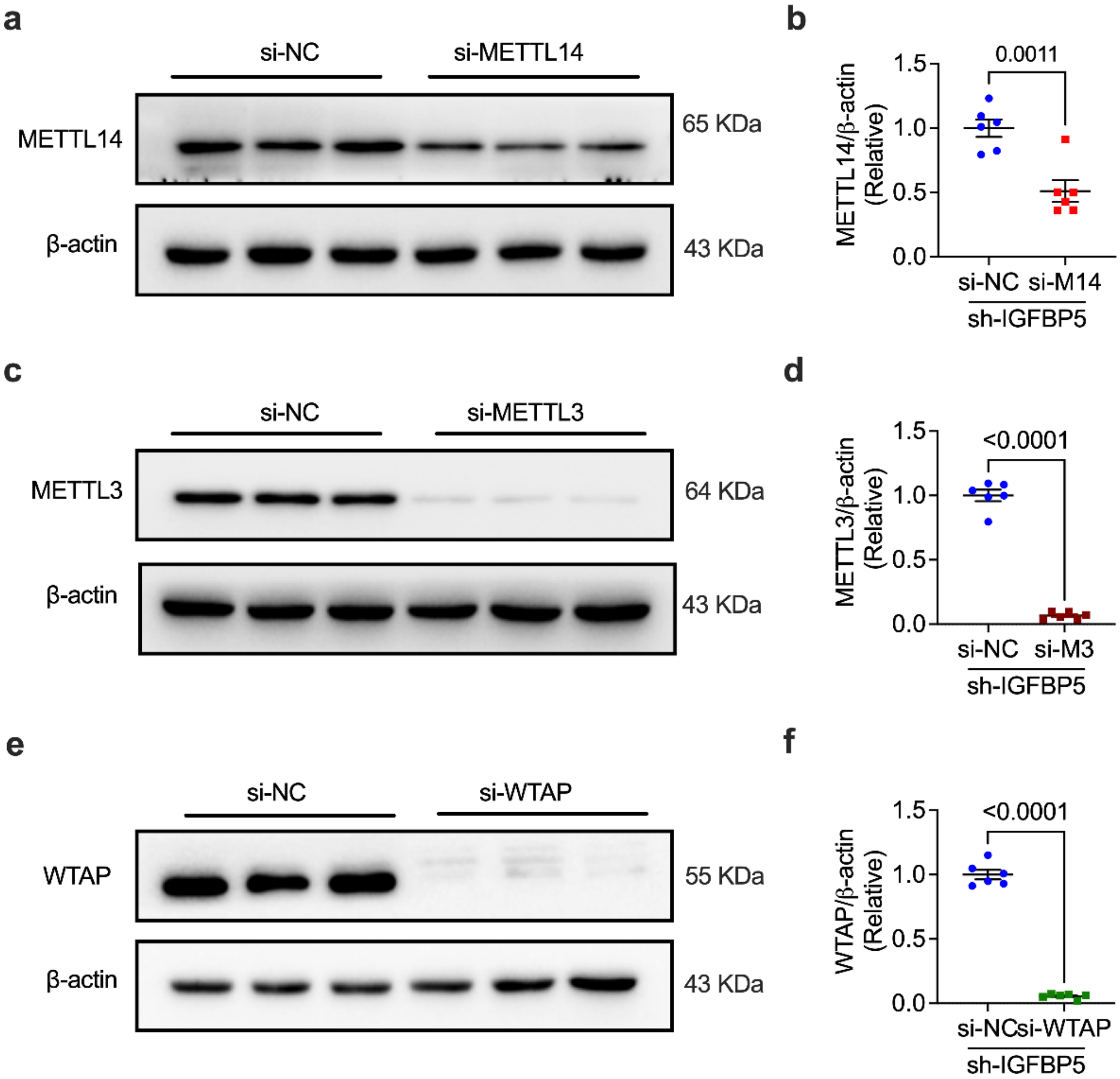

Supplementary Fig. 3. Knockdown efficiency of METTL14, METTL3, and WTAP. a, western blotting images, and **b,** quantification of METTL14 expression in control siRNA- and METTL14 siRNA-transfected HUVECs (n = 6 per group). **c,** western blotting images, and **d,** quantification of METTL3 expression in control siRNA- and METTL3 siRNA-transfected HUVECs (n = 6 per group). **e,** western blotting images, and **f,** quantification of WTAP expression in control siRNA- and WTAP siRNA-transfected HUVECs (n = 6 per group).

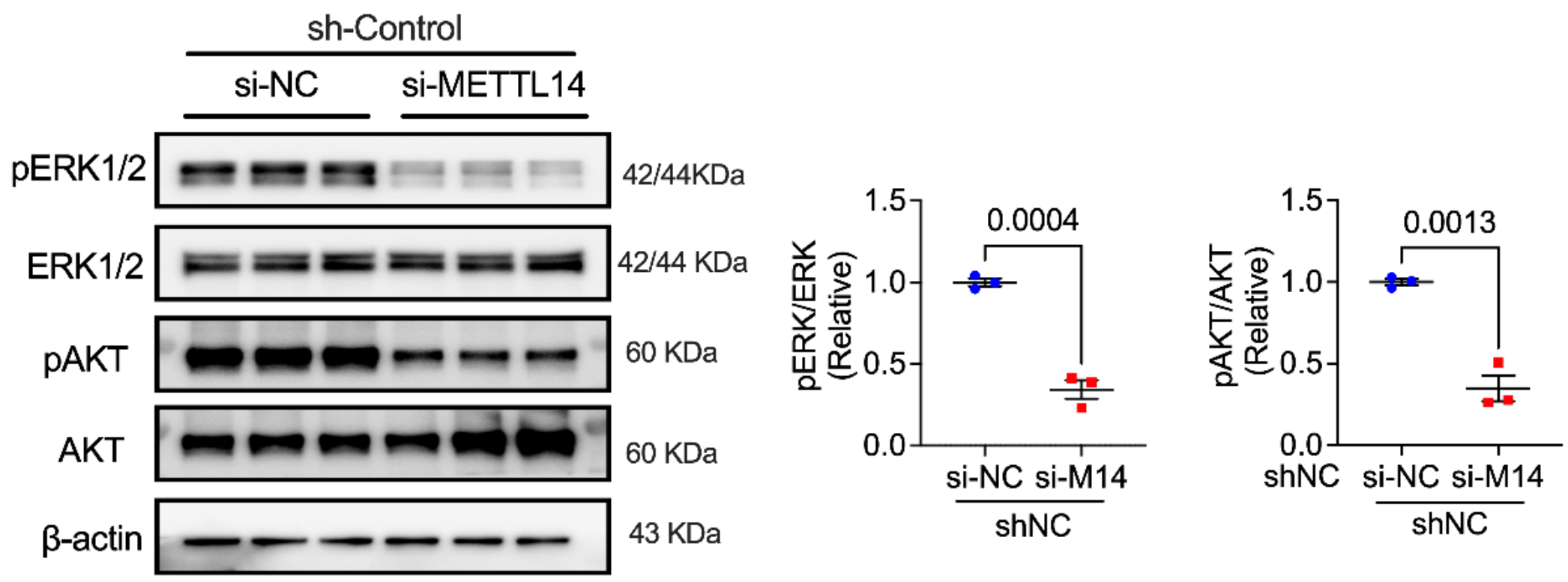

Supplementary Fig. 4. METTL14 knockdown suppressed phosphorylation of ERK1/2 and AKT. Western blotting images and quantification of phosphorylation of ERK1/2 and AKT in HUVECs transfected with si-control or sh-METTL14 (n = 3 per group).

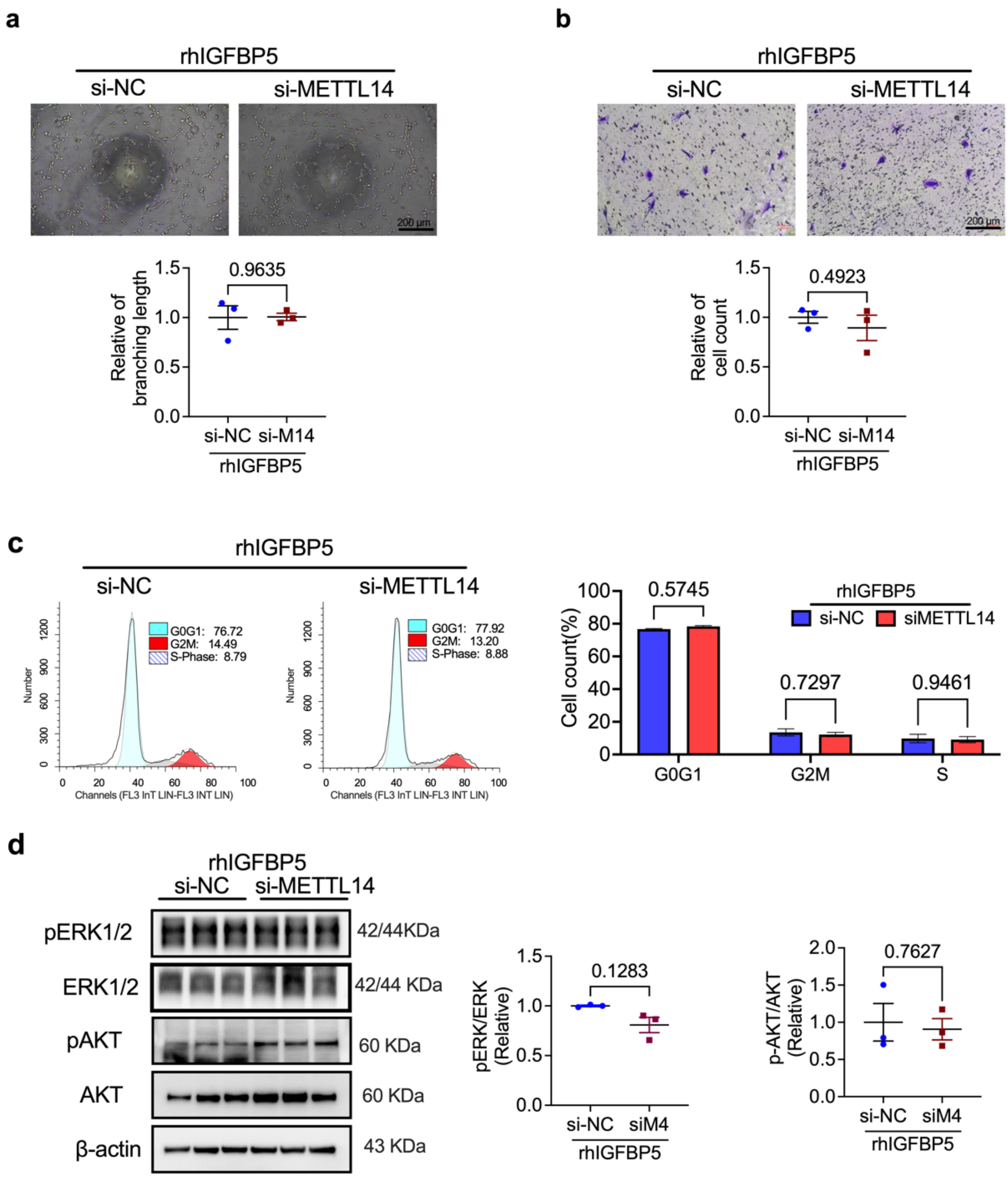

Supplementary Fig. 5. Knockdown of METTL14 neutralized recombinant IGFBP5-suppressed tube formation, proliferation, and migration. a. Tube formation images and quantification of HUVECs treated with recombinant IGFBP5 and transfected with si-control or si-METTL14 (n = 3 per group). **b,** Transwell assay images and quantification of HUVECs treated with recombinant IGFBP5 and transfected with si-control or si-METTL14 (n = 3 per group). **c,** flow cytometry for cell cycle images and quantification of HUVECs treated with recombinant IGFBP5 and transfected with si-control or si-METTL14 (n = 3 per group). **d,**

representative western blotting images and quantification of pERK1/2, ERK1/2, pAKT, and AKT in HUVECs treated with recombinant IGFBP5 and transfected with si-control or si-METTL14 (n = 3 per group).

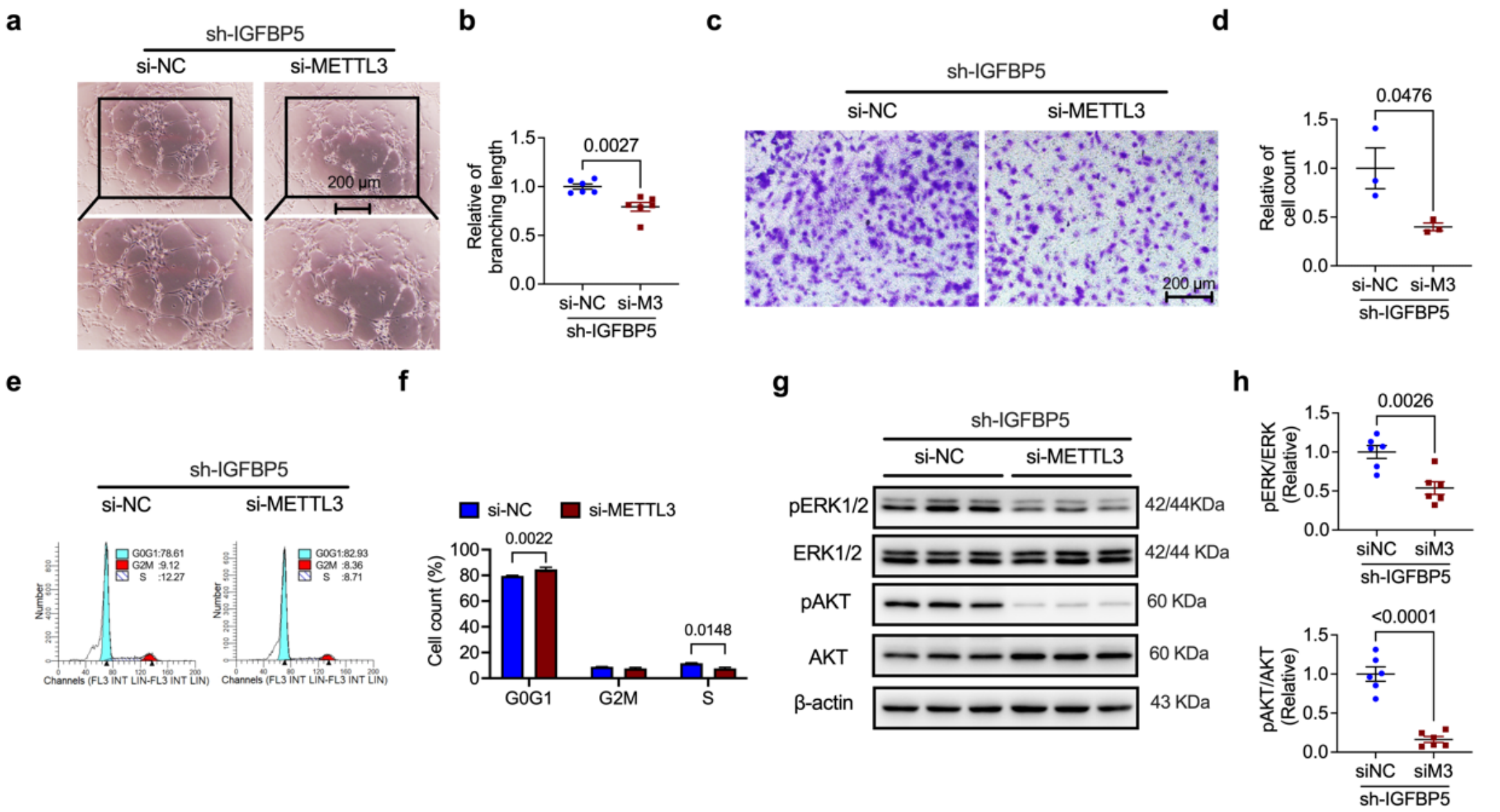

Supplementary Fig. 6. Knockdown of METTL3 suppressed IGFBP5 deficiency-promoted tube formation, cell proliferation, and migration in HUVEC. a, tube formation images, and **b,** quantification of IGFBP5 shRNA-infected HUVECs transfected with control siRNA (si-NC) and METTL3 siRNA (n = 6 per group). **c,** Transwell assay images, and **d,** quantification in IGFBP5 shRNA-infected HUVECs transfected with control siRNA (si-NC) and METTL3 siRNA (n = 3 per group). **e,** flow cytometry images, and **f,** quantification for cell cycle in IGFBP5 shRNA-infected HUVECs transfected with control siRNA (si-NC) and METTL3 siRNA (n = 3 per group). **g,** representative western blotting images, and **h,** quantification of p-ERK1/2, ERK1/2, p-AKT, and AKT expression in IGFBP5 shRNA-infected HUVECs transfected with control siRNA (si-NC) and METTL3 siRNA (n = 6 per group).

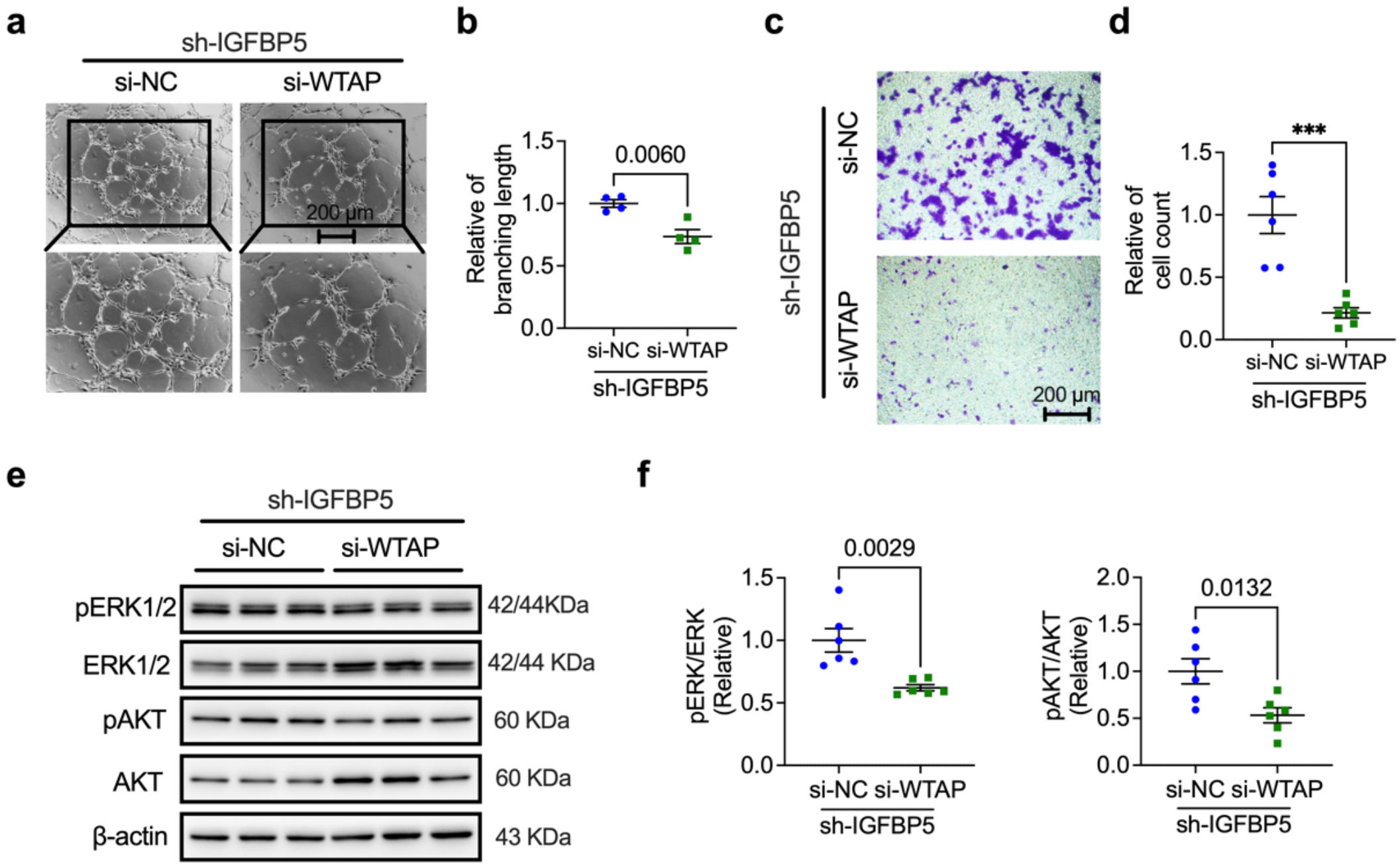

Supplementary Fig. 7. Knockdown of WTAP suppressed IGFBP5 deficiency-promoted tube formation and migration in HUVEC. a, tube formation images, and **b,** quantification of in IGFBP5 shRNA-infected HUVECs transfected with control siRNA (si-NC) and WTAP siRNA (n = 4 per group). **c,** Transwell assay images, and **d,** quantification of in IGFBP5 shRNA-infected HUVECs transfected with control siRNA (si-NC) and WTAP siRNA (n = 6 per group). **e,** western blotting images, and **f,** quantification of p-ERK1/2, ERK1/2, p-AKT, and AKT expression in IGFBP5 shRNA-infected HUVECs transfected with control siRNA (si-NC) and WTAP siRNA (n = 6 per group).

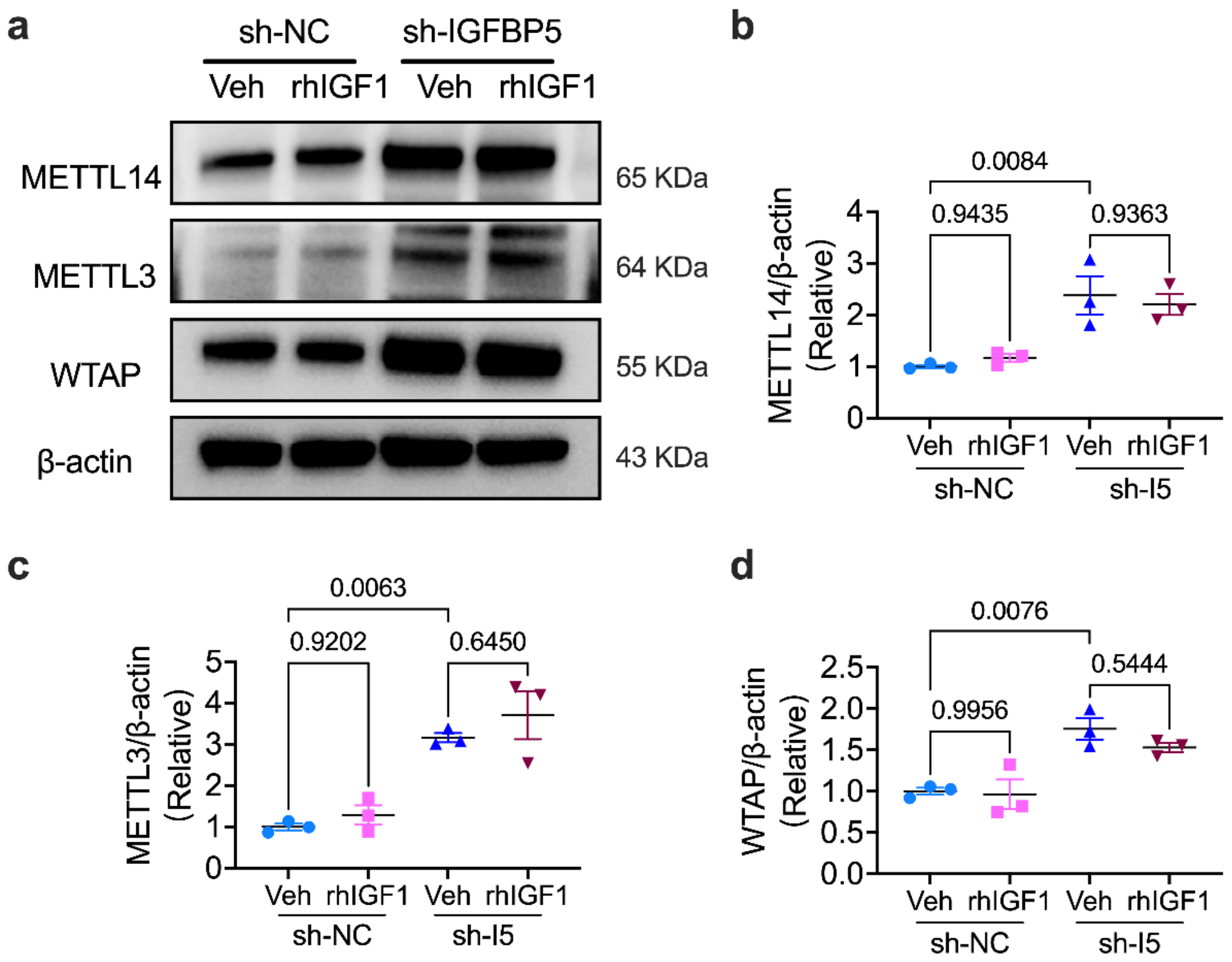

Supplementary Fig. 8. IGFBP5 knockdown-promoted expression of METTL14, METTL3, and WTAP was not suppressed by recombinant IGF1. a, western blotting images, and **b-d**, quantification of METTL14, METTL3, and WTAP in HUVECs treated with vehicle or recombinant IGF1 transfected with sh-control or sh-IGFBP5 (n = 3 per group).

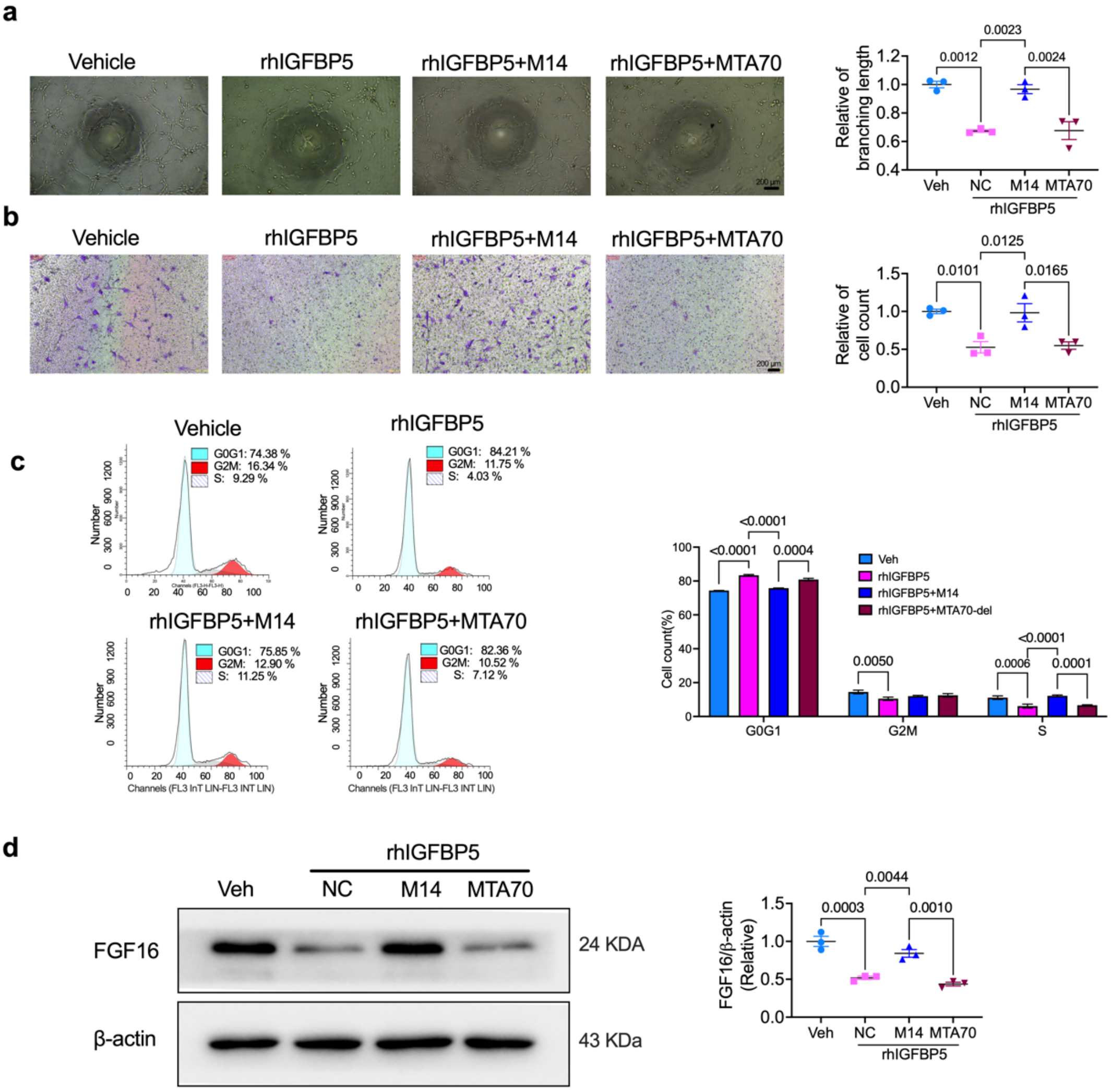

Supplementary Fig. 9. IGFBP5-suppressed tube formation, proliferation, and migration were rescued by METTL14, which was neutralized by mutation of MT-A70 sites of METTL14. a, tube formation images and quantification of HUVECs treated with recombinant IGFBP5 and infected with lentivirus overexpressing METTL14 or mutation of the MT-A70 site of METTL14 (n = 3 per group). **b,** Transwell assay images and quantification of HUVECs treated with recombinant IGFBP5 and infected with lentivirus overexpressing METTL14 or the MT-A70 site of METTL14 (n = 3 per group). **c,** flow cytometry for cell cycle images and quantification of HUVECs treated with recombinant IGFBP5 and infected with lentivirus overexpressing METTL14 or the MT-A70 site of METTL14 (n = 3 per group). **d,** representative

western blotting images and quantification of FGF16 protein of HUVECs treated with recombinant IGFBP5 and lentivirus overexpressing METTL14 or MTA70 site of METTL14 (n = 3 per group).

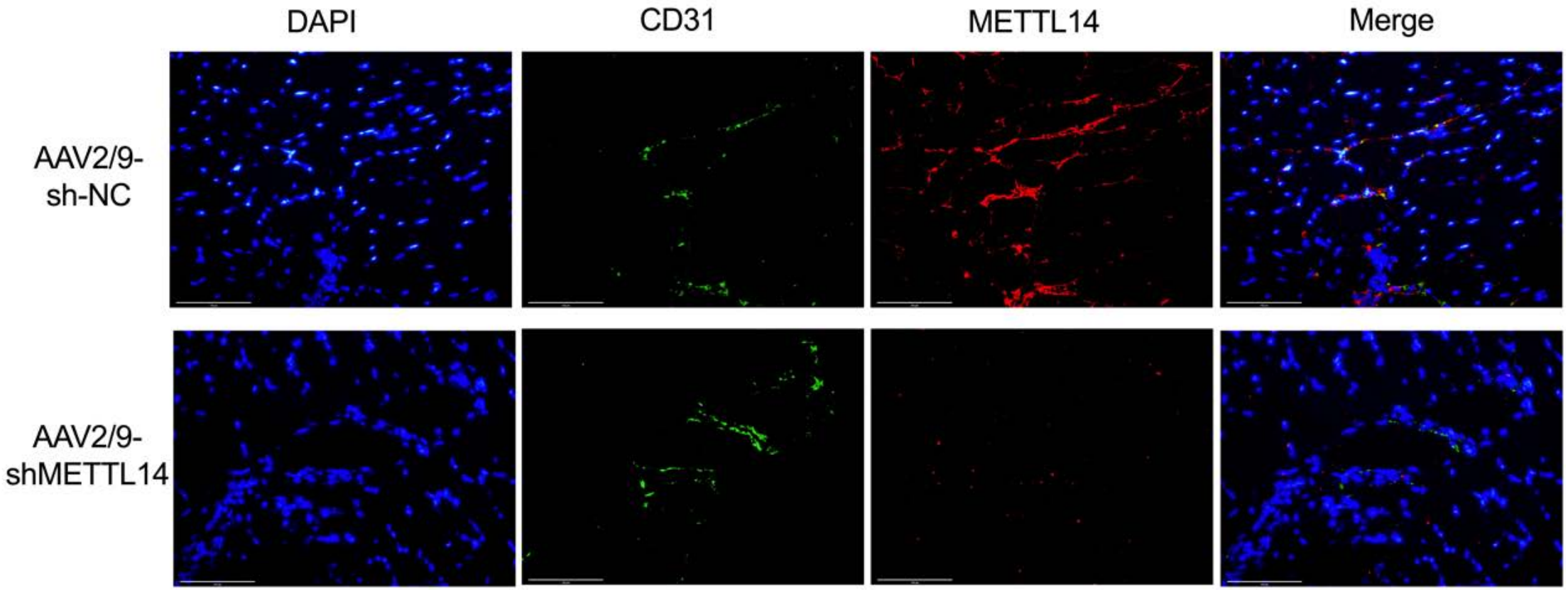

Supplementary Fig. 10. METTL14 was decreased in endothelial cells of AAV2/9-sh-METTL14-infected gastrocnemius. Gastrocnemius were stained with CD31 and METTL14 antibodies.

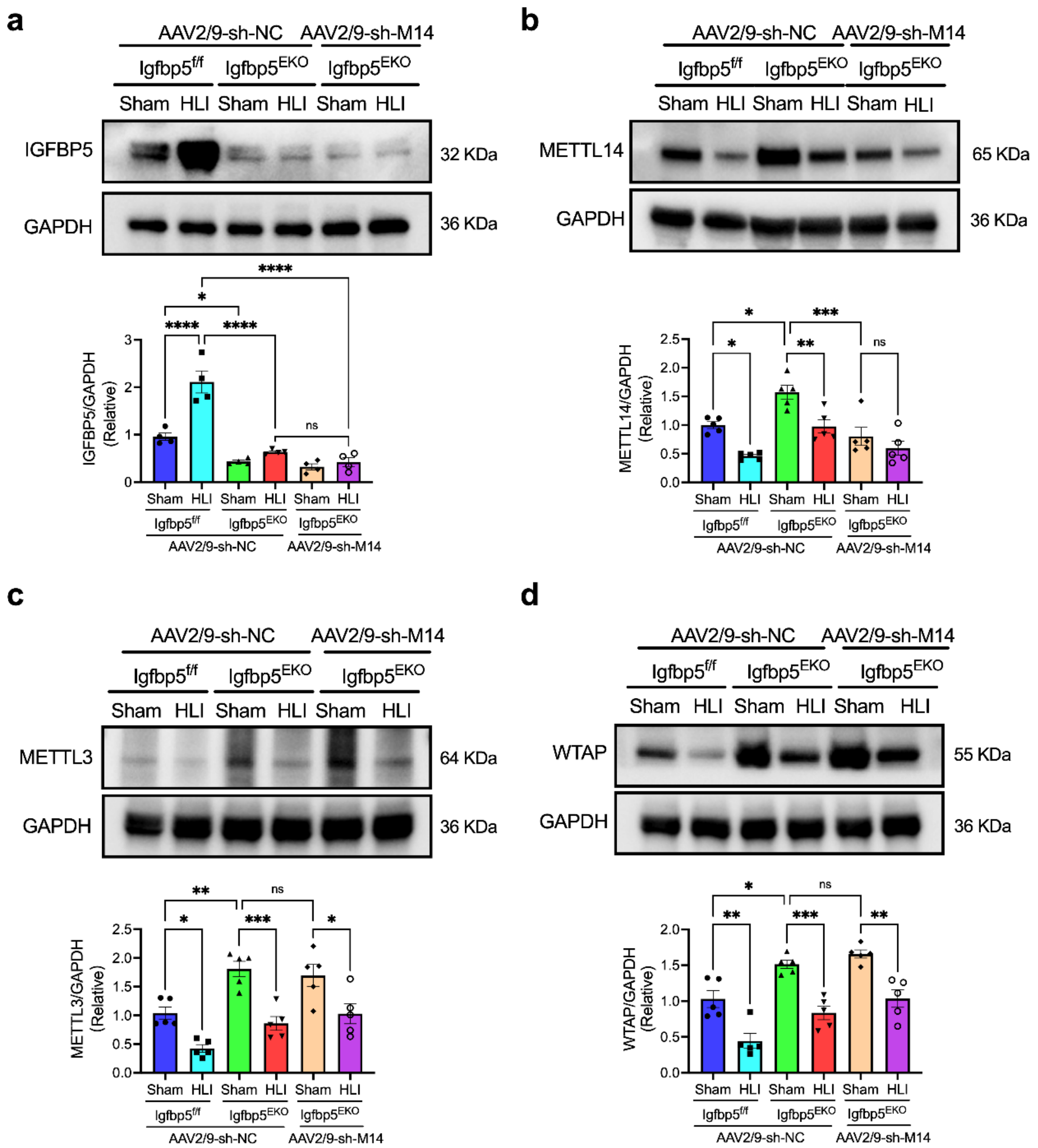

Supplementary Fig. 11. Expression of METTL14, METTL3, and WTAP in vivo. Representative western blotting images and quantification of IGFBP5 (**a**), METTL14 (**b**), METTL3 (**c**), and WTAP (**d**) in gastrocnemius of AAV2/9-control shRNA- and AAV2/9-Mettl14 shRNA-infected Igfbp5^{EKO} and Igfbp5$^{f/f}$ mice treated with sham or HLI.

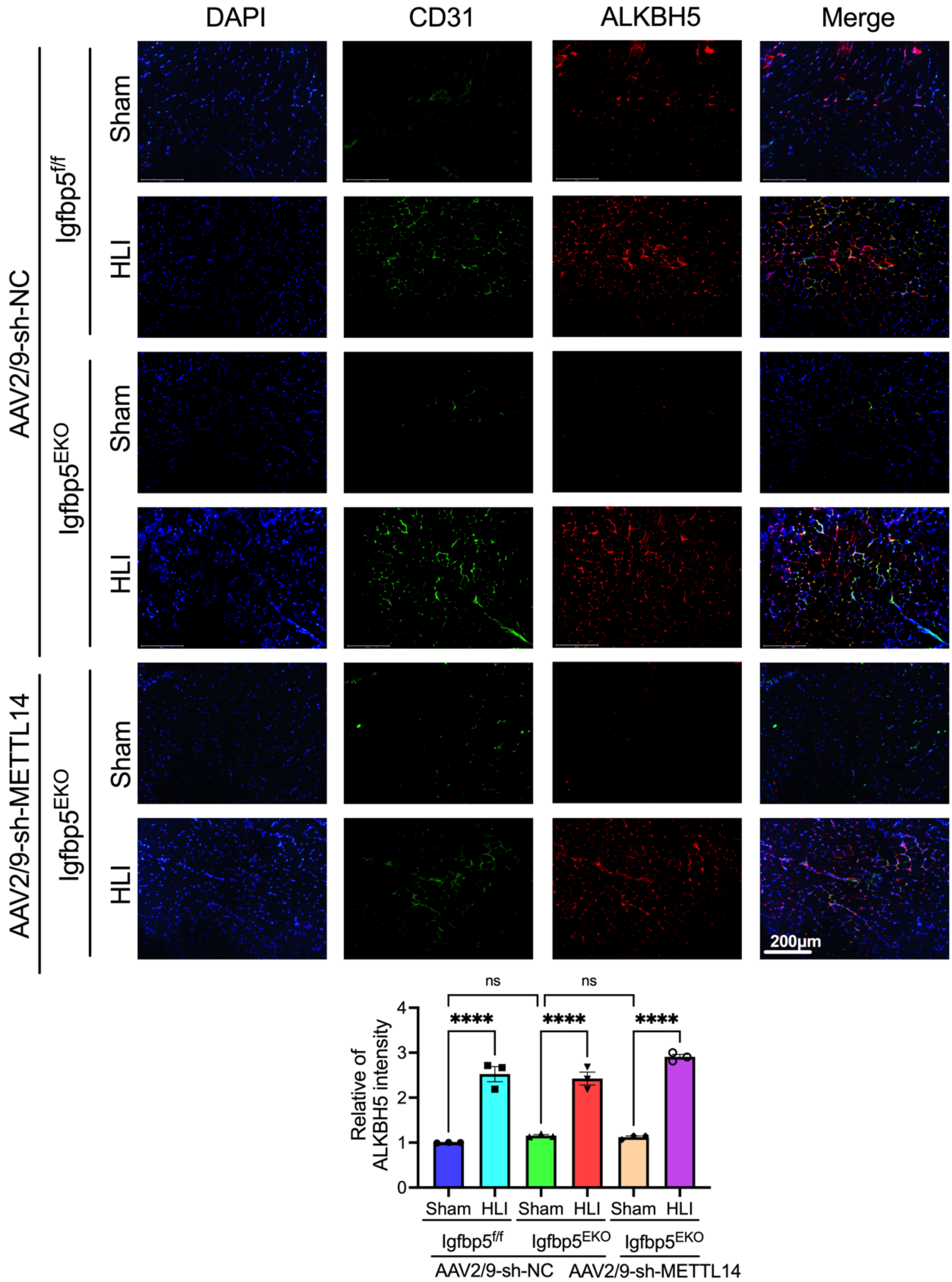

Supplementary Fig. 12. Immunofluorescence staining of ALKBH5 in mouse hindlimb. Immunofluorescence staining of CD31 (green) and ALKBH5 (red) in gastrocnemius of AAV2/9-control shRNA- and AAV2/9-Mettl14 shRNA-infected Igfbp5^{EKO} and Igfbp5$^{f/f}$ mice treated with sham or HLI.

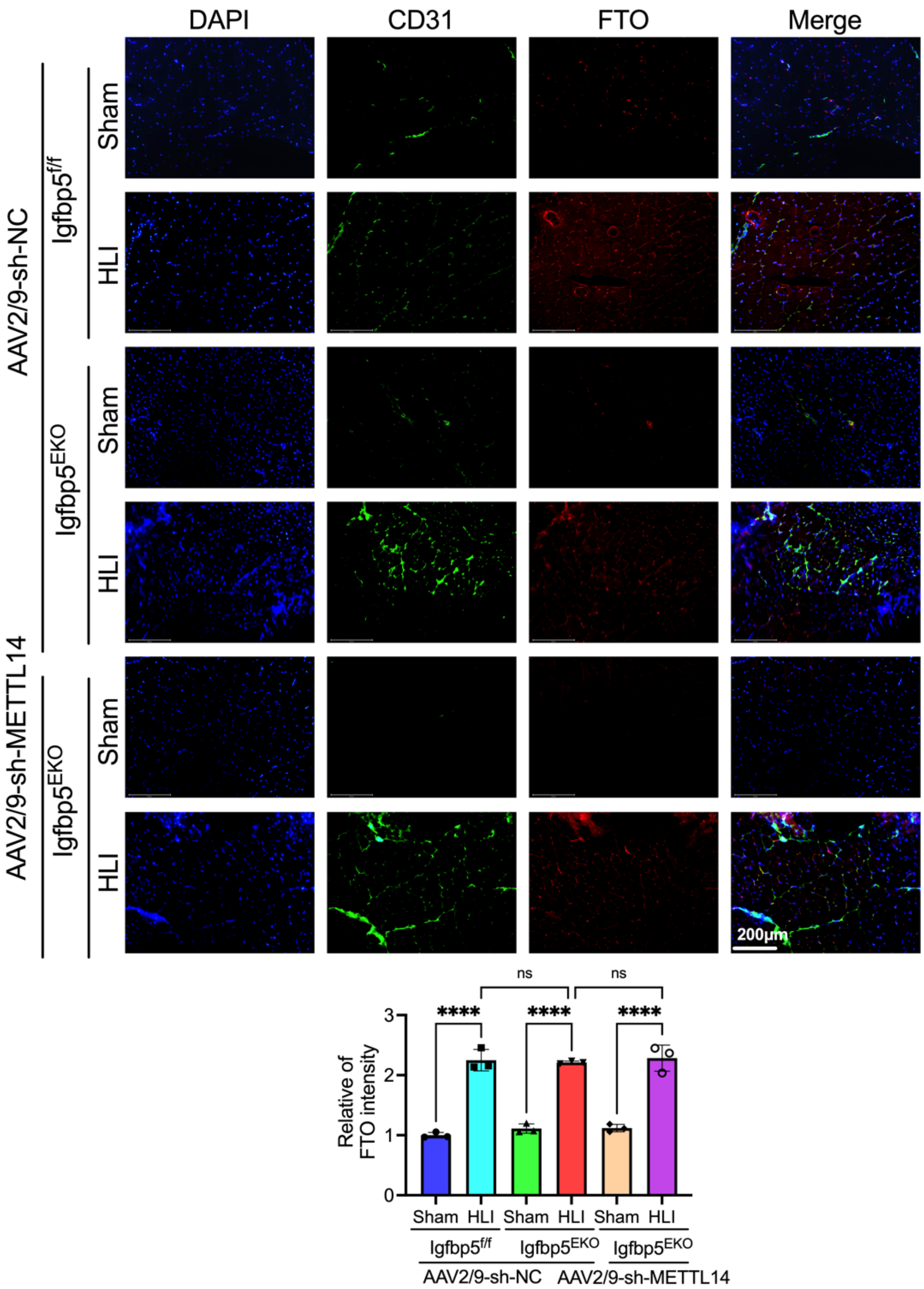

Supplementary Fig. 13. Immunofluorescence staining of FTO in mouse hindlimb. Immunofluorescence staining of CD31 (green) and FTO (red) in gastrocnemius of AAV2/9-control shRNA- and AAV2/9-Mettl14 shRNA-infected Igfbp5^{EKO} and Igfbp5$^{f/f}$ mice treated with sham or HLI.

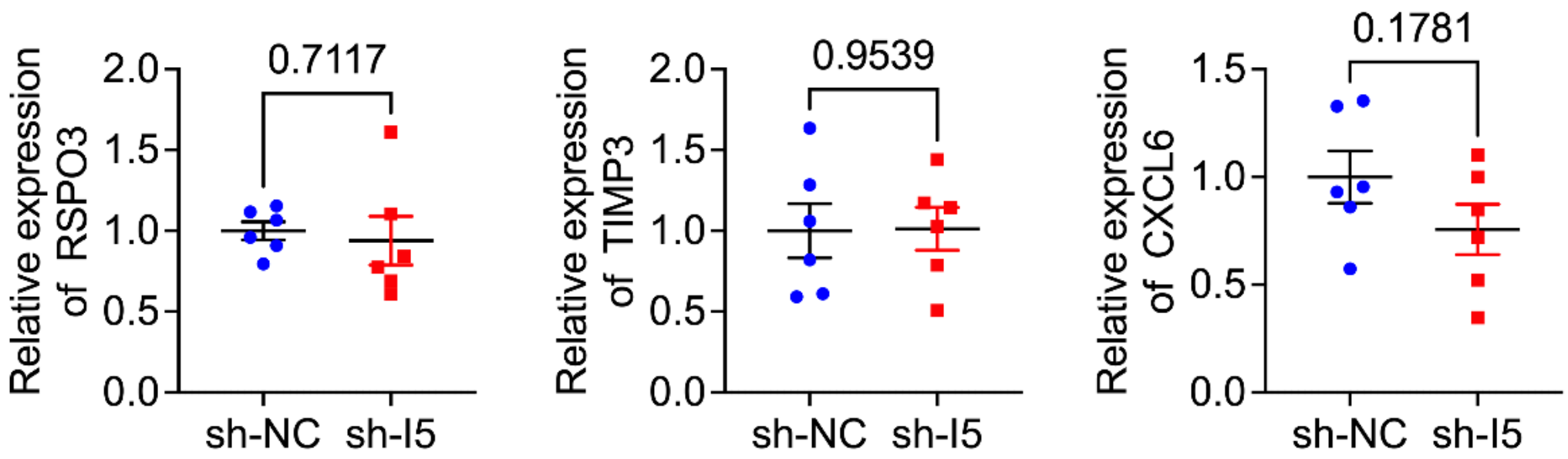

Supplementary Fig. 14. Validation of up-regulated genes identified in RNA-seq. The qPCR assay determined the mRNA levels of RSPO3, TIMP3, and CXCL6 in the sh-control- or sh-IGFBP5-transfected HUVECs.

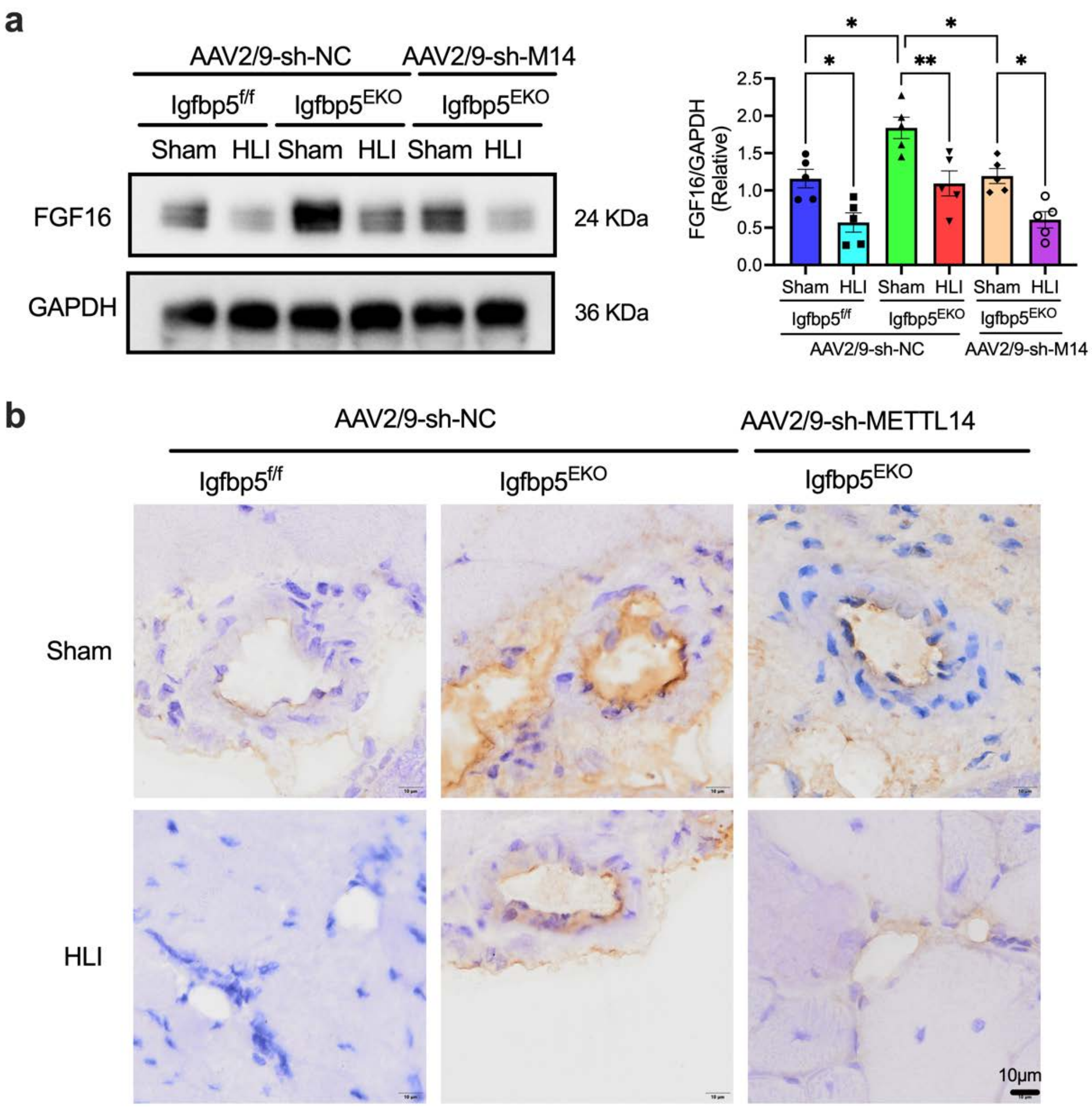

Supplementary Fig. 15. Expression of FGF16 in vivo. a, Representative western blotting images and quantification of FGF16 in gastrocnemius of AAV2/9-control shRNA- and AAV2/9-Mettl14 shRNA-infected Igfbp5^{EKO} and Igfbp5$^{f/f}$ mice treated with sham or HLI. **b**, Immunohistochemistry staining of FGF16 in gastrocnemius of AAV2/9-control shRNA- and AAV2/9-Mettl14 shRNA-infected Igfbp5^{EKO} and Igfbp5$^{f/f}$ mice treated with sham or HLI.

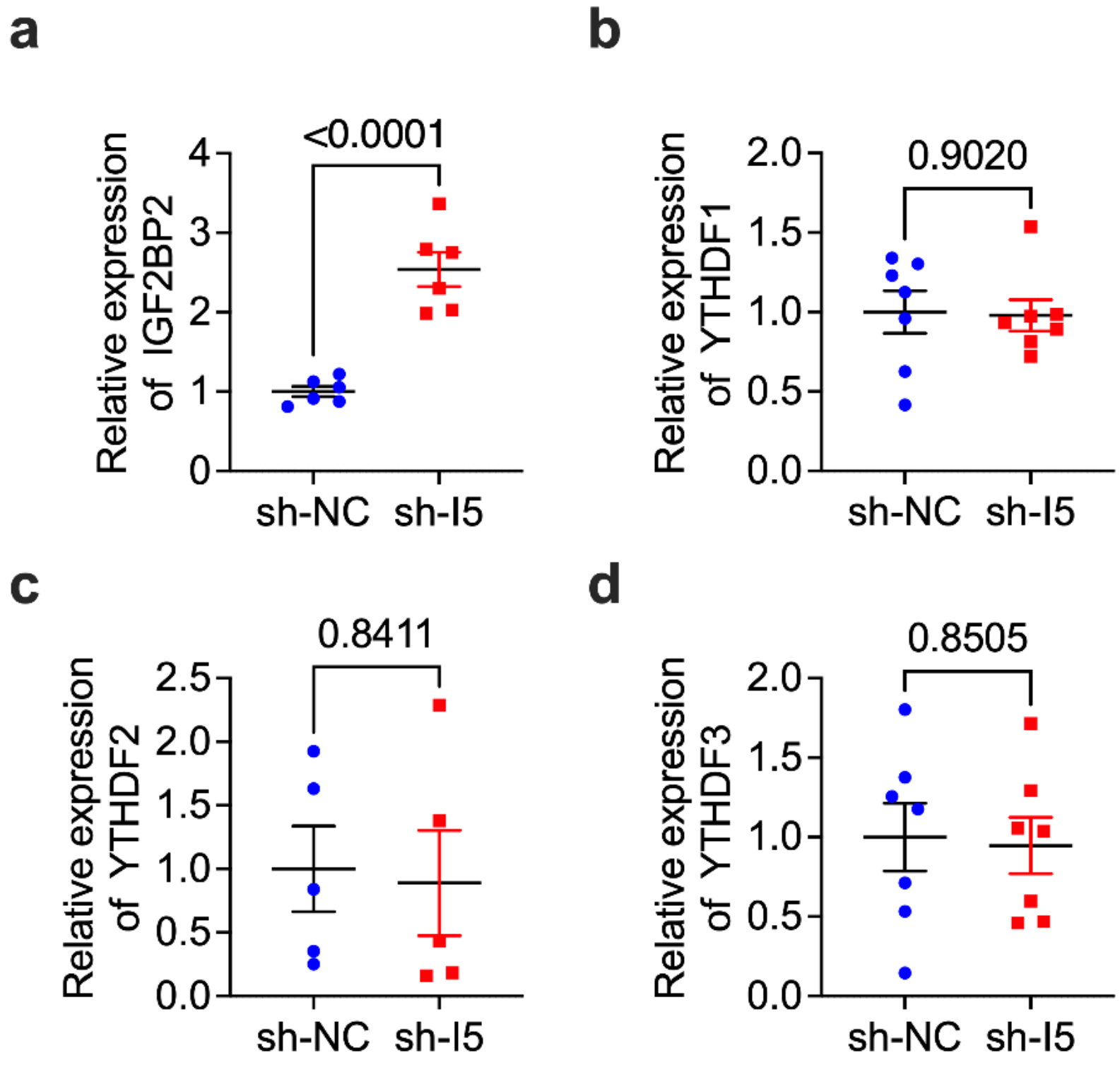

Supplementary Fig. 16. mRNA levels of m6A readers in sh-IGFBP5-infected HUVECs.

The qPCR assay determined the mRNA levels of IGF2BP2 (**a**), YTDHF1 (**b**), YTDHF2 (**c**), and YTDHF3 (**d**) in the sh-control- or sh-IGFBP5-infected HUVECs.

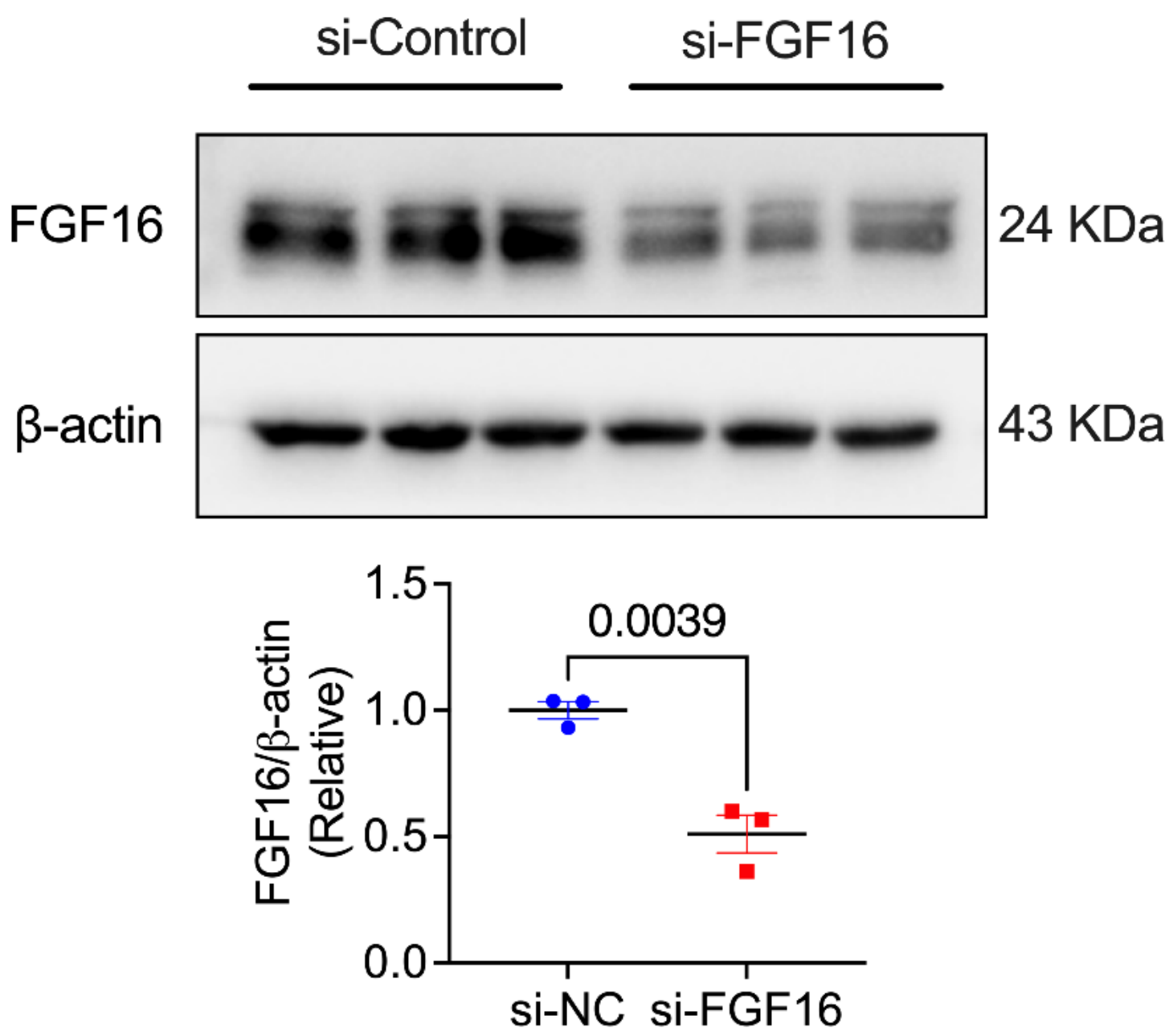

Supplementary Fig. 17. Knockdown efficiency of FGF16. The western blotting assay determined the protein levels of FGF16 in the si-control- or si-FGF16-transfected HUVECs.

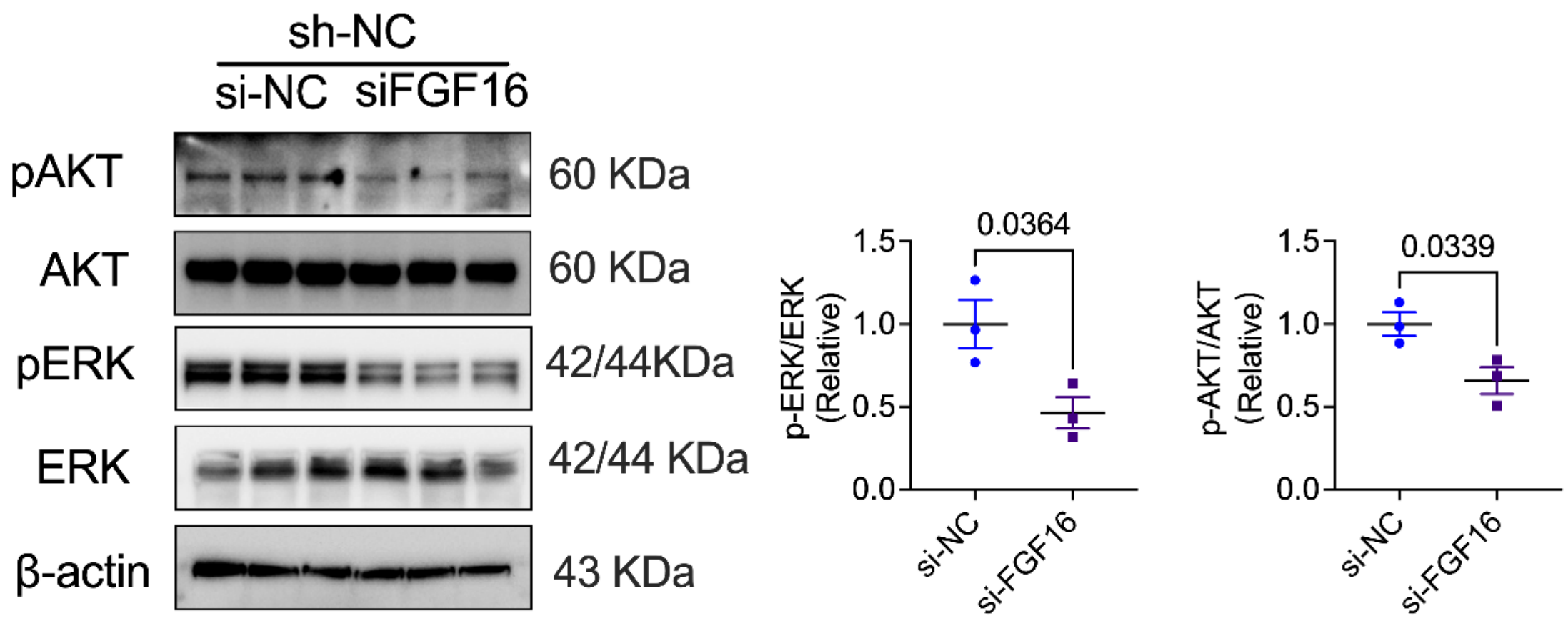

Supplementary Fig. 18. FGF6 knockdown suppressed phosphorylation of ERK1/2 and AKT. Western blotting images and quantification of phosphorylation of ERK1/2 and AKT in HUVECs transfected with si-control or si-FGF16 (n = 3 per group).

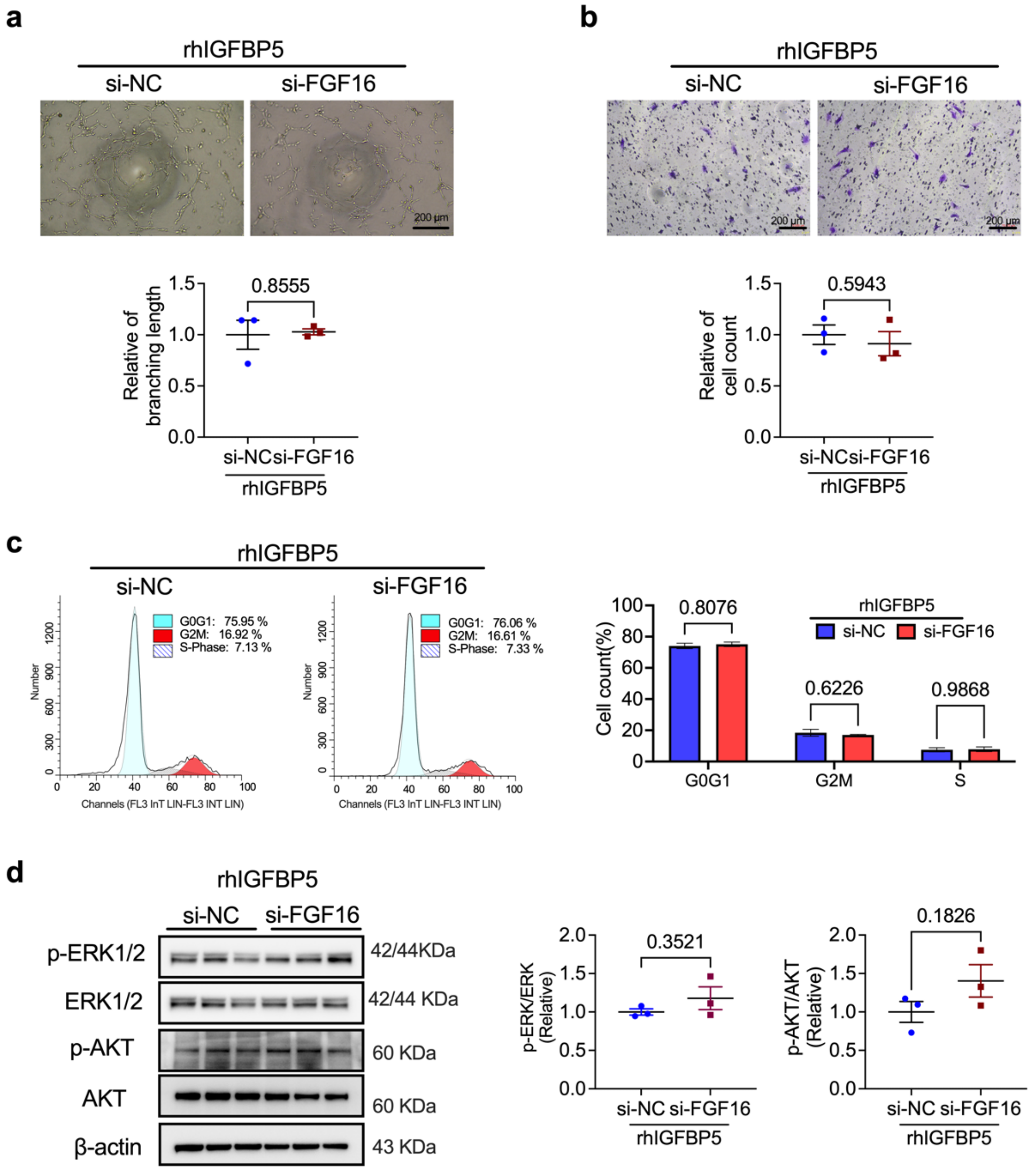

Supplementary Fig. 19. Knockdown of FGF16-suppressed tube formation, proliferation, and migration were neutralized by rhIGFBP5. a, tube formation images and quantification of si-control- or si-FGF16-transfected HUVECs treated with rhIGFBP5 (n = 3 per group). **b,** Transwell assay images and quantification of si-control- or si-FGF16-transfected HUVECs treated with rhIGFBP5 (n = 3 per group). **c,** flow cytometry images and quantification of cell cycle in si-control- or si-FGF16-transfected HUVECs treated with rhIGFBP5 (n = 3 per group). **d,** representative western blotting images and quantification of p-ERK1/2 and p-AKT

expression in si-control- or si-FGF16-transfected HUVECs treated with rhIGFBP5 (n = 3 per group).

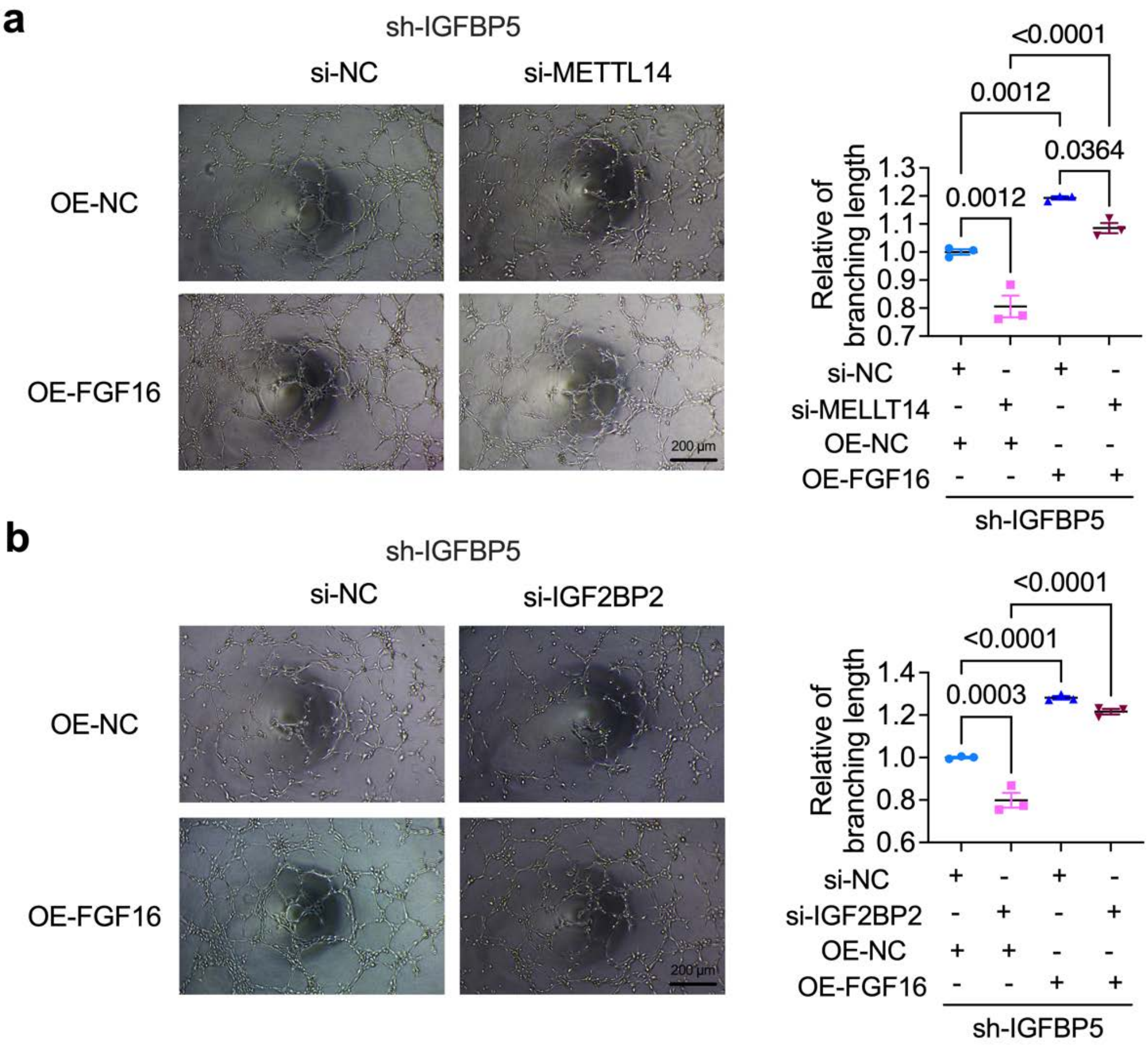

Supplementary Fig. 20. Knockdown IGFBP5-promoted tube formation was suppressed by METTL14 or IGF2BP2 siRNA and rescued by overexpression of FGF16. a, tube formation images and quantification of sh-IGFBP5-infected HUVECs transfected with si-control or si-METTL14 and/or FGF16 overexpression lentivirus or control lentivirus (n = 3 per group). **b,** tube formation images and quantification of sh-IGFBP5-infected HUVECs transfected with si-control or si-IGF2BP2 and/or FGF16 overexpression lentivirus or control lentivirus (n = 3 per group).
