# Supplementary material for: METTL14-dependent N6-methyladenosine modification of FGF16 participates in IGFBP5 deficiency-enhanced angiogenesis
Source: Exp Mol Med. 2026 Aug 4;58(8):2630–42. doi: 10.1038/s12276-026-01788-y (PMC13538678; doi:10.1038/s12276-026-01788-y)
Supplement: Supplementary file 2 [file 12276_2026_1788_MOESM2_ESM.pdf]

Supplementary Table 1. Sequence of FGF16 siRNA

Name	sequence
FGF16-homo-850 sense	GGCCUGUAGAUCCUUCUAATT
FGF16-homo-850 antisense	UUAGAAGGAUCUACAGGCCTT
FGF16-homo-619 sense	GCCUGUACCUAGGAAUGAATT
FGF16-homo-619 antisense	UUCAUUCCUAGGUACAGGCTT
FGF16-homo-690 sense	CGGGAACAGUUUGAAGAAATT
FGF16-homo-690 antisense	UUUCUUCAAACUGUUCCCGTT

Supplementary Table 2. Primer sequence of probes for different m6A sites of FGF16

Gene	primer sequences
FGF16-55 (probe)	L:5'po4CCACTCCCCGGACAATAACAACACGGTAGCAGGAGT3’<br>R:5’ ACCTGGACCACTTGGAACATATTGGGTACAGGCCAGArGrU3’
FGF16-203 (probe)	L:5'po4CTCTCTGAGTCCGAAACAGCCGAAGAA CCTTTACC3’<br>R:5’TCTGGTGGCATCACTCATCTGCCACGTAAT ACTrGrU3’
FGF16-control (probe)	L:5'-po4TTCCAAAGCTCTGTAGAAATCAACCCAATCAGACCAAT-3’;<br>R:5’-CATGATTAGCACCTGTTTGAGCATTTTCTTAATGAGACrTrU 3’
FGF16-55	F:5'-ACTCCTGCTACCGTGTTGTTATTG-3’<br>R:5’-ACCTGGACCACTTGGAACATATTG-3’
FGF16-203	F:5'-GGTAAAGGTTCTTCGGCTGT-3’<br>R:5’-TCTGGTGGCATCACTCATCT-3’
FGF16-control	F:5'-ATTGGTCTGATTGGGTTGATTT-3’<br>R:5’-CATGATTAGCACCTGTTTGAGC-3’

Supplementary Table 3. shIGFBP5 vs shNC (hyper-up)

Transcript ID	Gene Symbol	Expresstion		Methyation	
		shIGFBP5-group(mean)	shNC-group(mean)	shIGFBP5-group(mean)	shNC-group(mean)
uc001zip.3	GOLGA8B	3.0196782	2.4097167	3.0645743	2.3576107
ENST00000300730	PGAP2	-2.930812	-3.8611306	-3.0624992	-3.9777004
ENST00000540140	UTY	-2.850664	-3.4544459	-2.9292129	-3.7556879
ENST00000617612	C11orf96	0.7103874	-0.1286427	0.744517	-0.1848827
ENST00000233809	IGFBP2	-0.4920109	-1.2053687	-0.492922	-1.3177865
ENST00000374017	SNRPC	-2.7418405	-3.5643181	-3.1181119	-4.6037317
ENST00000228938	MGP	-0.2005026	-1.1908182	-0.1986651	-1.2557517
ENST00000392876	NT5DC3	-2.602297	-3.2659875	-2.6865891	-3.5282178
ENST00000219478	ZNF500	-2.6243271	-3.2931381	-2.6512606	-3.4148868
ENST00000505747	THUMPD2	-1.9418158	-2.5866198	-1.946327	-2.6829561
ENST00000508576	HOMER1	-1.8945011	-2.5684802	-1.9115577	-2.6641066
ENST00000370575	PYROXD2	-2.6324473	-3.3562801	-2.6630376	-3.499932
ENST00000429504	CERS1	-0.2944267	-1.0314334	-0.266646	-1.1007012
ENST00000272164	WNT9A	0.6044001	-0.0251978	0.6386637	-0.0802457
ENST00000545173	EIF3K	-2.7219514	-3.4660757	-2.7559778	-3.6193473
ENST00000352983	RPL7	-0.3127528	-0.9448423	-0.3116009	-1.0351962
ENST00000328933	SYNGR1	0.1434267	-0.6759306	0.1173676	-0.9274542
ENST00000267953	BCL2A1	-1.5178623	-2.1033099	-1.5120499	-2.1875001
ENST00000510585	CTC-534A2.2	-2.6242712	-3.4453247	-2.6564619	-3.578053
ENST00000340650	NHLRC1	-0.2780951	-0.9946689	-0.2488382	-1.060293
ENST00000392250	DPY19L3	-2.7375388	-3.390204	-2.7681202	-3.480053
ENST00000448022	MDFIC	-2.7211593	-3.3440493	-2.7559973	-3.4976124
ENST00000286544	FAM161B	-1.5924432	-2.2085793	-1.6068035	-2.3205192
ENST00000394890	P4HA1	-1.0056106	-1.7677728	-1.1013238	-2.1651952
ENST00000465393	TNIK	-2.3928496	-3.4332189	-2.435623	-3.6112587
ENST00000444304	C1QTNF7	-2.7000159	-3.304857	-2.7736452	-3.4845585
ENST00000415986	GPRASP1	-2.5661153	-3.2077569	-2.8924223	-3.5780915
ENST00000404169	CAMKK2	-2.0274363	-2.7026551	-2.070828	-2.8043159

ENST00000377079	LMAN2L	-2.9006641	-3.5487672	-2.9812852	-3.710969
ENST00000622804	ELOC	-2.8236668	-3.4618809	-2.8655719	-3.6052172
ENST00000392429	TMEM200A	-2.6126476	-3.3790561	-2.6407588	-3.5152133
ENST00000408995	FHL2	-1.6568545	-2.3235475	-1.6630201	-2.4757757
ENST00000563232	EARS2	-1.8865007	-2.6160237	-1.9051112	-2.7282964
ENST00000404488	CRELD2	0.4172676	-0.2423404	0.4379279	-0.309201
ENST00000562435	NETO2	1.7412149	1.0429409	1.7872955	0.9905038
ENST00000616481	PHOSPHO2	-2.7387191	-3.4967447	-2.7731439	-3.6457259
NM_001329451	KANK2	-0.8620485	-1.4949538	-0.8399459	-1.5683483
ENST00000277198	C9orf3	-1.8001354	-2.5055583	-1.8323884	-2.6391876
ENST00000619447	FGF1	-2.8331324	-3.5185299	-2.8810361	-3.7712198
ENST00000317814	HES7	-2.484286	-3.191128	-2.5034805	-3.3050445
MICT00000178407	CATG00000039615.1	-2.2631656	-2.9516257	-2.2869853	-3.0571461
ENST00000586956	TMEM205	-1.983847	-2.5832539	-2.2039034	-3.011023
ENST00000465503	STIM2	-1.5130169	-2.1810261	-1.505494	-2.2776444
ENST00000506382	WI2-2373I1.1	-2.2734376	-2.8904298	-2.7238856	-3.7388742
ENST00000317551	CERS5	-0.6651421	-1.280046	-0.6347939	-1.345724
ENST00000354377	ZNF774	-1.7386474	-2.4557144	-1.7708317	-2.6634848
ENST00000311672	UQCRH	-1.5857847	-2.4017404	-1.6821084	-2.7586029
NM_001310321	MFGE8	-1.0818132	-1.6938904	-1.1116749	-1.888681
ENST00000277540	DPH7	-1.9984561	-2.6362362	-2.0066245	-2.7806132
ENST00000315264	KIF26A	1.2584736	0.3398439	1.3048021	0.2834131
ENST00000505726	HSPA4L	-2.7342687	-3.3673876	-2.7677244	-3.4832003
ENST00000629778	SYT14	-2.7162984	-3.7444513	-2.7518865	-3.9965703
ENST00000249042	TST	-2.4122812	-3.3513167	-2.4328433	-3.4743739
ENST00000397560	KDM7A	-2.6706268	-3.3313155	-2.6988232	-3.4466946
ENST00000262464	FBN2	0.6793695	-0.5393869	0.7173642	-0.599624
ENST00000618628	ENC1	-1.2139676	-1.8550911	-1.1993374	-1.9390193
ENST00000417956	CELF2	-0.4070131	-1.4275514	-0.7018263	-1.6421102
ENST00000261819	ANAPC5	-2.4034831	-3.0812837	-2.4884415	-3.2646913
ENST00000296027	CXCL5	-2.3821103	-3.3067819	-2.3999928	-3.4353231
NM_001348191	ABCG4	-2.8423499	-3.4691622	-2.8826484	-3.594726

NM_001286705	UFM1	-1.656477	-2.3980723	-1.6502127	-2.4850587
ENST00000337566	POLR3H	-1.0299545	-1.6265693	-1.0308805	-1.764689
uc002jww.4	ENGASE	-1.7158058	-2.8883787	-1.7155859	-2.9915322
ENST00000612452	AR	-1.2671312	-2.3544458	-1.2572061	-2.4393509
ENST00000258111	KCNMB4	-0.9973298	-1.648318	-0.9793057	-1.7513144
ENST00000327555	MCAT	-2.8909618	-3.5577452	-2.9568306	-3.7894733
ENST00000310754	TRAM1L1	-1.0927401	-2.1510118	-1.0751944	-2.4293376
ENST00000357248	SMARCA2	-2.3009959	-3.3387649	-2.3272241	-3.5057727
ENST00000379980	MYH10	1.7402335	1.1484607	1.7859716	1.0950347
ENST00000311507	PLAC8	2.4862633	1.5555806	2.471613	1.3975478
ENST00000428135	SNX13	-0.9973821	-1.6038537	-0.9748292	-1.6725289
ENST00000536220	ZNF573	2.6654182	2.0670305	2.0379705	1.2374627
ENST00000429375	POU2F1	-2.543835	-3.3204163	-2.6179451	-3.4535365
NM_001317931	SPAG4	-2.2539059	-2.8442123	-2.2763911	-2.9495118
ENST00000457527	HARS	-1.3746187	-1.9729734	-1.3701412	-2.0615954
ENST00000538627	ARMCX6	-2.8157037	-3.6125214	-2.9282769	-3.86113
ENST00000293471	ZNF613	-1.8623187	-2.5929964	-1.8682943	-2.6911041
ENST00000336798	SDC3	0.0678435	-0.6465299	0.0492954	-0.7940805
ENST00000371764	DDX27	-1.1608063	-1.8148324	-1.1446674	-1.8897701
ENST00000308511	KIAA1967	-2.7080611	-3.3343849	-2.7764639	-3.4936768
ENST00000429576	MCTP1	1.6700181	0.7945861	1.7092375	0.743876
ENST00000307365	DDIT4	5.7962577	5.1746015	5.7855951	5.1418126
ENST00000318094	TOM1L2	-2.5135001	-3.2503672	-2.7634352	-3.7945409
ENST00000595830	ZNF418	-2.563974	-3.774553	-2.7037382	-3.9439623
ENST00000439435	FGF16	0.0383529	-2.082244	0.0718593	-2.1614178
ENST00000572940	PLD2	-2.0333722	-2.6417919	-2.0393337	-2.7389126
ENST00000563094	UTP4	-2.1990403	-3.003192	-2.2099643	-3.1103399
ENST00000361970	CCDC152	-1.5427287	-2.517554	-1.5375627	-2.6223333
NM_001350641	RP11-65B7.2	-2.0003988	-2.8336042	-2.0192209	-3.0087102
ENST00000276708	GSDMC	-2.6740223	-3.2710321	-2.8352471	-3.6646601
ENST00000374807	LAS1L	-1.6540722	-2.5659521	-1.654921	-2.660191
ENCT00000328189	CATG00000071657.1	-2.2210528	-2.8379783	-2.5320426	-3.3689623

ENST00000617930	CNOT3	-1.9588873	-2.5680194	-2.0260389	-2.7214425
NM_001352498	ZNF75A	-2.715197	-3.3445954	-2.7486754	-3.4610587
ENST00000505890	DDX60L	-2.3514637	-3.1279936	-2.3718462	-3.2463314
ENST00000580435	STRA13	-2.3510487	-3.0211483	-2.4193705	-3.1305288
ENST00000287272	UNC5D	-2.7502909	-3.4375416	-2.7846811	-3.5634242
ENST00000227638	PANX1	-1.3384854	-1.9710664	-1.32708	-2.0447834
NM_001271004	TFPI2	0.100993	-0.8372642	0.1224537	-0.9113974
ENST00000339431	LRRC17	0.5348171	-0.2841503	0.5556201	-0.383556
ENST00000322128	TSPYL5	1.0296824	0.4168275	1.0202129	0.2879542
ENST00000359942	IPP	-2.6490953	-3.6751203	-2.6820209	-3.8309474
ENST00000356698	RSPO3	-2.4174298	-3.6215623	-2.445414	-3.7795157
ENST00000442628	NRBP2	-1.9589091	-2.548659	-1.9909134	-2.6811824
ENST00000369063	RTN4IP1	-1.2896208	-1.8832856	-1.2789552	-1.9589909
ENST00000460540	IP6K1	-2.0997757	-2.7209787	-2.1073713	-2.8189831
ENST00000458307	AMT	0.8086128	0.1520874	0.844304	0.0931023
ENST00000244050	SNAI1	1.7264893	0.9002386	1.7711382	0.8487854
NM_001145961	SLC12A4	-1.0910749	-1.8250564	-1.0920324	-1.9015962
ENST00000215727	SERPIND1	-1.3534487	-1.9560414	-1.3565237	-2.0396701
ENST00000581136	NAT9	-2.4181683	-3.2325449	-2.4786905	-3.5104271
ENST00000582707	USP14	0.2670233	-1.7698362	-0.1716444	-2.3105397
ENST00000431660	SRI	-2.4752817	-3.148501	-2.4973012	-3.2640282
ENST00000292408	FGFR4	-1.6200449	-2.2112355	-1.6214936	-2.3212214
ENST00000330501	ZNF397	-2.6663359	-3.4113942	-2.7067915	-3.5376017
ENST00000312584	TNFRSF10D	4.533183	3.8678492	4.5601721	3.8280465
ENST00000445217	ZNF793	-2.8900198	-3.5343358	-2.9418582	-3.7290676
ENST00000589699	MAPRE2	-2.6798322	-3.4344272	-2.8840647	-3.9974025
uc002uwp.2	NIF3L1	-2.9485151	-3.6688436	-3.0299655	-3.7625181
ENST00000366534	C1orf101	-1.9491042	-2.6211349	-1.9545431	-2.7158648
ENST00000304735	VASN	-2.4248342	-3.0479318	-2.4439638	-3.1620954
ENST00000393543	MDM1	0.3599475	-0.8466359	-0.0907708	-1.2890941
NM_001324340	ZSCAN32	-1.8300515	-2.4311182	-1.8338441	-2.520481
ENST00000356341	PAK1	-1.943598	-2.6009965	-1.9475855	-2.6983544
ENST00000221455	CLASRP	-0.8155295	-1.5085579	-0.8079362	-1.6239062

ENST00000310677	ADCY4	2.795122	2.1840359	2.7989534	2.0831842
ENST00000329106	SULT1C3	-2.314233	-2.9251004	-2.4131719	-3.0910054
ENST00000527911	DGKZ	-1.0515102	-1.6551495	-1.0519931	-1.7473754
ENST00000382899	KDM6A	-2.7467934	-3.4899322	-2.7881247	-3.645811
ENST00000476950	DSCR3	-0.4581114	-1.4711068	-0.7684145	-1.7309232
ENST00000375457	PCID2	-2.5965874	-3.2514054	-2.6475026	-3.3881433
ENST00000264039	GPC1	1.5910617	0.7913726	1.6106295	0.7303005
ENST00000426923	PLAC8	-0.7509375	-1.5626721	-0.8075277	-1.8504099
ENST00000222543	TFPI2	0.3739751	-0.7391304	0.4121779	-0.7998546
uc010gnx.3	ERG	-1.6484703	-3.1876659	-1.7307423	-3.4468513
ENST00000368912	SEMA6C	-2.2234636	-2.8246112	-2.2716876	-3.0375249
ENST00000432072	FN1	4.1477057	3.5481918	4.1674679	3.4902905
ENST00000344095	TAF6	0.9349218	-0.057451	0.2162973	-1.437873
ENST00000272224	GDF7	-1.0274485	-1.9790154	-1.0120125	-2.056624
ENST00000256362	VRTN	-1.6796347	-2.2993702	-1.6762021	-2.4538948
ENST00000334082	HOMER1	-1.7642503	-2.6062431	-1.7663154	-2.7017446
ENST00000368115	FCER1A	-2.5656956	-3.5640024	-2.9784738	-4.438483
ENST00000221448	SNRNP70	1.7078138	0.9580266	1.7522694	0.9047948
ENST00000295927	PTX3	6.7299126	5.8857862	6.7049298	5.8889158
ENST00000374045	TNFSF15	-0.5109611	-1.3703401	-0.5996244	-1.7442629
ENST00000260653	SIX3	-0.5959039	-3.9560038	-1.081214	-4.2371921
ENST00000372764	PLAU	-1.8257882	-2.4302169	-1.8281135	-2.5211778
ENST00000379410	PLEKHN1	-2.3497854	-3.1640211	-2.4040339	-3.2913331
ENST00000409382	ST6GAL2	-1.7270259	-2.6712606	-1.7687685	-2.7701499
ENST00000264703	FNDC4	0.5537195	-0.0379411	0.5888036	-0.0954809
ENST00000458381	NOSTRIN	-2.8419222	-3.6188322	-2.8897163	-3.7853008
ENST00000367836	GPR161	-2.7751448	-3.3969932	-2.8115863	-3.5279607
ENST00000401533	GAREM2	-0.9335773	-1.6188514	-0.9149314	-1.6907029
ENST00000282469	SPEF2	-1.9650502	-2.6517648	-1.9716247	-2.7509884
ENST00000515655	ABCG2	-3.0670758	-3.7848838	-3.1258468	-3.890682
ENST00000572195	OVCA2	-3.0710892	-3.6891809	-3.1505887	-3.8901756
ENST00000380636	TMSB4X	4.0444721	3.4561514	3.9138958	3.170853
ENST00000360871	PRDM10	-2.5172505	-3.2072439	-2.5433243	-3.3309178

NM_001160005	NRG1	-2.1932874	-2.8350678	-2.2053963	-2.9412828
ENST00000309311	EEF2	4.7090579	4.0526542	4.6945998	3.9738263
ENST00000536441	SESN3	-1.603904	-3.0441539	-1.5983718	-3.1616978
HBMT00000732734	CATG00000040494.1	-2.651081	-3.2500012	-2.7036957	-3.4263345
ENST00000475551	CCM2	-0.5743971	-2.2610413	-1.1013621	-2.9505874
ENST00000299736	CENPV	-1.2235855	-1.9075042	-1.2111576	-1.9852003
ENST00000307216	TMEM87A	-1.8161436	-2.4998129	-1.8199395	-2.5920189
ENST00000345724	MALT1	-2.5142974	-3.1632156	-2.5352736	-3.2701545
ENST00000644112	TIMM8A	-2.0117018	-2.654035	-2.0207631	-2.7499924
ENST00000268483	TXNL4B	-2.4148436	-3.2938667	-2.4361241	-3.5046498
NM_001243476	STARD13	-2.7012798	-3.6041696	-2.7344131	-3.6986355
ENST00000332598	TMED9	6.7893339	5.9649012	6.7106608	5.9451319
ENST00000457167	DNAJC7	-2.4664353	-3.1265723	-2.5808599	-3.4279481
ENST00000380629	BNIP3L	-2.7627017	-3.6724908	-2.8335398	-3.8631763
ENST00000426059	FN1	-0.099738	-0.7151612	-0.0697761	-0.7717862
ENST00000391851	PRMT1	-1.8765279	-2.6789949	-1.8951871	-2.8203553
ENST00000370336	DDX43	-1.3674117	-2.4327338	-1.3580713	-2.522129
ENST00000033079	FAM13B	-0.3247068	-1.2868249	-0.2978855	-1.3531968
ENST00000398955	MGARP	3.3860617	2.2344255	3.4231738	2.1816214
ENST00000432096	YAE1D1	0.27432	-0.5826605	0.30489	-0.6419481
ENST00000373467	HPCA	-2.1144113	-2.706719	-2.1372206	-2.8071632
ENST00000382699	EVA1C	-2.1848329	-3.1787991	-2.1961745	-3.2998595
ENST00000226317	CXCL6	-1.7559389	-3.167338	-1.7562824	-3.2883581
ENST00000312216	MCTP1	1.9477134	1.061133	1.9951065	1.0092579
ENST00000369099	C1orf54	-2.3065759	-2.9069838	-2.3628997	-3.0292813
ENST00000249377	LRRC17	-0.6566874	-1.4830291	-0.6714076	-1.6370698
ENST00000265840	ELMOD1	-0.8030291	-2.1035335	-0.7894748	-2.2982231
ENST00000166534	P4HA2	0.4585591	-0.1302627	0.4636464	-0.2596062
ENST00000598441	SNRNP70	1.7392788	0.9124595	1.7714416	0.8464004
ENST00000589786	KCTD15	-1.9644803	-2.7191054	-1.9664243	-2.8188427
ENST00000456764	IGFBP2	-1.106931	-1.7724423	-1.1157761	-1.8710176
ENST00000395511	DHRS7B	-2.1505928	-2.7384549	-2.1622908	-2.8376949

ENST00000585474	TXNL4A	-1.7429419	-2.3901351	-1.7443562	-2.4779846
ENST00000620139	MREG	-2.1859007	-2.8037822	-2.1996483	-2.9045436
ENST00000255681	MACROD1	-0.0160998	-1.931451	-0.4921118	-2.5248848
ENST00000289816	ZNF276	-0.2455508	-1.187784	-0.221486	-1.259344
ENST00000392593	PLD4	-0.2459242	-0.8620433	-0.2195109	-0.9209367
ENST00000266085	TIMP3	-0.6078426	-1.9036402	-0.5793933	-1.9787609
ENST00000397332	MYCL	-0.4121559	-1.0276974	-0.4192005	-1.1505595
ENST00000539261	MGP	3.1391286	2.017831	3.1573157	1.938365
ENST00000428358	PPAN-P2RY11	1.1036654	0.4469646	1.136939	0.3823976
ENST00000620222	ZNF117	1.364109	0.6234005	1.4127284	0.5726847
ENST00000393958	PC	-1.1042629	-2.3685444	-1.1374341	-2.5792735
ENST00000393960	PC	0.5793392	-0.7506505	0.5903631	-0.83978
uc011mhe.2	ASMTL	-2.3269017	-3.0691146	-2.563174	-3.405312
ENST00000537691	CPNE6	-2.83101	-3.4320109	-2.9890414	-3.7048594
NM_016148	SHANK1	-2.8097797	-3.4259969	-2.8754573	-3.6720129
ENST00000372524	ZNF503	-0.377522	-1.0717311	-0.3481916	-1.1388849
ENST00000262210	CSPP1	-1.5797829	-2.2158944	-1.5716961	-2.2997101
NM_001354714	THRB	-2.6567862	-3.3371655	-2.6919689	-3.4710373
ENST00000379215	ECHDC3	-1.9560052	-2.769285	-1.9729626	-2.8720028
ENST00000248668	LRFN1	-2.7080724	-3.3381933	-2.7402326	-3.4099376
ENST00000591340	ZNF529	-2.5569627	-3.1814179	-2.7286472	-3.545144
ENST00000369902	SUFU	-2.3276892	-3.0575645	-2.3525672	-3.166994
ENST00000375151	TSR2	-1.7370669	-2.3302655	-1.7354317	-2.4143661
ENST00000511507	GUCY1A3	-2.622862	-3.4716456	-2.6614195	-3.5965432
ENST00000378458	FAM107B	-1.7204329	-2.3301839	-1.7195177	-2.4203798
ENST00000329841	ALDH1A3	-2.2903295	-4.3907798	-2.3077017	-4.6065844
ENST00000282670	TCTEX1D1	-0.212052	-1.191629	-0.18053	-1.2567996
ENST00000538261	CLDN7	-1.8762684	-2.7063477	-1.8798147	-2.8044058
NM_001258453	FBXO18	-2.150903	-2.8356614	-2.1808909	-3.0086408
ENST00000299964	NNMT	-2.1426233	-2.9468407	-2.1527745	-3.0548247
ENST00000263735	EPCAM	-2.5780775	-3.3903233	-2.6059873	-3.5223618
ENST00000523403	TXNRD3	-2.0483181	-2.8632256	-2.0741543	-2.9837833
ENST00000276925	CDKN2B	-0.8087052	-1.5101302	-0.7857423	-1.5804681

ENST00000328654	ZNF26	-1.2237098	-1.8546345	-1.2117603	-1.9290385
ENST00000381222	ZBED1	-2.3728594	-3.2301745	-2.5741412	-3.6118128
ENST00000358065	SETMAR	-1.4790754	-2.1489672	-1.466899	-2.2310159
ENST00000373970	DKK1	-0.5193777	-1.1943909	-0.5026141	-1.2856485
ENST00000341923	RABEP1	-2.0950911	-2.7220251	-2.1049049	-2.8261043
ENST00000377934	CXorf36	-0.0969389	-0.7929824	-0.0669418	-0.8528479
ENST00000455858	FBXW8	-2.3669357	-3.1942328	-2.6473719	-3.3987277
ENST00000360521	PFKFB3	3.2603514	2.6255644	3.2848923	2.5668105
ENST00000349999	HFE	-2.7569956	-3.4126749	-2.7935036	-3.5453327
ENST00000374126	ZNF618	-2.4244364	-3.0799397	-2.4431667	-3.1906127
ENST00000253110	C19orf66	-2.5747186	-3.4674181	-2.6225524	-3.704583
ENST00000447215	IMMP2L	-2.4438968	-3.0391756	-2.4628541	-3.1588759
ENST00000361603	COL4A5	-2.699653	-3.3234152	-2.8267768	-3.4450312
ENST00000313860	LIMCH1	-2.2269805	-2.8864604	-2.2700822	-3.016695
ENST00000295971	RBM47	-2.1743709	-2.8207613	-2.2486026	-3.1512494
ENST00000521662	ENY2	-1.1402401	-1.897504	-1.2421072	-2.0318801
ENST00000359798	ZNF808	-2.5917928	-3.4457204	-2.6472285	-3.5453816
ENST00000262375	DNAJA3	-2.4692817	-3.3053399	-2.5192276	-3.4415581
ENST00000295461	NIPAL1	-2.7651675	-3.3579518	-2.8068603	-3.4798709
ENST00000444983	CCNE1	-2.3829111	-3.4206851	-2.410304	-3.6796083
ENST00000268797	GFOD2	-0.8056353	-1.4068201	-0.7848706	-1.4755099
ENST00000331817	KRT7	-0.1525098	-0.7506961	-0.2244763	-1.0562699
ENST00000375916	APOM	-2.4108798	-3.0248469	-2.5649199	-3.453478
ENST00000370404	CXorf40B	-2.2405734	-2.8732565	-2.260275	-2.9798267
ENST00000424155	TMED3	-1.3397236	-1.9305443	-1.3673547	-2.0955662
ENST00000555004	C14orf132	-0.6451509	-1.5317112	-0.6542268	-1.738123
ENST00000582524	NAT9	-2.0063436	-2.7002967	-2.1050137	-2.9387857
ENST00000540691	KXD1	-2.8960012	-3.5581896	-2.9393942	-3.6602293
ENCT00000383043	CATG00000087855<br>.1	-2.1871826	-2.8720825	-2.5583922	-3.2695702
ENST00000344204	TRIO	-2.225484	-3.2170175	-2.2352768	-3.3721008
ENST00000414773	CLGN	-2.2979681	-3.5718546	-2.336176	-3.741245
ENST00000335119	CCIN	-1.9505716	-2.5622282	-1.956335	-2.6558429

ENST00000319286	ZNF572	-1.153828	-2.3806548	-1.3522778	-2.5171266
ENST00000378412	MMEL1	-2.9182362	-3.5136518	-2.9710369	-3.6635208
ENST00000504761	TCOF1	-0.6053543	-1.2010644	-0.6230333	-1.3347017
NM_001304793	SAYSD1	-2.462885	-3.8658711	-2.4825049	-4.027406
ENST00000375835	SHC3	-1.3680707	-2.0049133	-1.3581724	-2.0809545
NM_001321733	EXOC6B	-2.6981643	-3.3052927	-2.7365727	-3.4322904
ENST00000359042	C17orf80	-1.5607926	-2.1811819	-1.5638973	-2.2856466
ENST00000392748	VWA5A	-2.7472193	-3.3538101	-2.8194229	-3.4970723
ENST00000405413	HPSE	-1.2492141	-2.0150513	-1.2357907	-2.094078
ENST00000331758	SS18L1	-1.6732684	-2.2728403	-1.6691189	-2.35808
ENST00000512648	SPOCK3	0.0603751	-0.6004987	-0.0080221	-0.7683009
ENST00000361991	CSNK1G3	-2.6455549	-3.3525189	-2.6766823	-3.4736365
uc010gwx.3	APOL1	-1.3978336	-2.0013223	-1.4015378	-2.0804578
ENST00000262593	DOK5	-0.779261	-1.9111798	-0.7682504	-1.9887656
ENST00000434463	HSDL1	-2.3537317	-2.9565618	-2.3759729	-3.0770517
ENST00000372172	HECTD3	-2.2883956	-2.9830521	-2.3288105	-3.1015853
ENST00000542301	P2RX2	-1.7182524	-2.335374	-1.7184689	-2.4221736
ENST00000552049	GRASP	-1.9686144	-2.7858665	-2.043919	-3.0037537
ENST00000398090	SLC7A2	-2.9537674	-3.8296143	-3.0019597	-3.9493922
ENST00000340941	MCCC2	-2.3090841	-3.3085819	-2.3248452	-3.4356189
ENST00000286627	KCNMA1	-0.8164195	-1.4145915	-0.8006161	-1.5025002
NM_001329996	PRR15	-2.2486898	-2.8569387	-2.2653537	-2.9621767
ENST00000367913	HSD17B7	-1.457706	-2.1937321	-1.4674844	-2.3375364
NM_001305086	NAT9	-2.2884522	-3.1717459	-2.440775	-3.4554961
ENST00000352251	PROSER1	-2.5965007	-3.2756742	-2.6233427	-3.3981974
ENST00000506866	SETD7	-2.5719631	-3.9402779	-2.5980734	-4.1051277
ENST00000397007	NUP98	-2.7595995	-3.5522273	-2.7956475	-3.6693402
ENST00000237889	NDUFB3	-1.669287	-2.2959864	-1.7335843	-2.4086987
ENST00000230449	EXOC2	-0.7822582	-1.4990286	-0.8305347	-1.7263542
ENST00000368211	MRPL24	-2.0891077	-2.7768879	-2.1056593	-2.9238325
ENST00000239938	EGR1	1.2300121	0.2947924	1.274057	0.2400345
ENST00000377834	HES2	-2.4601138	-3.2405228	-2.7114433	-3.767929
ENST00000486442	KLHL29	-2.9666404	-3.5824903	-3.2270227	-4.1184259

ENST00000358029	STX16	-2.3136548	-2.9304473	-2.3259591	-3.0337741
ENST00000373313	MAFB	-0.236158	-1.2814495	-0.2168597	-1.3578699
ENST00000421109	NR2F2	-1.4443407	-2.045415	-1.4481967	-2.1235367
ENST00000257659	GTPBP10	-2.0560322	-2.7942666	-2.0700874	-2.8949425
ENST00000588852	SAFB	-2.2686548	-3.3575441	-2.2927032	-3.4472113
ENST00000390645	ARL4C	-0.1174618	-0.7169005	-0.0891866	-0.779075
ENST00000397356	HAGH	-0.5251471	-1.1377437	-1.1765409	-1.9176584
ENST00000223357	AEBP1	-1.3343709	-2.0948314	-1.331025	-2.1754862
ENST00000367737	METTL13	-0.9388212	-1.5565906	-0.9188612	-1.6304631
ENST00000307407	CXCL8	-1.8026839	-2.4233627	-1.8024118	-2.5121263
ENST00000370351	SLC17A9	-1.2170973	-1.8078819	-1.2026512	-1.8835811
ENST00000394775	CCDC121	-2.6947662	-3.3276672	-2.7268389	-3.4649399
ENST00000378387	ARHGEF39	-3.0825079	-3.7942131	-3.2434889	-3.8976162
ENST00000368872	PI4KB	-2.9065985	-3.8537725	-2.953972	-3.9883956
ENST00000603798	SMAGP	-1.7833161	-2.4860578	-1.7851631	-2.580241
ENST00000372324	B4GALT2	-0.272273	-0.8776975	-0.2486693	-0.9421378
ENST00000560104	BLID	0.4641225	-0.1220271	0.4979597	-0.1826373
ENST00000379538	SNCAIP	-2.2338259	-2.8721046	-2.2597199	-2.9779387
ENST00000552864	LIN7A	-0.7483597	-1.6102076	-0.7231979	-1.6803371
ENST00000378526	LIG3	-0.2347022	-0.8354189	-0.2663995	-0.949977
NM_001135936	POSTN	-1.4209951	-2.5101605	-1.4143837	-2.6039253
ENST00000317178	ZNF776	-1.5892613	-2.2839246	-1.5814332	-2.37109
NM_001324281	CARD11	-2.2661935	-3.1430856	-2.2854043	-3.2667453
NM_001326358	ADAMTS18	-0.3636782	-1.2671388	-0.3395106	-1.3327493
ENST00000372108	GTF3C5	-1.795813	-2.3930144	-1.9636007	-2.8808155
ENST00000261024	SLC40A1	-0.9024386	-1.6630208	-0.8812136	-1.7377674
ENST00000263736	SRBD1	-1.7888756	-2.4494819	-1.7894883	-2.5408527
ENST00000300087	DCTN5	-1.5762015	-2.172099	-1.5693156	-2.2562421
ENST00000219368	FA2H	-2.9457005	-3.567999	-3.0093941	-3.851162
ENST00000309957	NAXD	-2.1830567	-2.9915223	-2.3765464	-3.4633783
ENST00000346691	ATRIP	-1.5936379	-2.187151	-1.5879899	-2.3010586
ENST00000440177	ERF	-1.9140787	-2.5293541	-1.9855604	-2.690517
ENST00000372228	POMT1	-2.5226206	-3.3953812	-2.6391103	-3.7372749

ENST00000381571	COMMD8	-1.449242	-2.0678453	-1.4387546	-2.1467778
ENST00000373464	ZBTB43	-2.4922147	-3.2489983	-2.5158041	-3.3649559
ENST00000009180	CD9	5.5799494	4.9616114	5.5813442	4.9359918
ENST00000357328	ZNF248	-0.9982843	-2.1830727	-0.9853304	-2.2841182
ENST00000329913	GDF3	-1.0282438	-2.4100168	-1.0954426	-2.7316668
ENST00000504248	MAN2B2	-2.8555634	-3.4441226	-2.9181669	-3.6280053
ENST00000458412	HYPK	-1.8995662	-2.6667444	-1.9903695	-2.8579718
ENST00000378090	C12orf73	-2.5361023	-3.2750484	-2.5630902	-3.3913098
ENST00000539247	ARMCX6	-2.6438481	-3.2530778	-2.6735224	-3.3675906
ENST00000426338	PQLC2L	-2.789571	-3.7456828	-2.8344625	-4.0128584
ENST00000407217	CRELD2	-0.2402483	-0.8677487	-0.2149037	-0.9419071
ENST00000590959	SPHK1	-2.2805466	-3.2295288	-2.2926411	-3.3522151
ENST00000374690	AR	-0.492897	-1.5187076	-0.4666733	-1.5917172
ENST00000539055	ARL1	-2.6565839	-3.6017015	-2.6868773	-3.701662
ENST00000456523	FGF5	-0.6360375	-1.2696327	-0.6124029	-1.3386814
ENST00000376021	TEX30	-2.6016097	-3.1948323	-2.6788972	-3.3474689
ENST00000337540	ZFYVE27	-2.7449137	-3.7198199	-2.9301815	-4.0493164
ENST00000393409	PLXNA1	-1.8244794	-2.5657273	-1.8286462	-2.6618188
ENST00000375413	BPIFA1	-2.5403966	-3.1519772	-2.6763261	-3.4876802
ENST00000427926	CLTCL1	-1.1154365	-1.7666956	-1.112102	-1.8533023
ENST00000264827	HOOK2	-1.4919536	-2.1868328	-1.5243973	-2.3891643
ENST00000303657	RFX2	-1.7270789	-2.5900171	-1.7248854	-2.6898124
ENST00000399988	MAGEB16	-2.676702	-3.2697249	-2.7906201	-3.4489132
ENST00000336292	SYNM	-2.3771879	-3.1600403	-2.4069243	-3.2713203
ENST00000369808	DNASE1L1	-2.1997002	-2.890756	-2.237724	-3.0878811
ENCT00000146661	CATG00000024467<br>.1	0.5276793	-0.7622671	0.0932031	-1.0761912
ENST00000333039	NPTXR	-2.4625765	-3.0939204	-2.4821192	-3.2197647
ENST00000282849	ADAMTS18	-0.4836284	-1.1941813	-0.4581631	-1.2608985
ENST00000245817	TNFSF9	-2.1312293	-2.8339715	-2.1619561	-2.9416069
ENST00000391759	TFPT	-2.2105473	-2.9957093	-2.2274375	-3.1485798
ENST00000378785	ATAD3C	-1.923452	-2.5734722	-1.9335219	-2.6951643
ENST00000402394	CGREF1	1.2565186	0.6306995	1.301174	0.5814548

FTMT22000002399	CATG00000075756.1	2.2677543	1.2989676	0.5285222	-0.4603326
ENST00000371126	DCAF12L1	1.5966327	0.7836883	1.6254232	0.7052803
ENST00000366987	ATF3	-2.3038002	-3.028438	-2.3796579	-3.1617513
ENST00000611975	SEMA4F	-2.5206324	-3.9588153	-2.5655466	-4.2054208
ENST00000537328	TMEM258	-2.6590402	-3.5098551	-2.7205915	-3.667423
ENST00000333137	SMTN	-2.2059798	-2.8394122	-2.2174704	-2.946772
ENST00000335255	FOXK2	-2.6540299	-3.6700208	-2.689306	-3.8499741
ENST00000340160	TSPAN18	0.2520122	-0.6085766	0.2856535	-0.6695048
ENST00000369875	CNNM2	-2.546566	-3.4980698	-2.5707936	-3.6325778
ENST00000563902	KIAA0895L	-2.7370562	-3.5192236	-2.7762687	-3.6358148
ENST00000582743	SLC16A3	2.4191791	1.654398	2.4612308	1.5898628
ENST00000258187	NDRG4	1.952793	1.0465658	2.0020371	0.9946265
ENST00000262327	LIG3	-1.5923677	-2.2301614	-1.6124992	-2.3419971
ENST00000420893	FAM13B	-0.2810252	-0.9513151	-0.4125489	-1.2748462
ENST00000645506	MSH2	-1.2995223	-1.8898746	-1.6340372	-2.5983853
ENST00000441698	MSRA	-1.7894766	-2.3753824	-1.7967162	-2.479486
NM_001267810	EIF6	-2.5979257	-3.2181963	-2.7358877	-3.3904159
ENST00000510027	SPINK1	-0.2358099	-3.1682372	-0.6208205	-3.5076422
ENST00000476858	APTX	-2.6063248	-3.2386207	-2.6501261	-3.3583999
ENST00000371600	NPDC1	-2.0067251	-2.7321512	-2.0531328	-2.8309874
ENST00000331268	MORF4L1	-2.4263069	-3.0816281	-2.4494833	-3.1955299
ENST00000358432	EPHA2	0.4510583	-0.1524861	0.4688889	-0.2605643
NM_001142598	P4HA2	1.7265111	1.0721227	1.7733965	1.00566
ENST00000273666	STXBP5L	-2.2920797	-2.9317641	-2.3483113	-3.1464694
ENST00000293443	FAM171A2	0.0378794	-0.5600803	0.0614883	-0.6253334
ENST00000396043	AR	-2.4779489	-3.1408495	-2.8310332	-3.9054977
ENST00000446023	MGAT1	-2.6077975	-3.2696986	-2.7241509	-3.4579383
ENST00000299565	CHRNA5	-1.8288241	-2.6313441	-1.8329858	-2.7293165
ENST00000401867	P4HA2	-1.4235723	-2.051617	-1.4114158	-2.1300237
ENST00000641879	OR11G2	-2.7016264	-3.3455097	-2.7376663	-3.5183409
ENST00000530028	EID1	-2.1708976	-2.8985517	-2.2053264	-3.0070048
ENST00000355754	GBP4	-1.3487777	-2.0303675	-1.3392633	-2.1154802

ENST00000282903	PLOD2	1.2751021	0.5889359	1.3078844	0.5154866
ENST00000402744	CPE	-0.8835072	-2.2124894	-0.8595962	-2.3225015

Supplementary Table 4. shIGFBP5 vs shNC (hypo-down)

		Expresstion		Methyation	
Transcript ID	Gene Symbol	shIGFBP5-group(mean)	shNC-group(mean)	shIGFBP5-group(mean)	shNC-group(mean)
ENST00000375371	TAS1R2	3.0931948	3.9099593	2.8420714	3.5204927
ENST00000335895	BTF3	3.4042546	5.246247	2.2431476	3.4980627
ENST00000292586	C5orf45	-2.8397252	-2.0237383	-3.0016833	-2.2200432
ENST00000381980	C4orf19	-3.1807767	-2.2556281	-3.2426321	-2.3436234
NM_001194991	MEPCE	3.1262853	3.9456154	2.357075	3.3947208
NM_001350237	PSRC1	-3.0461584	-2.2898019	-3.3419608	-2.5989278
ENST00000545507	BSG	4.5298139	7.2400404	3.6505997	6.7543782
ENST00000338101	NOS1	0.0095872	0.908405	-1.1513116	-0.2400557
ENST00000467467	WWTR1	-1.5425841	0.1504614	-2.7728616	-1.4087936
ENST00000334696	ENTPD5	-2.7254695	-2.1226276	-2.8718429	-2.2711487
ENST00000228567	SYT10	1.6368103	2.5472094	1.6531079	2.4705102
NM_001278232	ZNF189	-3.3903383	-2.7142398	-3.8974478	-3.0362743
ENST00000419880	UBE2W	-3.7128462	-2.9398783	-3.8554336	-3.1113903
ENST00000614189	PCGF5	-2.5373671	-1.4936312	-2.5985905	-1.6099765
NM_001145134	CHKB-CPT1B	-0.7623121	-0.0784645	-0.8582758	-0.2107735
ENST00000562971	ZNF267	7.9141599	9.251729	7.1948394	8.7894271
ENST00000287907	HTR5A	-1.7564119	-1.0264981	-2.5577385	-1.7567864
ENST00000392011	SH3BP4	2.9434819	4.5421872	1.8898753	3.8094749
ENST00000247833	RAB3IP	-2.3452015	-1.7060608	-2.3727394	-1.78104
ENST00000393240	SYT1	-2.5655329	-1.9262238	-3.4867165	-2.8256512
ENST00000397624	MEIS2	-3.3894091	-2.6425461	-3.46315	-2.7407496
ENST00000375482	FGD3	-3.6648137	-2.2772715	-4.8361823	-2.6718237
ENST00000356462	NEDD4L	-1.0336164	-0.2111925	-1.126726	-0.355234
ENST00000288050	PDPR	-3.5687818	-2.7016345	-3.6404622	-2.8138122
ENST00000312989	PPP1CA	-3.3905111	-2.7026483	-3.4667283	-2.8265042
ENST00000340957	FAM131A	-2.9897482	-2.2879321	-3.1870315	-2.5548576
ENST00000359610	OR6K2	3.1335185	5.0446719	2.8846365	4.5849389

ENST00000595944	ZSCAN18	3.0507249	3.744609	2.8329717	3.4692095
ENST00000566005	NOXO1	5.561096	7.9302931	4.8007966	7.472238
ENST00000376162	ITGBL1	-3.0448834	-2.3250384	-3.0951097	-2.4209995
ENST00000317459	TRPT1	3.0654852	4.1338855	2.3202402	2.9868651
ENST00000367603	HIVEP2	-2.344542	-1.632599	-2.763376	-1.9967944
NM_001303242	PRUNE	-3.5079829	-2.7888291	-3.6997561	-2.9349551
NM_001346544	MRAP2	-2.4271079	-1.7779632	-3.1283392	-2.323294
ENST00000552561	TMEM106C	1.3485509	4.1506982	0.8316187	3.5264281
ENST00000216177	PNPLA5	-3.2126565	-2.6125442	-3.5924127	-3.006592
NM_001194997	CARS	-1.3488744	-0.7407873	-2.0809612	-1.431285
ENST00000328666	SIRT7	-3.5712016	-2.8690176	-4.0034677	-3.1639765
ENST00000427262	SEMA3E	1.4800249	2.6648591	1.3586593	2.3235405
ENST00000444376	HNRNPU	2.7587586	4.1657885	1.1540354	2.8837928
ENST00000380206	IFNA2	-4.0772372	-3.0512775	-4.2965005	-3.2118329
ENST00000436609	ALG13	0.6033555	1.4255268	-0.0878614	0.736562
ENST00000523261	NKX3-1	-3.5257953	-2.5505481	-3.9411101	-2.7598319
ENST00000265626	PMEPA1	-0.9744127	1.359916	-1.1066983	1.2591368
ENST00000557471	PAPOLA	-0.4544585	0.5451503	-1.7518701	-0.6644604
ENST00000393354	B4GALNT2	2.7861754	3.4807997	2.5271093	3.1575447
ENST00000394202	CNN3	-2.4450693	-1.7320419	-2.6835259	-2.0009025
ENST00000417572	SEC22C	2.152141	2.7591113	1.6631635	2.2554748
ENST00000372987	CCND3	2.7334821	3.365093	1.5110537	2.2891335
ENST00000419165	TMSB15B	3.0538385	3.7985187	1.804537	2.4148731
ENST00000409993	DPAGT1	1.6551487	2.4235659	0.5569048	1.2412597
ENST00000614686	PFKFB1	0.2182305	1.0906163	0.2510871	1.0328241
NM_001329517	ZNF420	-3.4990536	-2.7341438	-3.6454978	-2.9107276
ENST00000348495	SSBP4	-2.4642784	-1.6989057	-4.0177181	-2.8019764
ENST00000371210	FGGY	-3.454693	-2.5817924	-3.8157882	-2.819733
ENST00000606865	ACYP2	-3.5390376	-2.8123417	-3.6387686	-2.9183709
ENST00000396442	OCLN	-3.4139505	-2.5690662	-3.4906218	-2.6650819
ENST00000347900	KIRREL2	0.131332	0.9688458	-1.2237495	-0.3357214
ENST00000289373	TMSB15A	-3.1153622	-2.4551064	-3.1706132	-2.5436508

ENCT00000200335	CATG00000038184.1	-2.3086849	-1.6225277	-2.3227782	-1.6928744
NM_001320823	WDR54	1.2231148	1.9051825	0.8053325	1.4529363
ENST00000498623	ARPC4	-3.1501671	-2.3665268	-3.4865521	-2.7013373
ENST00000496384	BRAF	-1.0005213	0.0667665	-2.3766175	-0.8057943
ENST00000395068	GRINA	-3.2113576	-2.5854613	-3.5599116	-2.8028567
ENST00000430267	GPR35	-1.0049705	-0.3594268	-1.747608	-1.1615762
ENST00000526995	TRAF6	1.3862682	2.1328428	0.1157386	0.8206216
ENST00000441248	CXorf40A	-0.4786716	0.5688715	-1.95537	-0.711045
ENST00000264716	FOSL2	0.2207939	1.7107804	-0.0700857	0.7707843
ENST00000310624	NEFH	-3.4815426	-2.5051868	-3.5644297	-2.5979436
ENST00000570836	ALOX15	-2.7248991	-1.558913	-2.8267268	-1.6651723
ENST00000426130	CYB561A3	-3.5375834	-2.7482772	-3.6210585	-2.8556718
ENST00000345127	PRICKLE1	2.7480762	5.3589452	1.7012339	4.780816
NM_001349978	RASGRP3	-2.3915997	-1.6384354	-2.6161585	-1.8608662
ENST00000590202	ZNF519	3.2488407	4.3165476	2.3737369	3.4704253
ENST00000348931	ZPBP2	-0.4226969	0.8068065	-1.1689099	-0.1440604
ENST00000262625	KIRREL2	0.8160479	1.7522003	-0.6227215	0.4101657
NM_001079522	KTN1	3.7907072	5.3913301	3.2030657	4.8453201
MICT00000303414	CATG00000083839.1	-0.7767754	0.0854256	-1.6797427	-0.9839979
ENST00000356244	RANGAP1	-3.8667392	-2.9389944	-4.3796768	-3.1925758
ENST00000346916	BSG	4.1402257	6.8295537	3.2214529	6.3074295
ENST00000375547	PROZ	2.758624	5.067257	1.3594898	3.3502695
ENST00000215885	PLA2G3	-3.2805078	-2.2369191	-3.5518193	-2.4761491
NM_001350253	TRMT5	-0.8216354	0.9266147	-1.4178605	-0.4371393
ENST00000326866	ZFP42	0.7836141	2.2704661	-0.8606107	0.5366732
ENST00000439399	SNAP91	-3.643003	-2.5475061	-4.0829971	-2.7050835
ENST00000547905	RACGAP1	-0.3308801	0.3588095	-2.2771068	-1.6861987
ENST00000356120	NOXO1	2.5148457	3.2565525	1.2774591	2.0537902
ENST00000308831	RHOD	-3.3856977	-2.6921181	-3.5667814	-2.8268615
ENST00000505606	ZNF131	6.0726406	7.9944556	5.4282983	7.519135
ENST00000360202	KIRREL2	2.2169715	3.0137988	1.4089666	2.0927754

ENST00000424149	CYHR1	2.2952516	3.0697765	1.2835498	1.9352858
ENST00000272972	ANKMY1	0.106312	0.9052795	-0.2228173	0.4308081
NM_001322076	ZSCAN5A	-0.6460825	0.0690882	-0.6684384	0.0039187
ENST00000299204	BAG5	-2.9576732	-2.3371074	-3.0127657	-2.4270475
ENST00000283296	ADGRF5	-1.0674487	-0.0790427	-1.052369	-0.1387313
ENST00000640608	GOSR2	-3.6792982	-3.0273781	-4.0014838	-3.3204336
ENST00000610540	CAB39L	-3.2233969	-2.3292608	-3.3529047	-2.4716587
ENST00000084798	CA11	-0.9254981	0.0590385	-1.5670753	-0.3317929
ENST00000252595	SLC27A1	0.7905881	1.3834024	0.2115324	0.8181226
ENST00000540839	FAM65A	3.7488442	4.9650267	2.3888792	3.2621457
ENST00000316793	OXTR	-2.8568505	-1.9316561	-3.3393443	-2.2252378
ENST00000301464	IGFBP6	-1.1606467	-0.4363187	-1.2539743	-0.5726865
ENST00000613060	SLC6A6	-3.3178808	-2.6701118	-3.3892593	-2.7670988
ENST00000397981	MAP2K7	3.693825	4.3531126	2.9987722	3.6463301
ENST00000458591	SCFD1	0.493905	1.8791196	0.0515482	1.2813762
ENST00000355029	NET1	-1.8666851	-1.2314227	-3.4954253	-2.6901467
MICT00000219633	CATG00000053533.1	-2.5876421	-1.9876904	-3.119641	-2.3986924
ENST00000295228	INHBB	-3.3190367	-2.5594459	-3.7542031	-2.6578028
ENST00000408011	KCTD1	-1.2538678	0.3847611	-2.0876567	-0.9119227
ENST00000636466	GABRB3	-2.8715896	-2.2681928	-3.5835006	-2.898734
ENST00000495479	N4BP2L1	-2.3916279	-1.6705319	-2.409626	-1.7435811
NM_001318473	ZFP1	-3.363436	-2.6883503	-3.4504907	-2.8085701
NM_001346469	ANO10	-3.304215	-2.5450393	-3.3811436	-2.6385774
ENST00000409212	SH3BP4	3.3347441	4.486532	1.7980382	3.2721225
HBMT00000396921	CATG00000018005.1	0.8448676	1.7826255	-0.2409599	0.6784531
NM_001309444	SPARC	-2.4924415	-1.7621397	-3.322564	-2.5540458
ENST00000272521	TMEM177	-1.9328795	-0.1844019	-2.5660405	-1.3945393
ENST00000437682	ADAM32	-2.4312102	-1.7539704	-3.0918633	-2.4514379
ENST00000433779	SUV39H2	-2.4801159	-1.752975	-2.7624691	-1.9613221
ENST00000458031	ABHD14A	3.5574736	5.4660509	3.0506977	4.0135586
ENST00000323090	SULT2B1	6.4071084	7.6852171	5.5688696	7.0142656

ENST00000618760	NOS1	3.9450454	4.6873738	2.8153543	3.6920312
ENST00000368551	FUOM	-0.286221	1.3428553	-1.2473321	-0.2016652
ENST00000600404	ZSCAN18	1.9559495	2.6378962	1.6932297	2.2951919
ENST00000397280	NOXO1	5.5764919	7.2412674	4.4811676	6.5640083
ENST00000451287	PPM1N	-3.5946555	-2.8204276	-3.7817834	-3.0304745
ENST00000290705	MT1A	-3.2395614	-2.5547749	-3.5021479	-2.6992444
ENST00000313516	THAP5	-3.1721256	-2.3857159	-3.231267	-2.4789264
NM_001321917	ZFP82	-2.3598581	-1.6953003	-3.5047575	-2.8823204
ENST00000416589	TEX264	-3.1215172	-2.2255272	-3.4552432	-2.4523594
ENST00000533357	MPZ	-1.4899603	-0.4738336	-1.5314515	-0.534289
ENST00000317987	FDCSP	-3.4657256	-2.737609	-3.6058315	-2.8399699
ENST00000537882	NDRG1	0.216257	2.9012097	-1.0919822	1.1977623
ENST00000595796	SLC38A5	4.9598658	6.4220646	4.0412115	5.7454061
ENST00000333535	SCN5A	-3.1060548	-2.4855323	-3.194752	-2.5792078
ENST00000375630	ATP11A	-3.1287401	-2.4959423	-3.1930591	-2.586666
NM_001287013	GLIPR2	4.881597	6.8312687	4.3547349	6.3897161
ENST00000549035	POC1B	0.2535131	1.1153539	-1.2170068	-0.3213471
ENST00000327979	FAM65C	-1.1827952	-0.3405523	-1.3626914	-0.6105654
ENST00000256178	LYVE1	-2.4548884	-1.5825391	-2.5001205	-1.6908621
ENST00000521848	NKX2-5	1.2529088	1.8796963	0.6223591	1.222728
NM_001353163	ZMYM2	-2.445942	-1.770814	-2.6755003	-2.0209981
NM_001300806	MDFI	-2.6153097	-1.8438119	-2.7465763	-1.975071
ENST00000315614	NEURL4	-3.615973	-2.5860781	-3.8588917	-2.9291071
ENST00000331563	PKP3	-1.9551873	-1.0572615	-2.1692556	-1.2810431
ENST00000356433	DLL3	0.225637	1.1853334	-0.9387955	-0.0198041
NM_001353374	ZNF879	-2.2051482	-1.6133582	-3.9807012	-2.9645981
uc010ogu.1	TMEM39B	3.040357	3.7012467	2.5641792	3.1628423
ENST00000359499	KRT78	4.1078932	5.3131868	3.8316389	4.8734933
ENST00000526620	AMICA1	0.0616127	1.2397775	-0.330116	0.9686104
ENST00000342783	PROZ	2.8676705	5.0987136	1.1753632	3.2030283
ENST00000503201	NR3C1	3.3851921	6.2031207	3.0134664	5.8631858
ENST00000592741	PRKCSH	0.5843723	3.0769905	-0.6022149	1.3531655
ENST00000436108	CHID1	2.9882195	4.9429853	1.9809491	3.4284421

ENST00000453097	SLC4A8	-3.5938237	-2.9538393	-3.7307977	-3.1098997
ENST00000409809	COL6A3	-2.5494384	-1.9200737	-2.6290909	-1.9984998
ENST00000621345	CASP7	-1.5700646	-0.8668752	-1.5827218	-0.9503869
ENST00000482567	PLSCR5	2.8015212	4.6517112	2.5586779	4.1904412
ENST00000540482	SLC12A9	2.8428545	5.3569073	1.2312047	3.6284289
ENST00000415287	SLC12A9	1.1208928	3.8872564	-0.6851308	1.937156
NM_001329758	FAM136A	1.5600783	3.4840051	0.4799666	2.0516957
ENST00000619700	GLIPR2	3.3480015	4.2481074	2.7496123	3.5990043
ENST00000265417	ADGRF5	-1.8410052	-1.1086459	-1.8732729	-1.1907509
ENST00000225648	HOXB6	-3.4997285	-2.557814	-3.5778291	-2.6531747
ENST00000244565	UNC5CL	-2.8840899	-2.1984202	-2.9410783	-2.2835118
ENST00000412052	ATRIP	-1.6279785	-0.7488016	-1.99721	-1.2493311
ENST00000300291	NUDT21	0.472108	1.6542728	0.3623112	1.3049578
ENST00000595658	MGC4771	-0.2310814	1.3598148	-1.3017463	0.2367524
ENST00000283269	CADPS	-1.3459708	-0.7106345	-3.3797956	-2.1892936
ENST00000647543	CTD-2643I7.4	-3.3036393	-2.7030596	-4.0581909	-3.1250153
ENST00000375840	STRADA	3.0497233	3.6591137	2.219597	2.862009
ENST00000604255	FXYD3	-2.3228369	-1.5685953	-2.3771001	-1.6649385
ENST00000355422	GFRA1	-1.5051931	-0.1789378	-2.0589366	-1.4073685
ENST00000340811	PKP2	0.0565946	0.9206192	0.0906776	0.8700658
ENST00000640135	SCN1B	-3.6583878	-2.8539988	-4.2009177	-3.3255308
ENST00000514254	DNAH10OS	1.659921	2.3291142	0.5909621	1.2557914
ENST00000445220	SHANK3	-3.06398	-2.2958813	-3.3233841	-2.5334359
ENST00000252594	NSUN5	-1.198684	0.957444	-2.2782189	-0.4935175
ENST00000442011	TGFBI	-2.780164	-2.0597224	-3.2204828	-2.4521496
ENST00000378279	AARD	-0.4544555	0.3527887	-0.7541257	-0.0294555
ENST00000403897	CAPN14	-3.099272	-2.4369293	-3.3154323	-2.5792214
ENST00000393704	MFAP3L	-0.2958826	0.2955213	-2.1884917	-1.3242358
ENST00000430930	TNS1	-2.2910621	-1.6725166	-2.8168535	-2.163447
ENST00000398576	LCP1	1.7129256	4.573797	-0.1355599	2.6249307
ENST00000388820	ADAMTS7	-3.3512635	-2.7160345	-3.4653351	-2.8304837
ENST00000518322	FAM92A1	-3.1562152	-2.297586	-3.5665605	-2.7701543

ENST00000201979	REM1	3.9545174	4.6559557	3.8119489	4.4080599
NM_001303029	PKP3	-2.2471364	-1.3134198	-2.45597	-1.5228238
ENST00000553521	SRSF5	1.6337162	3.2356315	0.5103481	1.8529698
ENST00000417454	GP6	0.517022	1.363326	0.2873735	1.1639876
ENST00000612929	BHLHE23	0.6796147	1.6283966	0.2243534	0.9830911
ENST00000307401	OR2AG1	-2.2796509	-1.5196181	-3.4777211	-2.5999678
MICT00000052695	CATG00000001316.1	0.6683125	2.7234553	-0.1552045	1.3043822
ENST00000380338	MLLT3	-3.4959293	-2.8676736	-3.5909741	-2.9727436
ENST00000369151	CACUL1	-3.2160022	-2.4258554	-3.2772623	-2.513943
ENST00000494076	TBRG4	-1.0334355	-0.4040824	-1.4851769	-0.8474846
ENST00000373826	RAB42	-3.5321725	-2.6827672	-4.1277914	-3.0856142
ENST00000568079	ZFP1	-3.568438	-2.8204496	-3.6611225	-2.9262718
ENST00000581196	C17orf62	0.2512151	1.085592	-0.1312636	0.6252281
ENST00000322354	SERTAD3	-3.3518845	-2.6101723	-3.4294039	-2.7392325
ENST00000330459	KPNA2	3.5743798	4.1830411	2.7636807	3.3624314
ENST00000360379	SPRR2D	-3.3265535	-2.6895082	-3.7600224	-2.9547583
ENST00000373079	NAP1L3	1.2251995	1.9059498	-0.1443679	0.5925741
ENST00000372115	MUTYH	-3.0483606	-2.2939049	-3.3016192	-2.5428814
ENST00000344387	ZNF197	-2.2137775	-1.5795596	-2.6886663	-2.0598341
ENST00000345868	SPO11	-2.9898014	-2.2655887	-3.9455887	-2.9141056
ENST00000507294	PPP2R2C	-1.1792684	1.2098815	-2.0864536	-0.4611138
ENST00000348417	IFT122	-2.9320023	-2.2612684	-2.9953105	-2.3873701
ENST00000329559	NDUFB1	-3.2145558	-2.6264921	-3.6397253	-2.9502279
ENST00000326407	ITM2C	0.6834172	1.3948529	0.3089933	1.0025417
uc002erg.1	BC007896	-1.8564956	-1.1868319	-2.87777	-2.2091263
ENST00000382850	NEDD4L	0.9694311	1.7420021	0.9969387	1.6789842
ENST00000439096	BBC3	0.6382928	1.2561887	0.1651886	0.8042583
ENST00000215855	CRYBB3	1.9172154	3.3936471	1.4026306	2.0694236
NM_001206654	NCOR2	-1.9793629	-1.0818287	-2.4059795	-1.6042089
ENST00000490491	PMF1-BGLAP	-2.505366	-1.6486357	-2.5856864	-1.7441193
ENST00000415430	VSIG1	-2.4484653	-1.6204327	-2.8427957	-2.0044181

ENST00000597937	EPS15L1	3.5403272	6.1699164	2.9442081	5.693607
ENST00000374867	NRP1	4.5547769	5.255125	4.1555021	4.7598423
ENST00000641816	AC005787.5	-0.1047507	0.7505663	-0.6239267	0.446197
ENST00000358907	R3HDM2	-3.4117883	-2.555508	-3.7753703	-2.7920176

Supplementary Table 5. Significance GO terms of DE gene of up regulated-methylation

GO.ID	Term	Ontology	Count	P value	FDR	Enrichment Score	Gene Ratio	GENES
GO:0007166	cell surface receptor signaling pathway	Biological process	82	8.59108E-06	0.033359979	5.065952268	0.269736842	ADORA2B//HRAS//CARD11//SHC3//DOK5//NRG1//LTBP1//ID1//PML//PARD3//SKIL//TP53//HOMER1//FMR1//HES7//PPP3CA//DHH//FN1//GRB10//A//FGF5//POLR2F//FGF16//POSTN//NDRG4//SPHK1//GDF3//CPE//CSNK1G3//RNF146//RSPO3//CCNE1//SMURF2//CXCL1//CXCL8//SUFU//PHB//DKK1//SIX3//EGR1//CDKN2B//CDIP1//TMSB4X//PLVAP//TNFSF15//FBN2//FCER1A//CYFIP2//PLAUR//GPC1//NF2//AR//CILP//SESN3//PTPRE//ZFYVE27//DDIT4//EFNB3//NFKBIZ//EIF2B4//KDM6A//CHRNA5//CD44//IFI27//ISG20//SNAI1//CXCL6//CXCL5//EDN1//NOD1//SEMA6C//SEMA4F//SEMA3B//ANKRD6//BCL2A1//ZMYND8//PLXNA1//ATF3//TIMP3//NETO2//4EYA1//TNFRSF10D
GO:0048522	positive regulation of cellular process	Biological process	126	2.23105E-05	0.033359979	4.651491637	0.414473684	NOD1//ADORA2B//NRG1//FGF5//FMR1//HRAS//PLAUR//FGF16//PLVAP//BCL2L13//PML//TNFSF15//CNOT4//TP53//ERCC6//MAPK8IP3//EDN1//FN1//HILPDA//AR//LAMC2//ATF3//PLAC8//RPS15A//CXCL5//TSPYL5//NOLC1//HOMER1//SNAI1//SPHK1//COL26A1//MGARP//GDF3//STARD4//DKK1//APC//PHB//NDRG4//ENC1//POSTN//SESN3//KIF26B//RSPO3//N6AMT1//SEMA6C//SEMA4F//PPP3CA//SEMA3B//CDKN2B//CARD11//GRB10//CLIP1//EFNB3//CXCL8//NHLRC1//PARD3//ATAD2//MAPRE2//ID1//CENPV//RXFP3//OPN1LW//CD44//HPSE//ADCY4//DDX20//ALDH1A3//TFPT//KCNMA1//PLEKHN1//BCLAF1//LITAF//BLID//NENF//DOK5//TMSB4X//GPR62//PTX3//NF2//FBN2//PDLIM7//EYA1//ZFYVE27//PFKFB3//ANAPC5//CYFIP2//EGR1//NC

								OA6//SS18L1//SMARCA2//NR2F2//BRPF1//SETD7//ZNF516//MAFB//ERG//SLC40A1//MYCL//PRDM10//RFX2//SIX3//MLYCD//ANKRD6//ABCC4//PRMT5//MYH10//PANX1//PLXNA1//SKIL//ZMYND8//SETMAR//PARP16//CSNK1G3//SMURF2//RNF146//METRNL//DDIT4//CCNE1//RAB15//CPNE5//TIMP3//POMT1//ATXN3//NFKBIZ//CYP19A1//GPC1
GO:0007399	nervous system development	Biological process	65	2.85222E-05	0.033359979	4.544816608	0.213815789	MYH10//DDIT4//NR2F2//SUFU//KDM6A//TP53//PARD3//SEMA6C//SEMA4F//EFNB3//GPC1//GRB10//SHC3//DOK5//SEMA3B//PDLIM7//NRG1//AK4//NCOA6//ID1//UFM1//PPP3CA//SIX3//NDRG4//CCIN//SPHK1//CD9//SCN8A//LAMC2//FGF5//ENC1//FKBP4//DKK1//EIF2B4//NF2//SS18L1//MAFB//NDUFS3//ALDH1A3//EFHC1//BCL2A1//ERCC6//NRBP2//EPB41L3//MAPK8IP3//ZFYVE27//DHH//MYCL//EYA1//FN1//KIF26A//PRMT5//SGK1//HES7//PLXNA1//SKIL//FMR1//ZMYND8//CYFIP2//CPNE5//POSTN//CXCL1//APC//ATXN3//SMARCA2
GO:0070848	response to growth factor	Biological process	28	3.04398E-05	0.033359979	4.516558915	0.092105263	ID1//PML//PARD3//SKIL//TP53//FGF5//POLR2F//FGF16//SMURF2//EGR1//GDF3//CDKN2B//DKK1//GRB10//FBN2//LTBP1//SPHK1//GPC1//POSTN//CXCL8//CD44//ZFYVE27//DDIT4//DOK5//CYFIP2//FNDC4//EDN1//CILP
GO:0071363	cellular response to growth factor stimulus	Biological process	27	3.90904E-05	0.033359979	4.407930023	0.088815789	ID1//PML//PARD3//SKIL//TP53//FGF5//POLR2F//FGF16//SMURF2//EGR1//GDF3//CDKN2B//DKK1//GRB10//FBN2//LTBP1//SPHK1//GPC1//POSTN//CXCL8//CD44//ZFYVE27//DDIT4//DOK5//CYFIP2//EDN1//CILP
GO:0032989	cellular component morphogenesis	Biological process	29	4.20639E-05	0.033359979	4.376090073	0.095394737	ZPBP//RFX2//PARD3//SEMA6C//SEMA4F//EFNB3//GPC1//GRB10//MYH10//SHC3//DOK5//SEMA3B//PDLIM7//LAMC2//EPB41L3//CD9//ZFYVE27//FN1//SGK1//PLXNA1//SKIL//ID1//PPP3CA//SS18L1//EDN1//CYFIP2//CPNE5//POSTN//CD44

GO:0008630	intrinsic apoptotic signaling pathway in response to DNA damage	Biological process	9	4.33828E-05	0.033359979	4.362682715	0.029605263	CDIP1//PML//DDIT4//TP53//CD44//SNAI1//SKIL//ERCC6//BCL2A1
GO:0014032	neural crest cell development	Biological process	8	4.39464E-05	0.033359979	4.357076598	0.026315789	SEMA6C//SEMA4F//SEMA3B//FN1//EDN1//NRG1//TCOF1//NOLC1
GO:0050789	regulation of biological process	Biological process	231	4.64238E-05	0.033359979	4.33325923	0.759868421	ERCC6//ATRIP//CDKN2B//CCNE1//PKIG//DDX20//AEBP1//EDN1//EGR1//ERF//FHL2//DKK1//EID1//ID1//IFI27//AR//ATF3//SUFU//PHB//ZNF248//SMURF2//SKIL//SMARCA2//SNAI1//NR2F2//TP53//FGF5//NRG1//HRAS//SHC3//PLVAP//FGF16//RPL7//RPL27//RPS15A//RPS20//NOD1//ADORA2B//CNOT4//CELF2//FMR1//EPB41L3//SGK1//KIF26A//LITAF//HILPDA//FN1//PML//PTPN13//PLAUR//POSTN//NF2//GAA//AK4//CARD11//CYP19A1//UBE2D2//SPHK1//PRMT5//ZMYND8//ZNF776//ZNF761//ZNF117//ZNF26//ZNF138//UTP4//ZNF845//ZNF516//MAFB//ZNF256//ERG//ATAD2//ZNF774//RFX2//NFKBIZ//HES7//ZNF276//ZNF518A//TCOF1//EIF2B4//NOLC1//BCL2L13//TNFSF15//CFI//APC//CGREF1//CXCL8//PLXNA1//CHRNA5//CSNK1G3//TOM1L2//METRNL//CXCL1//NENF//FAM13B//PI4KA//OPN1LW//CXCL6//CXCL5//NDRG4//TNFRSF10D//RABEP1//FCER1A//DOK5//LTBP1//PARD3//GPR62//ADCY4//RXFP3//ADM2//HOMER1//CPE//PPP3CA//DHH//TMSB4X//MAPK8IP3//LAMC2//PLAC8/TSPYL5//ZNF503//CD9//GRB10//MYH10//POLR2F//BCL2A1//SNX13//GDF3//NHLRC1//PRDM10//UTY//HPSE//KDM6A//PC//CILP//SPINK1//DDIT4//ATXN3//COL26A1//MGARP//STARD4//MLYCD//SPOCK3//TIMP3//TFPI2//ENC1//FKBP4//RNF146//RSPO3//SESN3//NRBP2//RAB15//DPH7//NCOA6//KCNMB4//MCTP1//KIF26B//SIX3//N6AMT1//NDUFS3//SEMA6C//S

								EMA4F//SEMA3B//SGMS2//MGP//FBN2//CYFIP2//KANK3//CLGN//PARP16//MAPRE2//CLIP1//EFNB3//CENPV//POLR3H//POLR1C//DENND3//PANX1//FRMD6//CD44//UFM1//CDIP1//APOM//SLC40A1//CATSPER2//TMEM37//KCNMA1//SCN8A//VPS18//ASB1//EXTL2//GPC1//PTGS1//ALDH1A3//TFPT//PLEKHN1//BCLAF1//HYPK//ATOX1//BLID//SETMAR//PTX3//ISG20//TREML2//TXNRD3//MYCL//EYA1//PDLIM7//LRRC17//ZFYVE27//PFKFB3//ANAPC5//PHC3//SS18L1//BRPF1//SETD7//ANKRD6//PTPRE//SYNGR1//ABCC4//FNDC4//EFHC1//CABLES1//CERS1//LIG3//ADAMTS18//PYCR1//PDE4DIP//CPNE5//POMT1//NETO2//EXOC2
GO:0050794	regulation of cellular process	Biological process	217	4.97337E-05	0.033359979	4.303349447	0.713815789	ERCC6//ATRIP//CDKN2B//CCNE1//PKIG//DDX20//AEBP1//EDN1//EGR1//ERF//FHL2//DKK1//EID1//ID1//IFI27//AR//ATF3//SUFU//PHB//ZNF248//SMURF2//SKIL//SMARCA2//SNAI1//NR2F2//TP53//FGF5//NRG1//HRAS//SHC3//PLVAP//FGF16//NOD1//ADORA2B//CELF2//FMR1//EPB41L3//SGK1//KIF26A//FN1//PML//PTPN13//PLAUR//POSTN//NF2//AK4//CARD11//UBE2D2//SPHK1//PRMT5//ZMYND8//ZNF776//ZNF761//ZNF117//ZNF26//ZNF138//UTP4//ZNF845//ZNF516//MAFB//ZNF256//ERG//ATAD2//ZNF774//RFX2//NFKBIZ//HES7//ZNF276//LITAF//ZNF518A//TCOF1//EIF2B4//NOLC1//BCL2L13//TNFSF15//CNOT4//APC//CGREF1//CXCL8//PLXNA1//CHRNA5//CSNK1G3//TOM1L2//METRNL//CXCL1//NENF//FAM13B//PI4KA//OPN1LW//CXCL6//CXCL5//NDRG4//TNFRSF10D//RABEP1//FCER1A//DOK5//LTBP1//PARD3//GPR62//ADCY4//RXFP3//ADM2//HOMER1//CPE//PPP3CA//DHH//TMSB4X//MAPK8IP3//HILPDA//LAMC2//PLAC8//RPS15A//TSPYL5//ZNF503//CD9//GRB10//POLR2F//BCL2A1//S

	NX13//GDF3//SPINK1//CYP19A1//DDIT4//ATXN3//COL26A1//MGARP//STARD4//MLYCD//SPOCK3//TIMP3//TFPI2//ENC1//FKBP4//RNF146//RSPO3//SESN3//NRBP2//RAB15//DPH7//MCTP1//KIF26B//SIX3//N6AMT1//NDUFS3//SEMA6C//SEMA4F//SEMA3B//CYFIP2//KANK3//CLGN//PARP16//MAPRE2//CLIP1//EFNB3//NHLRC1//CENPV//DENND3//FRMD6//CD44//UFM1//CDIP1//HPSE//SLC40A1//APOM//CATSPER2//TMEM37//KCNMA1//SCN8A//VPS18//ASB1//FBN2//EXTL2//GPC1//PTGS1//ALDH1A3//TFPT//PLEKHN1//BCLAF1//HYPK//ATOX1//BLID//CILP//SETMAR//PTX3//TXNRD3//MYCL//EYA1//PDLIM7//LRRC17//ZFYVE27//PFKFB3//ANAPC5//PHC3//NCOA6//SS18L1//BRPF1//SETD7//PRDM10//ANKRD6//PTPRE//KCNMB4//SYNGR1//ABCC4//MYH10//PANX1//EFHC1//CABLES1//CERS1//KDM6A//ISG20//LIG3//ADAMTS18//PYCR1//PDE4DIP//CPNE5//POMT1//NETO2

Supplementary Table 6. Significance GO terms of DE gene of up regulated-mRNA

GO.ID	Term	Ontology	Count	P value	FDR	Enrichment Score	Gene. Ratio	GENES
GO:0007166	cell surface receptor signaling pathway	Biological process	46	8.18621E-05	0.241922586	4.08691733	0.294871795	CARD11//FGFR4//SHC3//DOK5//NRG1//HOMER1//HES7//FN1//FGF5//FGF16//POSTN//NDRG4//SPHK1//GDF7//GDF3//CPE//CSNK1G3//RSPO3//CCNE1//CXCL8//SUFU//DKK1//SIX3//EGR1//CDKN2B//TMSB4X//TNFSF15//FBN2//COL4A5//FCER1A//GPC1//AR//SESN3//DDIT4//KDM6A//CHRNA5//SNAI1//CXCL6//CXCL5//SEMA6C//SEMA4F//BCL2A1//PLXNA1//TIMP3//NETO2//TNFRSF10D
GO:0048568	embryonic organ development	Biological process	13	9.8395E-05	0.241922586	4.007026953	0.083333333	SUFU//NDRG4//ALDH1A3//SIX3//GDF3//MAFB//FBN2//CXCL8//KDM6A//RSPO3//SNAI1//NR2F2//FBXW8
GO:0006928	movement of cell or subcellular component	Biological process	36	0.000102815	0.241922586	3.987945511	0.230769231	CENPV//SEMA6C//SEMA4F//MYH10//DDIT4//NR2F2//KIF26A//GDF7//GPC1//SHC3//DOK5//MGARP//CD9//PLXNA1//POSTN//NDRG4//FGFR4//SNAI1//SDC3//NRG1//FGF5//TMSB4X//FGF16//SPHK1//MCTP1//CXCL8//CXCL6//CXCL5//FN1//TNFRSF10D//SLC16A3//MREG//SRI//SIX3//SPOCK3//CLDN7
GO:0040011	locomotion	Biological process	32	0.000215292	0.244447179	3.666972735	0.205128205	CENPV//SEMA6C//SEMA4F//MYH10//DDIT4//NR2F2//CXCL8//CXCL6//CXCL5//GDF7//GPC1//SHC3//DOK5//CD9//PLXNA1//POSTN//NDRG4//FGFR4//SNAI1//SDC3//NRG1//FGF5//TMSB4X//FGF16//SPHK1//MCTP1//FN1//TNFRSF10D//SLC16A3//SIX3//SPOCK3//CLDN7
GO:0009888	tissue development	Biological process	33	0.000215482	0.244447179	3.666588391	0.211538462	MGP//DKK1//SNAI1//SEMA6C//SEMA4F//HES7//SUFU//KDM6A//NDRG4//SIX3//ALDH1A3//SLC40A1//NRG1//AR//GDF3//NR2F2//CD9//GDF7//FBN2//TST//KRT7//EGR1//FN1//MYCL//LRRC17//HOMER1//HPSE//MYH10//FHL2//PLXNA1//RSPO3//POSTN//GPC1

GO:0042994	cytoplasmic sequestering of transcription factor	Biological process	3	0.000286959	0.244447179	3.542179449	0.019230769	TMSB4X//SUFU//SRI
GO:0048646	anatomical structure formation involved in morphogenesis	Biological process	22	0.000370473	0.244447179	3.431243292	0.141025641	FN1//CXCL8//RFX2//DKK1//SNAI1//HES7//SUFU//KDM6A//RSPO3//ALDH1A3//CD9//GDF7//MAFB//GPC1//GDF3//SPHK1//AR//MYH10//HPSE//SLC40A1//FBN2//FHL2
GO:0050789	regulation of biological process	Biological process	122	0.000376816	0.244447179	3.423870293	0.782051282	ATRIP//CDKN2B//CCNE1//AEBP1//EGR1//ERF//FHL2//DKK1//AR//SUFU//ZNF248//SMARCA2//SNAI1//NR2F2//FGF5//FGFR4//NRG1//SHC3//FGF16//RPL7//CELF2//KIF26A//FN1//POSTN//CARD11//SPHK1//ZNF776//ZNF117//ZNF26//UTP4//MAFB//ERG//ZNF774//RFX2//ZNF397//HES7//ZNF276//TNFSF15//SRI//CGREF1//CXCL8//CLDN7//PLXNA1//CHRNA5//CSNK1G3//TOM1L2//FAM13B//CXCL6//CXCL5//NDRG4//TNFRSF10D//RABEP1//FCER1A//DOK5//ADCY4//HOMER1//CPE//TMSB4X//PLAC8//TSPYL5//ZNF503//CD9//MYH10//BCL2A1//SNX13//GDF3//NHLRC1//PRDM10//UTY//HPSE//KDM6A//PC//SPINK1//DDIT4//MGARP//GDF7//SPOCK3//TIMP3//TFPI2//ENC1//RSPO3//SESN3//NRBP2//DPH7//KCNMB4//MCTP1//SIX3//SEMA6C//SEMA4F//MGP//FBN2//CLGN//CENPV//POLR3H//PANX1//UFM1//APOM//SLC40A1//KCNMA1//COL4A5//GPC1//ALDH1A3//TFPT//PLEKHN1//HYPK//BLID//SETMAR//PTX3//TXNRD3//MYCL//LRRC17//ANAPC5//SS18L1//SYNGR1//FNDC4//FBXW8//CERS1//LIG3//ADAMTS18//POMT1//NETO2//EXOC2
GO:0050794	regulation of cellular process	Biological process	115	0.000385189	0.244447179	3.414326241	0.737179487	ATRIP//CDKN2B//CCNE1//AEBP1//EGR1//ERF//FHL2//DKK1//AR//SUFU//ZNF248//SMARCA2//SNAI1//NR2F2//FGF5//FGFR4//NRG1//SHC3//FGF16//CELF2//KIF26A//FN1//POSTN//CARD11//SPHK1//ZNF776//ZNF117//ZNF26//UTP4//MAFB//ERG//ZNF774//RFX

								2//ZNF397//HES7//ZNF276//TNFSF15//CGREF1//CXCL8//CLDN7//PLXNA1//CHRNA5//CSNK1G3//TOM1L2//FAM13B//CXCL6//CXCL5//NDRG4//SRI//TNFRSF10D//RABEP1//FCER1A//DOK5//ADCY4//HOMER1//CPE//TMSB4X//PLAC8//TSPYL5//ZNF503//CD9//BCL2A1//SNX13//GDF3//SPINK1//DDIT4//MGARP//GDF7//SPOCK3//TIMP3//TFPI2//ENC1//RSPO3//SESN3//NRBP2//DPH7//MCTP1//SIX3//SEMA6C//SEMA4F//CLGN//NHLRC1//CENPV//UFM1//HPSE//SLC40A1//APOM//KCNMA1//FBN2//COL4A5//GPC1//ALDH1A3//TFPT//PLEKHN1//HYPK//BLID//SETMAR//PTX3//TXNRD3//MYCL//LRRC17//ANAPC5//SS18L1//PRDM10//KCNMB4//SYNGR1//MYH10//PANX1//FBXW8//CERS1//KDM6A//LIG3//ADAMTS18//POMT1//NETO2
GO:0048870	cell motility	Biological process	29	0.000433734	0.244447179	3.362776943	0.185897436	CENPV//SEMA6C//SEMA4F//MYH10//DDIT4//NR2F2//CD9//PLXNA1//POSTN//NDRG4//FGFR4//GPC1//SNAI1//SDC3//NRG1//FGF5//TMSB4X//FGF16//SPHK1//MCTP1//CXCL8//CXCL6//CXCL5//FN1//TNFRSF10D//SLC16A3//SIX3//SPOCK3//CLDN7
GO:0051674	localization of cell	Biological process	29	0.000433734	0.244447179	3.362776943	0.185897436	CENPV//SEMA6C//SEMA4F//MYH10//DDIT4//NR2F2//CD9//PLXNA1//POSTN//NDRG4//FGFR4//GPC1//SNAI1//SDC3//NRG1//FGF5//TMSB4X//FGF16//SPHK1//MCTP1//CXCL8//CXCL6//CXCL5//FN1//TNFRSF10D//SLC16A3//SIX3//SPOCK3//CLDN7

Supplementary Table 7. GO analysis shows the downregulated m6A

GO.ID	Term	Ontology	Count	P value	FDR	Enrichment.Score	Gene.Ratio	GENES
GO:0002028	regulation of sodium ion transport	Biological process	4	9.34184E-05	0.588558107	4.029567509	0.078431373	PKP2//NEDD4L//NOS1//FXYD3
GO:0002934	desmosome organization	Biological process	2	0.000317979	0.588558107	3.49760194	0.039215686	PKP3//PKP2
GO:0045981	positive regulation of nucleotide metabolic process	Biological process	3	0.000333508	0.588558107	3.476893844	0.058823529	NOS1//PFKFB1//ENTPD5
GO:1900544	positive regulation of purine nucleotide metabolic process	Biological process	3	0.000333508	0.588558107	3.476893844	0.058823529	NOS1//PFKFB1//ENTPD5
GO:0010038	response to metal ion	Biological process	6	0.000441203	0.622890414	3.35536153	0.117647059	MT1A//SPARC//BSG//SYT10//BRAF//NEDD4L
GO:1902305	regulation of sodium ion transmembrane transport	Biological process	3	0.000722348	0.849842892	3.141253285	0.058823529	NEDD4L//NOS1//FXYD3
GO:0006513	protein monoubiquitination	Biological process	3	0.000859895	0.851177312	3.065554466	0.058823529	PCGF5//NEDD4L//PEX12
GO:0010562	positive regulation of phosphorus metabolic process	Biological process	10	0.001085224	0.851177312	2.964480633	0.196078431	BRAF//IGFBP6//NKX3-1//NOS1//STRADA//PFKFB1//PSRC1//ENTPD5//CACUL1//ADGRF5
GO:0045937	positive regulation of phosphate metabolic process	Biological process	10	0.001085224	0.851177312	2.964480633	0.196078431	BRAF//IGFBP6//NKX3-1//NOS1//STRADA//PFKFB1//PSRC1//ENTPD5//CACUL1//ADGRF5
GO:0065007	biological regulation	Biological process	45	0.001253736	0.885012535	2.90179376	0.882352941	KPNA2//NEDD4L//MEPCE//RASGRP3//BRAF//IGFBP6//NKX3-1//SPARC//NOS1//ZNF197//FOSL2/

/ZSCAN5A//PCGF5//MT1A//STRADA//TBRG4//TGFBI//PDE7A//ERAP2//ADGRF5//BSG//GP6//OR2AG1//GPR35//HTR5A//OR6K2//FGD3//CACUL1//FIBP//PKP3//PKP2//PMEPA1//SYT10//ENTPD5//PLSCR5//PSRC1//TMSB15A//MYRIP//CNN3//PFKFB1//EPS15L1//PLB1//COBL//NEFH//FXYD3

Supplementary Table 8. GO analysis shows the downregulated m6A

GO.ID	Term	Ontology	Count	P value	FDR	Enrichment. Score	Gene.Ratio	GENES
GO:0002028	regulation of sodium ion transport	Biological process	4	9.34184E-05	0.588558107	4.029567509	0.078431373	PKP2//NEDD4L//NOS1//FXYD3
GO:0002934	desmosome organization	Biological process	2	0.000317979	0.588558107	3.49760194	0.039215686	PKP3//PKP2
GO:0045981	positive regulation of nucleotide metabolic process	Biological process	3	0.000333508	0.588558107	3.476893844	0.058823529	NOS1//PFKFB1//ENTPD5
GO:1900544	positive regulation of purine nucleotide metabolic process	Biological process	3	0.000333508	0.588558107	3.476893844	0.058823529	NOS1//PFKFB1//ENTPD5
GO:0010038	response to metal ion	Biological process	6	0.000441203	0.622890414	3.35536153	0.117647059	MT1A//SPARC//BSG//SYT10//BRAF//NEDD4L
GO:1902305	regulation of sodium ion transmembrane transport	Biological process	3	0.000722348	0.849842892	3.141253285	0.058823529	NEDD4L//NOS1//FXYD3
GO:0006513	protein monoubiquitination	Biological process	3	0.000859895	0.851177312	3.065554466	0.058823529	PCGF5//NEDD4L//PEX12
GO:0010562	positive regulation of phosphorus metabolic process	Biological process	10	0.001085224	0.851177312	2.964480633	0.196078431	BRAF//IGFBP6//NKX3-1//NOS1//STRADA//PFKFB1//PSRC1//ENTPD5//CACUL1//ADGRF5
GO:0045937	positive regulation of phosphate metabolic process	Biological process	10	0.001085224	0.851177312	2.964480633	0.196078431	BRAF//IGFBP6//NKX3-1//NOS1//STRADA//PFKFB1//PSRC1//ENTPD5//CACUL1//ADGRF5
GO:0065007	biological regulation	Biological process	45	0.001253736	0.885012535	2.90179376	0.882352941	KPNA2//NEDD4L//MEPCE//RASGRP3//BRAF//IGFBP6//NKX3-1//SPARC//NOS1//ZNF197//FOSL2//ZS

CAN5A//PCGF5//MT1A//STRADA//TBRG4//TGFBI//PDE7A//ERAP2//ADGRF5//BSG//GP6//OR2AG1//GPR35//HTR5A//OR6K2//FGD3//CACUL1//FIBP//PKP3//PKP2//PMEPA1//SYT10//ENTPD5//PLSCR5//PSRC1//TMSB15A//MYRIP//CNN3//PFKFB1//EPS15L1//PLB1//COBL//NEFH//FXYD3
